# Domain Movement within a Gene: A Novel Evolutionary Mechanism for Protein Diversification

**DOI:** 10.1371/journal.pone.0018819

**Published:** 2011-04-14

**Authors:** Yoshikazu Furuta, Mikihiko Kawai, Ikuo Uchiyama, Ichizo Kobayashi

**Affiliations:** 1 Department of Medical Genome Sciences, Graduate School of Frontier Sciences, University of Tokyo, Minato-ku, Tokyo, Japan; 2 Institute of Medical Science, University of Tokyo, Minato-ku, Tokyo, Japan; 3 Laboratory of Genome Informatics, National Institute for Basic Biology, Okazaki, Aichi, Japan; 4 Department of Biophysics and Biochemistry, Graduate School of Science, University of Tokyo, Minato-ku, Tokyo, Japan; University of Wyoming, United States of America

## Abstract

A protein function is carried out by a specific domain localized at a specific position. In the present study, we report that, within a gene, a specific amino acid sequence can move between a certain position and another position. This was discovered when the sequences of restriction-modification systems within the bacterial species *Helicobacter pylori* were compared. In the specificity subunit of Type I restriction-modification systems, DNA sequence recognition is mediated by target recognition domain 1 (TRD1) and TRD2. To our surprise, several sequences are shared by TRD1 and TRD2 of genes (alleles) at the same locus (chromosomal location); these domains appear to have moved between the two positions. The gene/protein organization can be represented as x-(TRD1)-y-x-(TRD2)-y, where x and y represent repeat sequences. Movement probably occurs by recombination at these flanking DNA repeats. In accordance with this hypothesis, recombination at these repeats also appears to decrease two TRDs into one TRD or increase these two TRDs to three TRDs (TRD1-TRD2-TRD2) and to allow TRD movement between genes even at different loci. Similar movement of domains between TRD1 and TRD2 was observed for the specificity subunit of a Type IIG restriction enzyme. Similar movement of domain between TRD1 and TRD2 was observed for Type I restriction-modification enzyme specificity genes in two more eubacterial species, *Streptococcus pyogenes* and *Mycoplasma agalactiae*. Lateral domain movements within a protein, which we have designated DOMO (*do*main *mo*vement), represent novel routes for the diversification of proteins.

## Introduction

A specific function of a protein molecule can often be ascribed to a specific region within the polypeptide chain called a domain [1]. The structure and sequence of a domain can diversify through different types of recombination at the DNA or RNA level [2]. In eukaryotic genes that have the exon/intron structure, exon shuffling through DNA recombination and alternative RNA splicing can lead to the reorganization of protein domains [3], [4]. Antigenic variation in some microbes and antibody formation in some organisms arise from gene conversion with various donor sequences, leading to domain diversification [5], [6].

**Figure 1:**
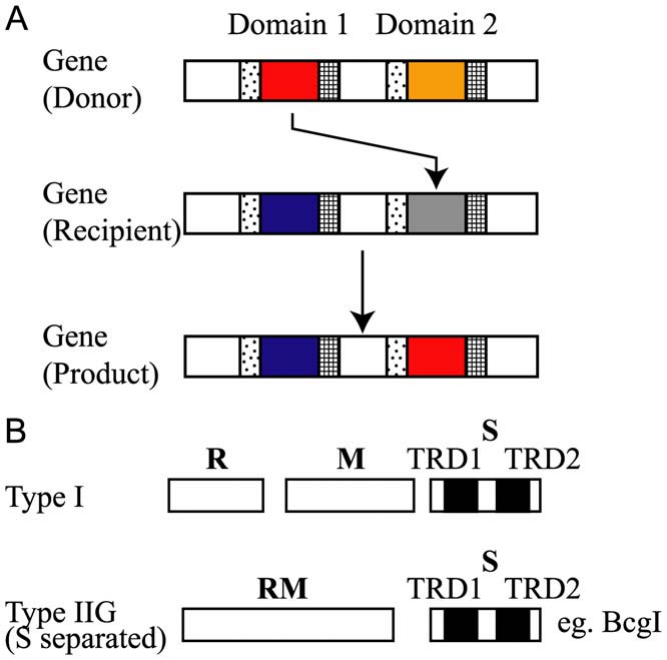
Domain movement and specificity subunits. (A) Domain movement. A specific amino acid sequence of a domain in a specific position moves to another domain in another position within the same protein (protein from the same locus) by recombination at repeat DNA sequences (hatched and dotted squares) that flank the DNA sequences for the domains in their genes. (B) Subunit organization in Type I and some Type IIG restriction modification systems. The specificity (S) subunit consists of two target recognition domains: TRD1 and TRD2. R, restriction subunit; M, modification subunit; RM, restriction modification subunit; TRD, target recognition domain.

Recognition of a specific DNA sequence by a protein can be mediated by a domain that is often called the target recognition domain (TRD); this has been studied in detail for several restriction (R) modification (M) enzymes [7]–[9]. Modification enzymes methylate a specific DNA sequence, whereas their cognate restriction enzymes cut DNA which lacks methylation at this sequence. Type I restriction modification systems consist of the R, M, and specificity (S) subunits (Fig. 1B) [10]. Their DNA sequence recognition is determined by TRD1 and TRD2 present in the S subunit, each of which recognizes half of a bipartite target sequence [11]. The region between TRD1 and TRD2 determines the distance between the two elements of the target sequence. A subclass of Type IIG RM systems consists of a similar S subunit and a polypeptide carrying both modification and restriction activities (Fig. 1B) [12].

RM systems, which limit the horizontal transfer of genes, are themselves mobile as revealed by genome comparison, genome context analyses, phylogenetic analyses and laboratory experiments [13]–[16]. Although these were discovered and studied for their ability to attack invading DNAs, their biological significance appears to extend beyond the defense function [17]. They define the specific epigenetic status of a genome by methylation of specific genome sequences in a combinatorial manner [18]. Alteration in the epigenetic status might lead to cell death by restriction enzymes [19]–[21]. This may help to maintain the genome, its epigenomic state [21], and RM systems [22].

The *Helicobacter pylori* is present in the human stomach [23] and is known to possess many diverse restriction-modification systems [24], [25]. This bacterium, which is also known for genome diversity through frequent recombination [26], provides a unique opportunity for studying the origin of diversity of target recognition domains.

In this study, we analyzed target recognition domains of restriction-modification systems in complete genome sequences of geographically diverse *H. pylori* strains. To our surprise, we found that the domain sequences themselves are mobile within a gene (Fig. 1A).

## Results

Type I RM systems contain the specificity (S) subunit that determines their recognition sequence and is necessary for both restriction and modification activities (Fig. 1B) [8]. Their recognition sequences are asymmetric and bipartite, for example, 5′GAA(N)_6_RTCG for EcoR124I. These are recognized by two features of S: the central repeat region and the two target recognition domains (TRDs) TRD1 and TRD2 (Fig. 1B). TRD1 recognizes the 5′ half of the recognition sequence (5′GAA), whereas TRD2 recognizes the 3′ half (RTCG). The central repeat determines the relative distance between these two component sequences. For example, the change in the number of the central 10-bp repeat of EcoR124I from 2 to 3 changes the recognition sequence from GAA(N)_6_RTCG to GAA(N)_7_RTCG [27]. Recombination of TRD1 sequences and TRD2 sequences was reported to create novel target specificity [28]–[30]. Sharing of TRD sequences between two S paralogs has already been reported, but was restricted to that between TRD1 and TRD1 or between TRD2 and TRD2 [31], [32].

### Type I S genes in *H. pylori*

*H. pylori* has been assigned many alleles of Type I specificity subunits, from *hsdS1* to *hsdS6*, based on the locus and sequence similarity of the entire ORF (open reading frame) [33]. Although their TRD regions are highly diverse, we clustered these anew, according to their conserved regions, into only 3 homology groups: Group 1 S (*hsdS1*, Fig. S1), Group 2 S (*hsdS2*, *hsdS4*, and *hsdS5*, Fig. S2), and Group 3 S (*hsdS3* and *hsdS6*, Fig. S3). We then compared members of each Group at the nucleotide sequence level.

**Figure 2:**
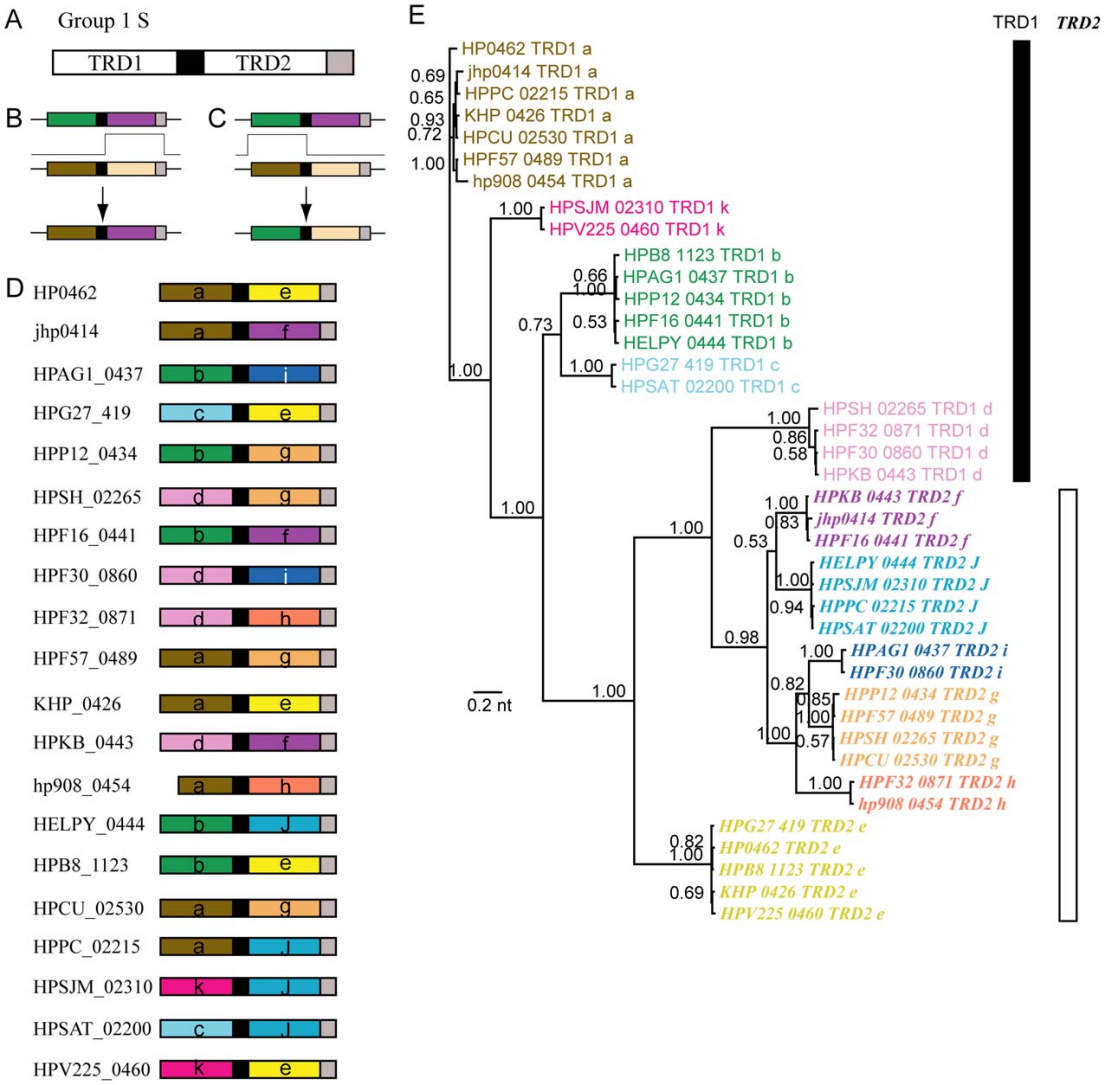
The S subunit gene of Group 1 of Type I RM systems. (A) Common organization of Group 1 S. The black and gray boxes indicate central and C-terminal conserved sequences, respectively. (B, C) Mechanisms of TRD substitution by recombination. (D) Organization in each strain. (E) Phylogenetic tree of the half TRD sequences. The color of labels of terminal nodes corresponds to the color of TRDs in Fig. 2D. The labels of TRD2 are in italic and bold. The numbers indicate posterior probabilities.

Organization of Group 1 S genes is TRD1-conserved-TRD2-conserved as illustrated in Fig. 2A. Recombination between TRD1 sequence and TRD2 sequence was observed (Fig. 2D). For example, domain sequence labeled *b* at TRD1 was paired with domain sequence labeled *i*, *g*, *f*, *j*, and *e* at TRD2. This was probably mediated by recombination in the central region and conserved flanking sequences. More specifically, the recombination events that replaced TRD2 (Fig. 2B) probably occurred at the central conserved region and at the right conserved region or the conserved region to the right of the gene. The recombination events to replace TRD1 (Fig. 2C) probably occurred at the central conserved region and at the conserved region to the left of the gene.

In the phylogenetic tree (Fig. 2E), TRD1 sequences and TRD2 sequences are clearly separated, as expected. After all, a TRD1 sequence is replaced by another TRD1 sequence, and a TRD2 sequence by another TRD2 sequence, at this locus. This tree justifies our color grouping in Fig. 2D. This pattern has also been noted for the Type I S subunit in *Staphylococcus aureus* [31], [32].

### Domain movement in S genes in Group 2 of Type I systems

**Figure 3:**
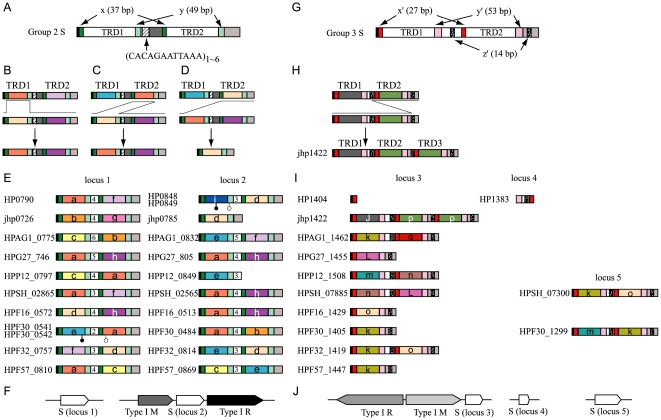
The S subunit genes of Group 2 and Group 3 Type I RM systems. (A) Common organization of Group 2 S. (B) TRD substitution by recombination between the repeats. (C) TRD movement by recombination between the repeats. (D) TRD loss by recombination between the repeats. (E) Group 2 S at loci 1 and 2. The number in the central white box indicates the copy number of the repeat sequence shown in A (above). (F) Genetic map. (G) Common organization of Group 3 S. (H) Duplication of TRD by recombination between the repeats. (I) Group 3 S at loci 3, 4, and 5. (J) Genetic map. A white circle indicates a start codon, whereas a black circle indicates a stop codon. Circles at the ends that are expected for a full-length ORF are omitted.

We encountered a deviation from this pattern when analyzing Group 2 Type I S genes (Fig. 3). These are present at two loci in all the strains (Fig. 3F). Their organization (Fig. 3A) is more complex because they carry two pairs of direct repeats; one, designated as x, is 37-bp long and the other, designated as y, is 49-bp long. TRD1 is flanked by the left x sequence and left y sequence, whereas TRD2 is flanked by the right x sequence and right y sequence. In addition to the combinatorial variations of TRD1 sequences and TRD2 sequences observed with Group 1 Type I S genes, we found that some of the sequences are shared by TRD1 and TRD2 (Fig. 3E). For example, domain sequence labeled *a* is present in TRD1 (HP0790, HPG27_746, HPSH_02865, HPF57_0810) as well as in TRD2 (HPP12_0797, HPF30_0541/HPF30_0542) at locus 1. Domain sequences labeled *b*, *c*, *d*, and *f* are also shared by TRD1 and TRD2 at locus 1. At locus 2, domain *e* appears to have moved between TRD1 and TRD2. We also found that some of these domain sequences (*a*, *b*, *c*, *d*, *e*, *f*, *h*) are shared by the two loci.

**Figure 4:**
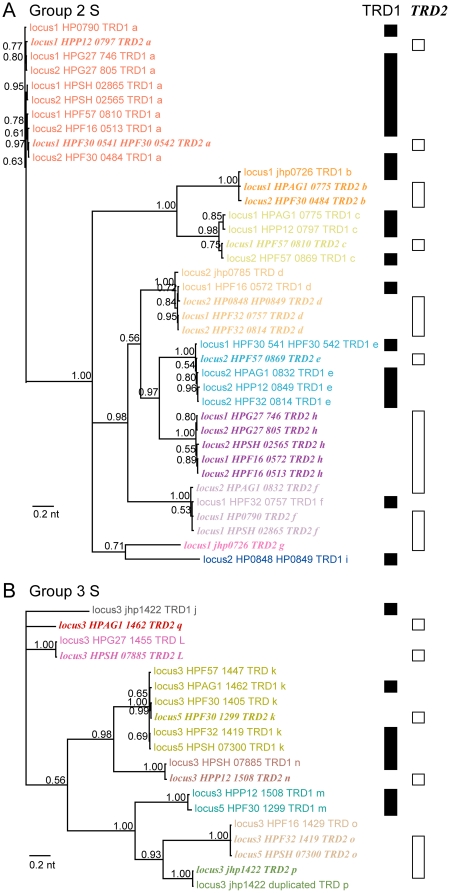
Phylogenetic trees of TRDs of S subunit genes of Group 2 and Group 3 Type I RM systems. (A) Group 2. (B) Group 3. The colors correspond to those in Fig. 3E and 3I. The labels of TRD2s are in italics and bold. The numbers indicate posterior probabilities.

Such apparent movements of domain sequences between TRD1 and TRD2 are clearly shown in their phylogenetic tree (Fig. 4A). The terminal nodes of TRD1 and TRD2 sequences are mixed, which is in contrast to those of Group 1 (Fig. 2E). This tree also justifies our homology-based color grouping of the TRD sequences.

Figure 3B illustrates a likely mechanism for replacement of one sequence at TRD1 by another sequence through recombination events between the two left x sequences and between the two left y sequence, with retention of the original central repeats and TRD2. The central region may be substituted if the right x sequence is used instead of the left y sequence. The right x sequence (or the left y sequence) and right y sequence are supposed to be used for recombination events to replace sequences in TRD2.

Figure 3C shows the likely recombination mechanism underlying the movement of a domain sequence from TRD2 to TRD1. The left recombination occurs between the right x sequence of the upper gene (allele) and the left x sequence of the lower gene, whereas the right recombination takes place between the right y sequence of the upper gene and the left y sequence of the lower gene. The two repeat pairs help in the movement of a sequence from TRD2 to TRD1 by recombination based on their sequence identity.

The role of these repeat sequences in recombination was supported by analysis of an aberrant gene. One of the Group 2 S alleles had only one TRD (jhp0785 at locus 2, Fig. 3E, locus 2, 2nd row). Sequence examination suggests the mechanism illustrated in Fig. 3D: recombination between the right x sequence of the upper allele and the left x sequence of the lower allele. If the domain sequence labeled *d* was present at TRD1, recombination could occur between the left y sequence of this allele and the right y sequence of another allele. This argument indirectly supports the involvement of repeat-mediated recombination in the domain sequence movements. A Group 2 S allele (HPP12_0849, Fig. 3E, locus 2, 5th row) also had only one TRD, but this could be explained by the simple deletion of TRD2.

Some S subunits carrying only one TRD are known to be active through dimerization [34], [35]. Although these two deletion alleles show no detectable sign of further decay, we do not know whether these are active or not. If active, the above deletion mechanism through repeat-mediated recombination represents a novel route for the variation of sequence recognition by Type I RM systems.

The S alleles also have a variable number of 12-bp repeats (5′CACAGAATTAAA) in the central region (Fig. 3A, lower). The copy number of the central repeats is variable among the alleles from 2 (24 bp, 8 amino acids) to 6 (72 bp, 24 amino acids). Because the central repeat determines the relative distance between these two component sequences, this suggests further variation in the recognition sequences. This variation may also be generated by recombination at the same sequences of x and y. Here, the unit of <y−(central region)−x> is likely the unit of movement within a locus and between loci. This hypothesis is consistent with absence of both the x, y repeats and the central long repeats from Group 1 S genes.

### Domain movement in S genes in Group 3 of Type I systems

Group 3 genes appear to be the most complex (Fig. 3G–J). They are spread across three loci (Fig. 3J): one locus (locus 3) with R and M genes and two loci (locus 4 and locus 5) by themselves. Group 3 alleles carry three pairs of repeat sequences (x′ (27 bp), y′ (53 bp) and z′ (14 bp)(Fig. 3G)), whereas Group 2 alleles carry two pairs (Fig. 3A). Domain sequences such as *k* and *o*, apparently moved between TRD1 and TRD2 and between locus 3 and locus 5. These movements can be explained by recombination through the same mechanism based on the identity of these x′ and y′ repeat sequences, as shown in Fig. 3BC. The phylogenetic tree of the TRD sequences (Fig. 4B) shows the validity of our homology grouping and supports movement between TRD1 and TRD2.

All the deletion forms of Group 3 alleles retaining only one TRD (HPG27_1455, HPF16_1429, HPF30_1405 and HPF57_1447, in the 4rd, 7th, 8th, and 10th rows in Fig. 3I) appear to have been formed through recombination between the x′ or y′ sequences by a mechanism similar to that illustrated in Fig. 3D.

One Group 3 S allele carried three, rather than two, TRDs (jhp1422, Fig. 3I, locus 3, 2nd row): one TRD1 and two TRD2s. Fine genome comparison suggested a possible mechanism (Fig. 3H) of unequal recombination between the left z′ repeat and right z′ repeat to duplicate TRD2. Although there is no sign of decay in the sequence of this gene, we do not know about the activity of this protein or the nature of its recognition sequence, if any.

Strain 26695 (Fig. 3I, 1st row) carries broken paralogs at loci 3 and 4. HP1404 (Fig. 3I, locus 3, 1st row) retained only the N-terminal end, whereas HP1383 (Fig. 3I, locus 4, 1st row) appears to be a remnant of the central part. The genomic region between these two broken paralogs is inverted in strain 26695. Detailed sequence analysis there suggests that this inversion event likely led to the breakage of these two [36].

### Movement of domains in S genes of a Type IIG RM system

**Figure 5:**
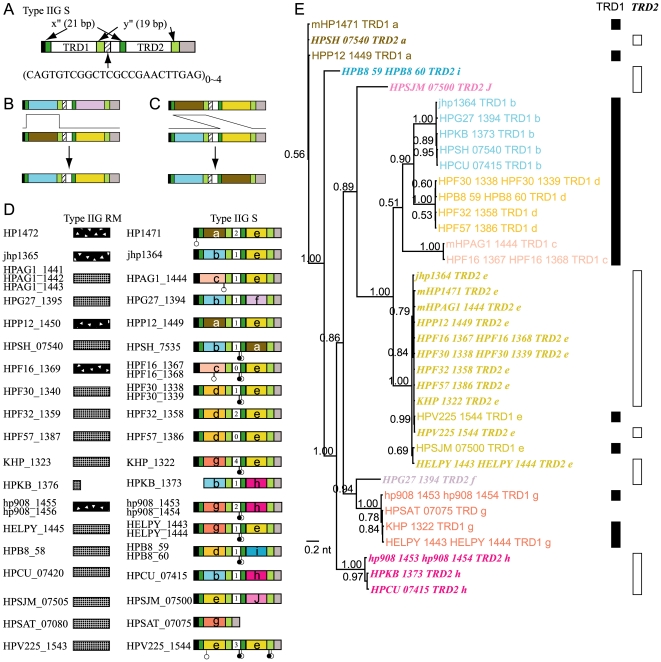
Diversity in the S subunit of a Type IIG RM system. (A) Common organization of the S subunit. (B) TRD substitution by recombination between the repeats. (C) TRD movement by recombination between the repeats. (D) Organization of the RM subunit and S subunit genes in each strain. The number in the central white box indicates the copy number of the repeat sequence shown in the lower part of Fig. 5A (above). Other symbols are the same as those used in Fig. 3. (E) Phylogenetic tree of TRDs. The colors correspond to those in Fig. 5D. The labels of TRD2s are in italics and bold. The numbers indicate posterior probabilities.

Some subclasses of Type IIG RM systems carry an S subunit homologous to that in the Type I RM system and separate from the RM subunit (Fig. 1B) [12]. A Type IIG RM system was found conserved in all the strains examined (Fig. 5, Fig. S4). Sequence diversity was observed in the S gene and the RM gene (Fig. 5D).

Organization of the S genes (Fig. 5A) is very similar to that of Group 2 Type I S genes (Fig. 2A). There are two repeat pairs: one (x″) of 21 bp and the other (y″) of 19 bp. There are 0 to 4 copies of a 24 bp sequence (5′ CAGTGTCGGCTCGCCGAACTTGAG) repeated in tandem between these (Fig. 5A).

In addition to the combinatorial variation of the sequences in the two TRD regions (Fig. 5BD), TRD sequence *a* is present in TRD1 and TRD2, probably through recombination involving the above-mentioned repeat sequences (Fig. 5CD). TRD sequence *e* is also present in TRD1 and TRD2. It is present in both TRD1 and TRD2 in one allele (HPV225_1544, Fig. 5D, the last row). A gene with a single TRD was also found (strain HPSAT_07075, Fig. 5D, the penultimate row), which suggests role for the repeat sequences in recombination as in Type I S single TRD genes (Fig. 3EI).

The copy numbers of the tandem repeat between the two TRDs also varied from 0 to 4 (Fig. 5D). This central region may have moved helped by the flanking y″ and x″ sequences.

The phylogenetic tree supports our homology grouping and our concepts of reassortment and movement (Fig. 5E).

Thus, this RM system is also predicted to exhibit have variation in the recognition sequences. In addition, two alleles were distinguished in the linked RM subunit gene, which has no mutual sequence similarity for the entire ORF (Fig. 5D). Together with the diversity in the S subunit, this system may have created great variation in sequence recognition and other properties.

### Intra-locus movement or two inter-locus movements?

These results suggested that a sequence can move between TRD1 and TRD2. Does this take place as one event? Alternatively, is it a result of multiple inter-locus events?

For Group 2 and Groupd 3 Type I S genes present in multiple loci (Fig. 3), we cannot exclude the latter possibility. However, for the Type IIG S gene present in only one locus (Fig. 5), the latter possibility is unlikely. The movement of the sequence is likely take place between two alleles of the same locus.

**Figure 6:**
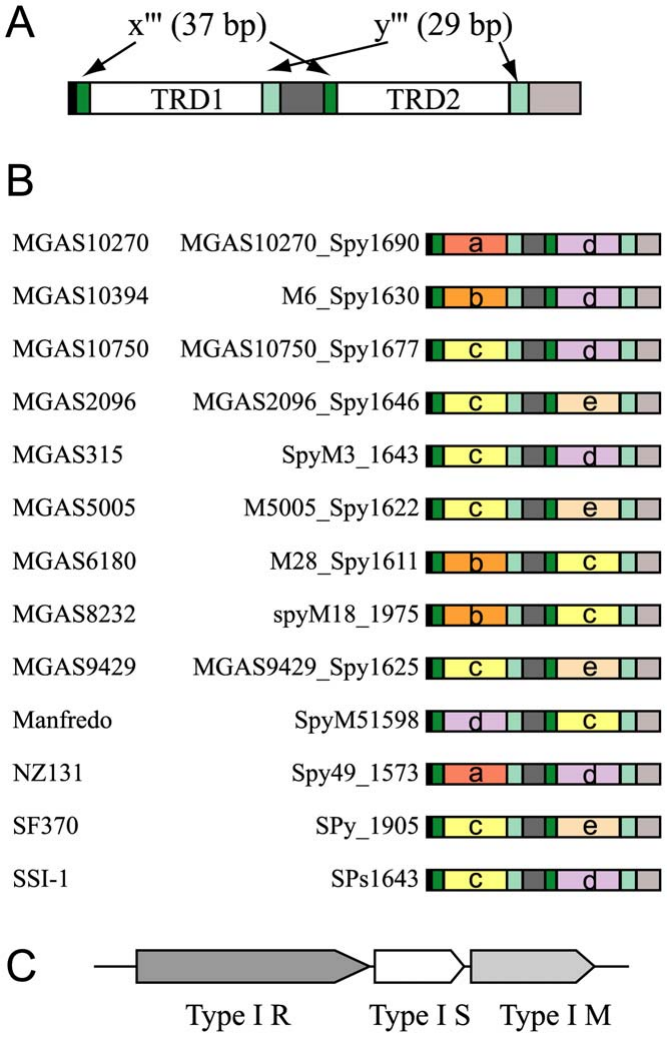
Domain movement in *Streptococcus pyogenes*. (A) Common organization of Type I S genes at a locus. (B) Type I S genes at a single locus. The leftmost label represents a strain name. (C) Genetic map.

We searched for more examples of domain movement in S genes, both Type I and Type IIG, in other species by comparing S genes within the same species. Examples were detected in the single Type I locus of *Streptococcus pyogenes* (Fig. 6). Domain sequence movement was observed for sequences *c* and *d* by comparison between 13 complete genomes (Fig. 6B). TRDs are flanked by repeat sequences, x‴ of 37 bp and y‴ of 29 bp (Fig. 6A), which supports the mechanism of domain movement by recombination at the flanking repeats. This result indicates that, in Type I S gene, a sequence can move between TRD1 and TRD2 within the same locus.

In two strains (56323 and PG2) of *Mycoplasma agalactiae*, a family of Type I S genes are found at three loci. Their TRDs are flanked by 37-bp and 34-bp repeat sequences. A sequence was found shared by TRD2 at locus 1 (MAGa6280) in strain 5632, TRD1 at locus 3 (MAGa6340) in strain 5632, and TRD2 at locus 1 (MAG5640). MAGa6310 had three TRDs while MAG5720 had one TRD, likely through recombination at the repeats.

## Discussion

Sequence analyses of allelic diversities in target recognition domains of restriction-modification systems in various *H. pylori* complete genomes revealed the movement of a sequence between domains in different positions within a protein coding gene. We designated such movement of the domain sequence within a protein (a locus) as DOMO (*do*main *mo*vement). DOMO defines a novel route for protein diversification.

Domain movement is probably mediated by repeat sequences flanking the TRDs. The movement of sequences between specificity genes at different loci sharing the same repeats and the deletion and duplication of the domains are likely mediated by these repeats. This hypothesis is also consistent with the observation of no DOMO in the Group 1 Type I S genes lacking the repeats. The detection of DOMO in Type I S genes with TRDs flanked by repeats in two more species also support the mechanism. The aberrant S genes with only one TRD (Fig. 3, Fig. 5) are explained by recombination at the repeat sequences resulting in a deletion. However, we do not know whether this recombination is mediated by DOMO or some other mechanisms, such as DNA replication error at the repeats on the same DNA molecule.

Because we analyze complete genome sequences, we are certain that a single locus is involved in DOMO for Type IIG locus in *H. pylori* and for Type IS locus in *Streptococcus pyogenes*. This indicates that DOMO can take place within the same locus at least in these cases.

We do not know whether such recombination is mediated by site-specific recombination machinery or homologous recombination machinery. We also do not know whether the restriction-modification activities themselves are involved in such movement.

Type I Group 2 and Group 3 S are present at multiple chromosomal loci (Fig. 3EFIJ) with linked R and M genes in only one of the loci. It is likely that these RM genes can interact with both S subunits and recognize two specific sequences. Similar cases have been reported for *Staphylococcus aureus* [31], [32] and *Mycoplasma pneumoniae* [37]. In two loci of *Mycoplasma pulmonis*, two S genes flank the R and M genes in inverted orientation and are prone to inversion at site-specific recombination site within their ORF, resulting in the shuffling of their TRDs [38]. These S genes carry repeat sequences equivalent to y sequences in Group 2 Type I S genes and in the Type IIG S genes but lack x sequence equivalents. The site-specific recombination likely takes place between a specific sequence in the left y sequence of a gene and the same sequence in another gene or between those in the right y sequences. Organization of the three S loci found in *Mycoplasma agalactiae* (see above) can be represented as S1-M-hypothetical gene-S2-*int*-R-S3-M, where *int* indicates an integrase gene homolog. We do not know whether this integrase homolog is involved in recombination between the S loci as found in *Mycoplasma pulmonis* [39]. In *Lactococcus lactis*, two copies of the S genes on different plasmids, interact through homologous recombination and create two chimeric S genes for one RM system, resulting in shuffled recognition sequences [40].

Restriction-modification systems are mobile [13], [15]. Some of these are present on discrete mobile elements such as plasmids, prophages, and transposons [14], [41], [42], whereas others are themselves similar to transposons in terms of organization [13]. In addition to the mobility at the levels of genes, RM systems, and mobile elements, mobility at the domain level, found here, would contribute to diversification related to biology and epigenetics in a unique manner. Specifically, domain movements and repeat-mediated inter-locus movements would allow changes in sequence specificity in an organized manner, not disturbing genome organization. Many previous experiments (Introduction and the first part in Results) have established that TRD1 recognizes the 5′ half of the recognition sequence and TRD2 the 3′ half. We predict that DOMO generates diversity in sequence recognition in this way, which in turn increases the repertoire in the epigenetic status of a genome and allows fine-tuned defense against various DNAs. These differences may enable adaptation to different environments. Future experiments would test this prediction. Unfortunately, at present, there is no general method for determining recognition sequence of Type I or Type IIG RM systems.

Some examples of domain diversification of bacterial genes were observed in the antigenic variation of pathogenic bacteria by the mechanism of gene conversion [43]. For example, pilin variation in *Neisseria gonorrhoeae* is achieved by gene conversion in the central region of the *pilE* locus with silent *pilS* cassettes, which have hypervariable regions, as the donor [44]. Such diversity enables bacterial cells to evade host immune systems. Gene conversion occurs between genes at different loci, whereas the domain movement can occur within a protein gene of the same locus. At present, we do not know whether the DOMO process is similar to some forms of antigenic variation in terms of the molecular mechanisms involved.

Protein diversification by domain movement through repeat-mediated recombination is similar to that by exon shuffling through recombination at intron DNAs or by alternative RNA splicing [45]. Both processes lead to the deletion or duplication of a domain.

## Materials and Methods

### Genome Sequences

Sequence data for the circularized chromosomes of *H. pylori* were obtained from NCBI Genome as listed in Table S1. Only the upper 10 genomes were used for Group 2 and Group 3 Type I S genes.

### Comparison of RM systems

RM systems were detected by homology search with RM genes registered in REBASE [24] and by search against PFAM [46] and a self-built HMM database composed of restriction endonuclease families [47] using HMMER[48]. Genomic context analyses were carried out using hmmpfam from the HMMER package [48]. RM systems were manually clustered using CGAT [49]. Nucleotide or amino acid sequences were aligned by MAFFT [50] and MUSCLE [51]. Phylogenetic trees were constructed through Bayesian estimation of phylogeny by MrBayes 3.1.2 [52] using the General Time Reversible model incorporating invariant sites and a gamma distribution (GTR+I+G). Two simultaneous analyses, each with four Markov chains, were run for 1,000,000 generations with a sampling in every 100 generations. Trees generated before the stabilization of the likelihood scores were discarded (burn in = 2501), and the remaining trees were used to construct a consensus tree. Nodal support was assessed by posterior probability values.

### Search for domain movement in S genes in species other than *H. pylori*

From the collection of complete genome sequences in REBASE Genomes (http://tools.neb.com/~vincze/genomes/) (as of Feb 3rd, 2011), we chose all the species with multiple genome entries and listed all of their S genes. Dotplot was drawn for S genes of each species by polydot in EMBOSS package [53]. Domain movement was detected by checking dotplots with careful manual curation.

## Supporting Information

Figure S1 **Nucleotide sequence alignment of Group 1 S (See** **Fig. 2** **).**
(PDF)

Figure S2 **Nucleotide sequence alignment of Group 2 S (See** **Fig. 3** **).**
(PDF)

Figure S3 **Nucleotide sequence alignment of Group 3 S (See** **Fig. 3** **).**
(PDF)

Figure S4 **Nucleotide sequence alignment of the S subunit of Type IIG systems (See** **Fig. 5** **).**
(PDF)

Table S1 ***H. pylori* complete genomes analyzed.**
(XLS)
