# Supplementary material for: Domain Movement within a Gene: A Novel Evolutionary Mechanism for Protein Diversification
Source: PLoS One. 2011 Apr 14;6(4):e18819. doi: 10.1371/journal.pone.0018819 (PMC3077401; doi:10.1371/journal.pone.0018819)
Supplement: Figure S1 — Nucleotide sequence alignment of Group 1 S (See Fig. 2 ). (PDF) [file pone.0018819.s001.pdf]

TRD1

```
HP0462       1    ------------------TTGAGTGAGTGGCAAACATTTTGTTTAAAAGATTTAGGAAAAATAGTCGGTG
jhp0414      1    ------------------TTGAGTGAGTGGCAAACATTTTGTTTAAAAGATTTAGGGAAAATAGTCGGCG
HPAG1_0437   1    ATGACTAAACTT-TTAGATTTAAAAAATAATTCAGTAATT--ACTATCAATCAAAACAACTACACTAAAG
HPG27_419    1    TTGAGTGAGCATGTCAAATTAAGTGAAGTATGCGAAATTCTAAATAGCAATGTAGATAAAAAAATAAAAG
HPP12_0434   1    ATGACTAAACTT-TTAGATTTAAAAAATCATTCAGTAATT--GCTATCAATCAAAACAACTATACTAAAG
HPSH_02265   1    ------------------TTGAGTAAAACTCTACAAGACT---ACGCAACTTTAATCAATGATACCATA-
HPF16_0441   1    ATGACTAAACTT-TTAGATTTAAAAAATAATTCAGTGATT--GCTATCAATCAAAACAACTACACTAAAG
HPF30_0860   1    ------------------TTGAGTAAAACTCTACAAGACT---ACGCAACTTTAATCAATGATACCATA-
HPF32_0871   1    ------------------TTGAGTAAAACTCTACAAGATT---ACGCAACTTTAATCAATGATACCATA-
HPF57_0489   1    ------------------TTGAGTGAGTGGCAAACATTTTGTTTAAAAGATTTAGGAAAAATAGTCGGTG
KHP_0426     1    ------------------TTGAGTGAGTGGCAAACATTTTGTTTAAAAGATTTAGGAAAAATAGTCGGTG
HPKB_0443    1    ------------------TTGAGTAAAACTCTACAAGACT---ACGCAACTTTAATCAATGATACCATA-
hp908_0454   1    ----------------------------------------------------------------------
HELPY_0444   1    ATGACTAAACTT-TTAGATTTAAAAAATAATTCAATAATT--GCTATCAACCAAAACAACTACACTAAAG
HPB8_1123    1    ATGACTAAACTT-TTAGATTTAAAAAATCATTCAGTAATT--GCTATCAATCAAAACAACTACACTAAAG
HPCU_02530   1    ------------------TTGAGTGAATGGCAAACATTTTGTTTAAAAGATTTAGGAAAAATAGTCGGTG
HPPC_02215   1    ------------------TTGAGTGAGTGGCAAACATTTTGTTTAAAAGATTTAGGGAAAATAGTCGGTG
HPSJM_02310  1    ------------------TTGAGTGAGTGGCAAACATTTTGTTTAAAAGATTTAGGGAAAATCGTAACAG
HPSAT_02200  1    TTGAGTGACCATGTCAAATTAAGTGAAGTATGCGAAATTTTAAATAGCAATGTAGATAAAAAAACAAAAG
HPV225_0460  1    ------------------TTGAGTGAGTGGCAAACATTTTGTTTAAAAGATTTAGGAAAAATCGTAACAG

HP0462       53   GCGCTACCCCACCTACCAATAACCCCAAAAATTATGGAAACAAAATTAGTTGGATTACCCCTAAAGATTT
jhp0414      53   GCGCTACCCCACCTACCAATAACCCCAAAAATTATGGCAATAAAATTGCTTGGATTACCCCTAAAGATTT
HPAG1_0437   68   AAGACAATTACAAA------AAAGTTTATTATTTAGATA-CTGATA-ATATCACAAACAATAAAATAAAT
HPG27_419    71   AAAACGAACAAAAAGTTAAACTCTGTAATTT---TACAGATGTATATAATAATTGGGCGA-TAACAAAAT
HPP12_0434   68   AAGACAATTACAAA------AAAGTTTATTATTTAGATA-CTGATA-ACATCACAAACAATAGAATAAAT
HPSH_02265   48   ----CAATCAAATG-------AAATCAATCACT---ATA-TCACCACAGCTAACATGTGCCAAAATTTAG
HPF16_0441   68   AAGACAATTACAAA------AAAGTTTGTTATTTAGATA-CTGACA-ACATTACAAATAATAGAATAAAT
HPF30_0860   48   ----CAATCAAATG-------AAATCAATCACT---ATA-TAACTACGGATAATATGTGCCAAAATTTAG
HPF32_0871   48   ----CAATCAAATG-------AAATCAATCACT---ATA-TCACTACGGATAACATGTGCCAAAATTTAG
HPF57_0489   53   GCGCTACCCCATCTACCAATAACCCCAAAAACTATGGCAATAAAATTGCTTGGATTACCCCTAAAGATTT
KHP_0426     53   GCGCTACCCCATCTACCAATAACCCCAAAAACTATGGCAATAAAATTGCTTGGATTACCCCTAAAGATTT
HPKB_0443    48   ----CAATCAAATG-------AAATCAATCACT---ATA-TAACTACGGATAACATGTGCCAAAATTTAG
hp908_0454   1    ----------------------------------------------------------------------
HELPY_0444   68   AAGACAATTACAAA------AAAGTTTGTTATTTAGATA-CTGACA-ACATTACAAATAATAGAATAAAT
HPB8_1123    68   AATACAATTACAAA---AAAGTTTGTTATTTAGATACTGACAACATTACAAATAATAAAA-TAAATGCTT
HPCU_02530   53   GCGCTACCCCATCTACCAATAACCCCAAAAACTATGGCAATAAAATTGCTTGGATTACCCCTAAAGATTT
HPPC_02215   53   GTGCTACCCCATCTACCAATAACCCCAAAAACTATGGCAATAAAATTGCTTGGATCACCCCTAAAGATTT
HPSJM_02310  53   GAAAAACCCCTAAAACTTCTAATTTAGATTTTTTTAATGGCAAATACATGTTTATTACGCCAAATGATTT
HPSAT_02200  71   AAAACGAACAAAAAGT---TAAACTCTGTAATTT---TA-TAGATGTATATAATAATTGGGCGATAACAA
HPV225_0460  53   GAAAAACCCCTAAAACTTCTAATTTAGATTTTTTTAATGGCAAATACATGTTTATTACGCCAAATGATTT

HP0462       123  ATCCACTTTACAAG-GGCGTTACATTAAAAAAGGCAGCCGCAGCATTTCACGCTTAGGATTTAAGT--CA
jhp0414      123  ATCCACTTTACAAGGGCGCTA-CATTAAAAAAGGCAGCCGCAGCATTTCACGATTAGGGTTTAAATCATG
HPAG1_0437   130  GCTTTTCTAAAAATTGATTTAACAAAAGAAAAATTGCCTTCAAGAGCTAAACGAAAATGCTCCATTAATA
HPG27_419    137  ATACAAGTAAAAAA-TTTATGAAAGCAA--CCGCCACACAAAACGA--AATTAATAAATTTTCATTAAAA
HPP12_0434   130  GCTTTTCTAAAAATTGATTTGACAAAAGAAAAACTGCCTTCAAGAGCTAAACGAAAATGCTCCATTAATA
HPSH_02265   104  GCGGTATTGATAC---ATTTA--AAAACATCAATATCCCACAAGGAAAGGTC-AGATCATTTCAAAAAGA
HPF16_0441   130  GCTTTTCTAAAAATTGATTTAACAAAAGAAAAATTGCCTTCAAGAGCTAAACGAAAATGCTCTCTTAATA
HPF30_0860   104  GCGGTATTGATAC---ATTTA--AAAATATCAATATCCCACAAGGAAAGGTC-AGATCATTTCAAAAAGA
HPF32_0871   104  GAGGTATTGATAC---ATTTA--AAAATATCAATATCCCACAAGGAAAGGTC-AGATCATTTCAAAAAGA
HPF57_0489   123  ATCCACTTTACAAGGGCGTTA-CATTAAAAAAGGCAGCCGCAGCATTTCACGCTTAGGATTTAAGTCATG
KHP_0426     123  ATCCACTTTACAAG-GGTGCTACATTAAAAAAGGCAACCGCAGCATTTCACGATTAGGATTTAAGT--CA
HPKB_0443    104  GCGGTATTGATAC---ACTTA--AAAATATCAATATCCCACAAGAAAAGGTC-AGATCATTTCAAAAAGA
hp908_0454   1    ----------------------------------------------------------------------
HELPY_0444   130  GCTTTTCTAAAAATTGATTTAACAAAAGAAAAATTACCCTCAAGAGCTAAACGAAAATGCTCCATTAATA
HPB8_1123    134  TTCTAAAAATTGAT-TTAACAAAAGAAAAACTGCCCTCAAGAGCTA--AACGAA-AATGCTCCATTAATA
HPCU_02530   123  ATCCACTTTACAAGGGCGTTA-CATTAAAAAAGGCAGCCGCAGCATTTCACGCTTAGGATTTAAGTCATG
HPPC_02215   123  ATCCACTTTACAAG-GGCGTTACATTAAAAAAGGCAGCCGCAGCATTTCACGCTTAGGATTTAAATCATG
HPSJM_02310  123  GCATGGCACATAT----CGTATTATTAAAA----CACCAAGAACGCTTAGTGATAGTGGTTTAAAAAGCA
HPSAT_02200  134  AATATACAAGTAAAAAATTTATGACAGCAACCGCTACA--CAAAATGAAATCAATAAATTTTCATTAAAA
HPV225_0460  123  GCATGGCACATA----TCGTGTTATTAAAA----CACCAAGAACGCTTAGTGATAGTGGTTTAAAGAGCA
```

```
HP0462       190  TGCTCTTGTGTGTTGCTCCCAAAACATGCCATTTTATTTTCTTCAAGAGCTCCCATAGGT-TATGTGGCA
jhp0414      192  CTC--TTGTGTGTTGCTCCCAAAGCATGCCATTT---TATTTTCTTCAAGAGCTCCCATA-GGTTATGTG
HPAG1_0437   200  GCA--TTAT----TTATTCTAGTGTAAGACCTAACCAACGCCACTTTGGG-ATTATCAAA-GAAATTCCC
HPG27_419    202  AAAGGTTATGTTGCTATTACAAAGGATAGCGAAACAAAAGATGATATAGGCATTTCTACT-TATATTGCA
HPP12_0434   200  GCA--TTAT----TTATTCTAGTGTAAGACCTAACCAACGCCACTTTGGG-ATTATCAAA-GAAATTCCC
HPSH_02265   168  TGA-----------TGTTTTGTTGTCAAATATAG--ACCCTTGGCATAGACAAGTCTATA-TGGCAAAAC
HPF16_0441   200  GCA--TTAT----TTATTCTAGTGTAAGACCTAACCAACGCCACTTTGGG-ATTATCAAA-GAAATTCCA
HPF30_0860   168  TGA-----------TGTTTTGTTGTCAAATATAA--GACTTTATTTTAGAAAAGTTTATA-GAGCCAAAC
HPF32_0871   168  TGA-----------TGTTTTATTGTCAAATATAA--GACTTTATTTTAGAAAAGTTTATA-GAGCCAAAC
HPF57_0489   192  TTC--TTGTGTGTTACTCCCAAAACATGCCATTT---TATTTTCTTCAAGAGCTCCCATA-GGTTATGTG
KHP_0426     190  TGCTCTTGTGTGTTACTCCCAAAACATGCCATTTTATTTTCTTCAAGAGCTCCCATAGGT-TATGTGGCT
HPKB_0443    168  TGA-----------TGTTTTGTTATCAAATATAA--GACTTTATTTTAGAAAAGTTTATA-GAGCCAAAC
hp908_0454   1    ----------------------------------------------------------------------
HELPY_0444   200  GCA--TTAT----TTATTCTAGTGTAAGACCTAACCAACGCCACTTTGGG-ATTATCAAA-GAAATTCCA
HPB8_1123    200  GCA--TTAT----TTATTCTAGCGTAAGACCCAACCAACGCCACTTTGGG-ATTATCAAA-GAAATTCCC
HPCU_02530   192  CTC--TTGTGTGTTACTCCCAAAACATGCCATTT---TATTTTCTTCAAGAGCTCCCATA-GGTTATGTA
HPPC_02215   192  CTCTTGTGTGT--TGCTCCCAAAACATGCCATTTT-ATTTTCTTCAAGAGCTCCCATAGG-TTATGTGGC
HPSJM_02310  185  TACAAAACAA-------CACAATAGATAACACAA--GCATTCTTGTAGGGTGT--ATAGG-TGATGTGGG
HPSAT_02200  202  AAAGGTTATGTTGCTATTACAAAGGATAGCGAAACAAAAAATGATATAGGCATTTCCACT-TATATTGCG
HPV225_0460  185  TACAAAACAACACAATAGATAATACAAGC-ATTCTTGTAGGGTGTATAGGTGATGTGGGTATGGTTCGCA

HP0462       259  ATTGCTGAAAAAAGGCTATGCACCAATCAAGGTTTTAAAAGCATTATCCCTAACAAAA---AAATTT---
jhp0414      256  GCAATTGCTGAAAAAAGGCTATGCACCAATCAAGGTTTTAAAAGTATTATCCCTAACAAAAAAATTTA--
HPAG1_0437   262  AAAAATTTTT-TAGTTTCTACCGCTTTTATCGTAATAGATATTATAGACTTAAAAAAATTAGACCCTAAT
HPG27_419    271  GATAATTTTGATAATGTGTTACTAGGTTATCATTGCACTTTGCTTAAACCTAATCAAA--AGGTTTTAA-
HPP12_0434   262  AAAAATTTTT-TAGTTTCTACCGCTTTTATCGTAATAGATGTTATAGATTTAAAAAAATTAGACCCTAAT
HPSH_02265   224  AAAAAGGCGG-----TTGCAGTTCTGATGTCCTAGTGTTTAGGGCTAAACACATTGA--TAGCGCCACAC
HPF16_0441   262  AAAAATTTTT-TAGTTTCTACCGCTTTTATCGTGATAGACATTATAGATTTAGAAAAATTAGACCCTAAT
HPF30_0860   224  AAAAAGGCGG-----TTGCAGTTCTGATGTCCTAGTGTTTAGGGCTAAACACATTGA--TAGCGCTACAC
HPF32_0871   224  AAAAAGGCGG-----TTGCAGTTCTGATGTCCTAGTGTTTAGGGCTAAACGCATTGA--TAGTGCTACAC
HPF57_0489   256  GCTATTGCTAAAAAAAGGCTATGCACCAATCAAGGTTTTAAAAGCATTATCCCTAACAAAAAAATTTA--
KHP_0426     259  ATTGCCAAAAAAAGGCTATGCACCAATCAAGGTTTTAAAAGCATTATCCCCAACAAAA---AAATTT---
HPKB_0443    224  AAAAAGGCGG-----TTGCAGTTCTGATGTCCTAGTGTTTAGGGCTAAACACATTGA--TAGTGCCACAC
hp908_0454   1    --------------------------------------ATGGGGCTAGTCCCTAATAAAAAAATTTA---
HELPY_0444   262  AAAAATTTTT-TAGTTTCTACTGCTTTTATCGTAATAGATATTATAGATTTAAAAAAATTAGACCCTAAT
HPB8_1123    262  AAAAATTTTT-TAGTTTCTACCGCTTTTATCGTAATAGATGTTATAGACTTAGAAAAATTAGACCCTAAT
HPCU_02530   256  GCTATTGCTAAAAAAAGGCTATGCACCAATCAAGGTTTTAAAAGCATTATCCCCAACAAAAAAATTTA--
HPPC_02215   258  TATTGCTGAAAAAAG----GCTATGCACCAATCAAGGTTTTAAAAGCATT--ATCCCT-AACAAAAAAAT
HPSJM_02310  243  TATGGTTCGCATGTGTTTTGATAAATGCGCAACAAATCAGCAAATAAATTCTATTACA-GACATTAAGGA
HPSAT_02200  271  GATAATTTTGATAATGTGTTACTAGGTTATCATTGTACTTTGCTTAAACCTAATCAAA--AGGTTTTAA-
HPV225_0460  254  TGTGTTTTGATAAATGCGCA-ACAAATCAACAAATAAATTCTATTACAGACATTAAAG---ATTTTTGTA

HP0462       323  ATTTTGAAT--TTTTATACTACTTACTAAAGTAT--CAT--------AAAAACAATTTTATAAACATGGG
jhp0414      323  -TTTTGAAT--TTTTATATTACTTATTAAAATACTATAAGGATAACATTTCCAACATAGGGGGCGGAACT
HPAG1_0437   331  TATTTGTAT--TACTATATTACGCAAGATAAAAT--CACTCATTACTTGCAACGCATTGCAGAATGTGGG
HPG27_419    337  -ATGGAAAA--TTTTTAAATGCTTATTTAAATTC--TTTTTATGGCAGAAAATATTTTTCTAATTGTGCA
HPP12_0434   331  TATTTGTAT--TATTATATTACGCAAGATAAAAT--TACTCATTACTTGCAACGCATTGCAGAATGTGGG
HPSH_02265   287  TTTTTGCGA--TTTTAT-CTAGTCAATCTTTTAT--CAATTATTTATGTTTAGGAAGTGTCGGTTCAAAG
HPF16_0441   331  TATTTGTAT--TATTATATTACGCAAGATAAAAT--CACTCATTACTTGCAACGCATTGCAGAATGTGGG
HPF30_0860   287  TTTTTGCGA--TTTTAT-CTAGTCAAATTTTTAC--TGATTATGCGTGTTCAGGCAGTCAAGGTTCTAAA
HPF32_0871   287  TTTTTGCGA--TTTTAT-CTAGTCAAATTTTTAC--TGATTATGCGTGTTCAGGCAGTCAAGGTTCTAAA
HPF57_0489   323  -TTTTGAAT--TTTTATACTACTTACTCAAATACCATAAGGATAACATCTCTAACATGGGAGTTGGCACT
KHP_0426     323  ATTTTGAAT--TTTTATACTACTTACTCAAATAC--CAT--------AAGGATAATATCTCTAACATAGG
HPKB_0443    287  TTTTTGCGA--TTTTAT-CTAGTCAAATTTTTAC--TGATTATGCGTGTTCAGGCAGTCAAGGTTCTAAA
hp908_0454   29   -TTTTGAAT--TTTTATACTACTTACTCAAATACCATAAGGATAACATCTCTAACATGGGAGTTGGCACT
HELPY_0444   331  TATTTGTAT--TATTATATTACGCAAGATAAAAT--CACTCATTACTTGCAACGCATTGCAGAATGTGGG
HPB8_1123    331  TATTTGTAT--TATTACATTACGCAAGATGAAAT--CATTCATTACTTGCAACGCATCGCAGAATGTGGG
HPCU_02530   323  -TTTTGAAT--TTTTATACTACTTACTCAAATACCATAAGGATAATATCTCTAACATAGGGGGCGGAACT
HPPC_02215   321  TTATTTTGAATTTTTATACTACTTACTCAAATACCATAAGGATAACATCTCTAACATAGGAGGAGGAACT
HPSJM_02310  312  TTTTTGTAA---TCCATACTACTTATATTATTATCTATCTAATAAAAAAGAGCTTTTTAAAAATATAGCT
HPSAT_02200  337  -ATGGAAAA--TTTTTAAATGCTTATTTAAGTTC--TTTTTATGGTAGAAAATATTTTTCTAATTGTGCA
HPV225_0460  320  ATCCATACT--ACTTATACTACTATCTATCTAAT--AAA--------AAAGAACTTTTTAAAAATATAGC
```

```
HP0462        381  TGAAGGAACTACTATTAAAGGAATTTATAATATTGCTTTAGGTCTGTTTAAAGTTAAGATACCCCCTACT
jhp0414       391  ACTTTTAAAGAAGTTTCAGGGGCTA---CTTTAGGTCTATTCCAAGTTAAGAT--ACCCCCCACTTATTA
HPAG1_0437    397  ACTTCTAGCTACCCCTCTATCACGC---CCTTAGATTTTCTTAATATTAAAATCAAAC-TTTACCCACTA
HPG27_419     403  AGTGGAAGCGGTCAAAGATACACACTAACTATTGATACTATTAAGGATTTAAATATACCTTTAATTAACA
HPP12_0434    397  ACTTCTAGCTACCCCTCTATCACGC---CCTTAGATTTTCTTAATATTAAAGTCAAAC-TTTACCCACTA
HPSH_02265    352  AGAAAAAGGGG-AGATAAAACACAC--ATGATGGATTTCAAAATCCCTACAATCAATTTTACAATCGCTA
HPF16_0441    397  ACTTCCAGCTACCCCTCTATCACGC---CCTTAGATTTTCTTAATATTAAAATCAAAC-TTTACCTACTA
HPF30_0860    352  ATGCCAAGAGG-CAATAAAACACAC--ATGATGGATTTCAAAATCCCTACAATCAATTTTACAATCGCTA
HPF32_0871    352  ATGCCAAGAGG-CAATAAAACACAC--ATGATGGATTTCAAAATCCCTACAATCAATTTTACAATCGCTA
HPF57_0489    391  ACTTTTAAGGATATTTCAAAGCCAG---CTTTAGGTCTGTTTAAAGTTAAGAT--ACCCCCTACTTATTA
KHP_0426      381  GGGCGGAACTACTTTTAAAGAAGTTTCAGGGGCTACTTTAGGTCTGTTTAAAGTTAAGATCCCCCCTACT
HPKB_0443     352  ATGCCAAGAGG-CAATAAAACACAC--ATGATGGATTTTAAAATCCCTACAATCAATTTTACAATCGCTA
hp908_0454    97   ACTTTTAAGGGTATTTCAAAGCCAG---CGTTAGGACTATTCCAAGTTAAGAT--ACCCCCCACTTATTA
HELPY_0444    397  ACTTCTAGCTACCCATCTATCACGC---CCTTAGATTTTCTCAATATTAAAATCAAAC-TTTACCCACTA
HPB8_1123     397  ACTTCTAGCTACCC---CTCTATTACACCCCTAGATTTTCTTAAT-ATTAAAATCAAACTTTACCCA--C
HPCU_02530    391  ACTTTTAAAGAAGTTTCAGGGGCTA---CTTTAGGTCTGTTTAAAGTTAAGAT--ACCCCCTACTTATTA
HPPC_02215    391  ACTTTTAAAGAAGTTTCAGGGGCTA---CTTTAAGTCTGTTTGAAGT--TAAGATACCCCCTACTTATTA
HPSJM_02310   379  CTCTCTACAGTTGTGCCAATTATTC---CAAAAACAATTTTTCAAGAAATAGAAGTTTTATTACCAAATA
HPSAT_02200   403  AGTGGAAGCGGTCAAAGATACACACTAACTATTGATATTATTAAGGATTTAACCATACCTTTAATTAATA
HPV225_0460   378  TCTCTCTACAGTTGTGCCAATTATTCCAAAAACAACTTTTCAAGAGATAGAAGTCTTGTTGCCAAATATA
                                                                          conserved region
HP0462        451  TATTACGAACAACAAAAAATAGCCCGCACGCTTTCTATCCTAGACCAAAAAATAGAGAACAACCACAAAA
jhp0414       456  C-----GAACAACAAAAAATCGCCCACACACTTTCTATTTTAGATCAAAAAATAGAGAACAACCATAAAA
HPAG1_0437    462  --G-AAACACAACAAAAAATCGCCCGCACGCTTTCTGTCTTAGATCAAAAAATAGAGAACAACCACAAAA
HPG27_419     473  TAG-AAACACAACAAAAAATCGCCCGCACGCTTTCTGTTTTAGATCAAAAAATAGAAAACAACCACAAAA
HPP12_0434    462  --G-AAACACAACAAAAAATCGCCCGCACGCTTTCTATTTTAGATCAAAAAATAGAGAACAACCATAAAA
HPSH_02265    418  ----------------AAATTTTTAATAG------CATTCAAAAT---AAGATTGAAAACAACCACAAAA
HPF16_0441    462  --G-AAACACAACAAAAAATCGCCCGCACGCTTTCTATCCTAGATCAAAAAATAGAGAACAACCACAAAA
HPF30_0860    418  ----------------AAATTTTTAATAG------CATTCAAAAT---AAGATTGAAAACAACCACAAAA
HPF32_0871    418  ----------------AAATTTTTAATAG------CATTCAAAAT---AAGATTGAGAACAACCACAAAA
HPF57_0489    456  C-----GAACAACAAAAAATCGCCCGCACGCTTTCTATCCTAGATCAAAAAATTGAAAACAACCACAAAA
KHP_0426      451  TATTACGAACAACAAAAAATAGCCCGCACGCTTTCTATCCTAGATCAAAAAATAGAGAACAACCACAAAA
HPKB_0443     418  ----------------AAATTTTTAATAG------CATTCAAAAT---AAGATTGAAAACAACCACAAAA
hp908_0454    162  C-----GAACAACAAAAAATCGCCCGCACGCTTTCTGTCTTAGATCAAAAAATAGAAAACAACCATAAAA
HELPY_0444    462  --G-AAACACAGCAAAAAATCGCCCGCACGCTTTCTGTTTTAGATCAAAAAATAGAGAACAACCACAAAA
HPB8_1123     461  TAG-AAACACAACAAAAAATCGCCCGCACGCTTTCTGTCTTAGATCAAAAAATAGAGAACAACCATAAAA
HPCU_02530    456  C-----GAACAACAAAAAATCGCCCGCACGCTTTCTGTCCTAGATCAAAAAATAGAGAACAACCACAAAA
HPPC_02215    456  CGA-A----CAACAAAAAATCGCCCGCACGCTTTCTGTCTTAGATCAAAAAATAGAAAACAACCACAAAA
HPSJM_02310   446  TAG-AAACACAACAAAAAATCGCCCGCACGCTTTCTGTTTTAGATCAAAAAATAGAGAACAACCACAAAA
HPSAT_02200   473  TAG-AAACACAACAAAAAATCGTCCGCACGCTTTCTATCCTAGATCAAAAAATAGAGAACAACCACAAAA
HPV225_0460   448  GAA---ACACAACAAAAAATCGCCCGCACGCTTTCTATCCTAGATCAAAAAATAGAGAACAACCACAAAA
                                     TRD2
HP0462        521  TCAACGAGCTTTTACACACGCTCGCTTATAAAATCTATGAATATTA---------TTTCAAATACAAACC
jhp0414       521  TCAATGAGCTTTTACACAAAATCTTAGAGCTTCTTTATGAGCAATACTTCGTCCGTTTTGATTTTTTAGA
HPAG1_0437    530  TCAATGAGCTTTTACACAAGATCCTAGAACTTCTTTATGAGCAATACTTCGTTCGTTTTGATTTTTTAGA
HPG27_419     542  TCAACGAGCTTTTACACACCCTCGCTTATAAAATCTATGAATATTA---------TTTCAAATACAAACC
HPP12_0434    530  TCAACGAGCTTTTACACAAAATCCTAGAACTTCTTTATGAGCAATACTTCGTTCGTTTTGATTTTTTAGA
HPSH_02265    464  TCAATGAGATTTTACACAAAATCCTAGAACTTCTTTATGAGCAATACTTCGTTCGTTTTGATTTTTTAGA
HPF16_0441    530  TCAATGAACTTTTACACAAAATCCTAGAACTTCTTTATGAGCAATACTTCGTTCGTTTTGATTTTTTAGA
HPF30_0860    464  TCAATGAGCTTTTACACAAAATCCTAGAACTTCTTTATGAGCAATACTTCGTTCGTTTTGATTTTTTAGA
HPF32_0871    464  TCAATGAGCTTTTACACAAAATCCTAGAGCTTCTTTATGAGCAATACTTCGTTCGTTTTGATTTTTTAGA
HPF57_0489    521  TCAATGAACTTTTACACAAAATCCTAGAACTTCTTTATGAGCAATACTTCGTTCGTTTTGATTTTTTAGA
KHP_0426      521  TCAATGAGCTTTTACACACGCTCGCTTATAAAATCTATGAATATTA---------TTTCAAATACAAACC
HPKB_0443     464  TCAATGAGCTTTTACACAAAATCCTAGAACTTCTTTATGAGCAATACTTCGTTCGTTTTGATTTTTTAGA
hp908_0454    227  TCAATGAGCTTTTACACAAAATCCTAGAGCTTCTTTATGAGCAATACTTCGTCCGTTTTGATTTTTCAGA
HELPY_0444    530  TCAACGAGCTTTTACACAAAATCCTAGAACTTCTTTATGAGCAATACTTCGTTCGTTTTGATTTTTTAGA
HPB8_1123     530  TCAACGAGCTTTTACACACGCTCGCTTATAAAATCTATGAATATTA---------TTTCAAATACAAACC
HPCU_02530    521  TCAATGAGCTTTTACACAAAATCCTAGAACTTCTTTATGAGCAATACTTCGTTCGTTTTGATTTTTTAGA
HPPC_02215    521  TCAATGAGCTTTTACACAAAATCCTAGAACTTCTTTATGAGCAATACTTCGTTCGTTTTGATTTTTTAGA
HPSJM_02310   515  TCAACGAGCTTTTACACAAAATCCTAGAACTTCTTTATGAGCAATACTTCGTTCGTTTTGATTTTTTAGA
HPSAT_02200   542  TCAATGAGCTTTTACACAAAATCCTAGAACTTCTTTATGAGCAATACTTCGTTCGTTTTGATTTTTTAGA
HPV225_0460   515  TCAATGAGCTTTTACACACACTCGCTTATAAAATCTATGAATATTA---------TTTCAAATACAAACC
```

```
HP0462        582  TAAAAATGCAAAGCTAGAACAAATTATTATTGAAAATCCTAAATCT-----AATATTATGGTTAAAAACG
jhp0414       591  TGAAAACAACAAACCCTATCAAACTAGCGGCGGGAAAATGAAATTTTCTAAAGAATTAAACCGCCTTATC
HPAG1_0437    600  TGAAAACAACAAACCCTATCAAACTAGCGGCGGGAAAATGAAATTCTCTAAAGAATTAAACCGCCTTATC
HPG27_419     603  TAAAAATGCAAAGCTAGAACAAATTATTATTGAAAATCCTAAATCT-----AGTATTATGGTTAAAAACG
HPP12_0434    600  TGAAAACAACAAACCCTATCAAACTAGTGGCGGGAAAATGAAATTCTCTAAAGAATTAAACCGCCTTATC
HPSH_02265    534  TGAAAACAACAAACCCTATCAAACTAGCGGCGGAAAAATGAAATTTTCTAAAGAATTAAACCGCCTTATC
HPF16_0441    600  TGAAAACAACAAGCCCTATCAAACCAGCGGCGGAAAAATGAAATTTTCTAAAGAATTAAACCGCCTTATC
HPF30_0860    534  TGAAAACAACAAACCCTATCAAACTAGCGGCGGAAAAATGAAATTTTCTAAAGAATTAAACCGCCTTATC
HPF32_0871    534  TGAAAACAACAAACCCTATCAAACCAGCGGCGGAAAAATGAAATTTTCTAAAGAATTAAACCGCCTTATC
HPF57_0489    591  TGAAAACAACAAACCCTATCAAACCAGCGGCGGAAAAATGAAATTTTCTAAAGAATTAAACCGCCTTATC
KHP_0426      582  TAAAAATGCAAAGCTAGAACAAATTATTATTGAAAATCCTAAATCT-----AGTATTATGGTTAAAAACG
HPKB_0443     534  TGAAAACAACAAACCCTATCAAACTAGCGGCGGGAAAATGAAATTCTCTAAAGAATTAAACCGCCTTATC
hp908_0454    297  TGAAAACAACAAACCCTATCAAACTAGCGGCGGAAAAATGAAATTTTCTAAAGAATTAAACCGCCTTATC
HELPY_0444    600  TGAAAACAACAAACCCTATCAAACTAGCGGCGGGAAAATGAAATTCTCTAAAGAATTAAACCGCCTTATC
HPB8_1123     591  TAAAAATGCAAAGCTAGAACAAATTATTATTGAAAATCCTAAATCT-----AATATTATGGTTAAAAACG
HPCU_02530    591  TGGAAACAACAAACCCTATCAAACTAGCGGCGGAAAAATGAAATTTTCTAAAGAATTAAACCGCCTTATC
HPPC_02215    591  TGGAAACAACAAACCCTATCAAACTAGCGGCGGAAAAATGAAATTTTCTAAAGAATTAAACCGCCTTATC
HPSJM_02310   585  TGAAAACAACAAACCCTATCAAACTAGCGGCGGGAAAATGAAATTCTCTAAAGAATTAAACCGCCTTATC
HPSAT_02200   612  TGGAAACAACAAACCCTATCAAACTAGCGGCGGAAAAATGAAATTTTCTAAAGAATTAAACCGCCTTATC
HPV225_0460   576  TAAAAATGCAAATCTAGAACAAATTATTATTGAAAATCCTAAATCT-----AGTATTATGGTTAAAAACG

HP0462        647  CCCAAAAAACCCAAGATAAATACCCT--------------------------------------------
jhp0414       661  CCTAACGATTTTAAAGTCAAAACGCTAGGGGAACTAATAACT----TGGATTTCCGGAAGTCAGC-----
HPAG1_0437    670  CCCAACGATTTTGAAGTCAAAACGCTAGGGGA--TAATCCCCTATGTAACACGATCAAAACCGGAGTAAC
HPG27_419     668  CCCAAAAAACCCAAGATAAATACCCC--------------------------------------------
HPP12_0434    670  CCCAACGATTTTGAAGTTAAAACACTAGGGG----AAT--------TAACCCAATTAAAAGTAGG-----
HPSH_02265    604  CCCAACGATTTTGAAGTTAAAACGCTAGGGGAATTAACCCAATTAAAAGTAGGA----AAC-----AAAA
HPF16_0441    670  CCCAACGATTTTGAAGTTAAAACGCTAGGGG----AATTAATAACTTGGATTTCCGGAAGTCAGC-----
HPF30_0860    604  CCCAACGATTTTGAAGTTAAAACGCTAGGGGA--TAATCCCCTATGTAACACAATCAAAACCGGAGTAAC
HPF32_0871    604  CCTAGTGGGTGGAGCGTAAGATTTTTAAATCA--TAAAATTGTAT-CAACTTATC---AACC----TAAA
HPF57_0489    661  CCCAACGATTTTGAAGTTAAAACGCTAGGGG----AAT--------TAACCCAATTAAAAGTAGG-----
KHP_0426      647  CCCAAAAAACCCAAGATAAATACCTC--------------------------------------------
HPKB_0443     604  CCCAACGATTTTGAAGTTAAAACGCTAGGGGAATTAATAACTTGGATTTCCGGAAGTCAGCCACCCAAAA
hp908_0454    367  CCTAATGGGTGGAGCGTAAGATTTTTAAATCATAAAATTGTATCAACTTATCAACCTAAAACCAT-----
HELPY_0444    670  CCCAACGATTTTGAAGTTAAAACGCTAGGGG----AACTAGT----GGATATTTTCAGCGGTTAT-----
HPB8_1123     656  CCCAAAAAACCCAAGATAAATACCCC--------------------------------------------
HPCU_02530    661  CCCAACGATTTTGAAGTTAAAACGCTAGGGG----AAT--------TAACCCAATTAAAAGTAGG-----
HPPC_02215    661  CCTAACGATTTTGAAGTTAAAACGCTAGGGGAACTAGT--------GGATATTTTCAGCGGTTAT-----
HPSJM_02310   655  CCCAACGATTTTGAAGTTAAAACGCTAGGGGAGTTAGT--------GGATATTTTCAGCGGTTAT-----
HPSAT_02200   682  CCCAACGATTTTGAAGTTAAAACGCTAGGGG----AACTAGT----GGATATTTTCAGCGGTTAT-----
HPV225_0460   641  CCCAAAAAACCCAAGATAAATACCCC--------------------------------------------

HP0462        672  ---TTTTTTACAAGCG---------GAGATAATATCCTATCCTACCCTAAAGCGATCATTGATGG-----
jhp0414       721  ------CACCCAAAAGTTGTCACATATACGAGTA-TAAAGAGGGTTACATTCGTTTCATACAAAA---CA
HPAG1_0437    738  CCCCTTTAAACAAAAAGTGTATTATGAAACAAAA-CATATACAAGAAACCCTATCTCTTAATCAAGGACT
HPG27_419     693  ---TTTTTTACAAGTG---------GAGATAATATCTTATCCTATCCTAAAGCAATCATTGATGG-----
HPP12_0434    722  -------AAACAAAAACGCTA--ATCACTCATCT-GATCAAGGAAAATATCCATTTTTTACTTG----CT
HPSH_02265    665  ATGCTAATCACTCAT-----CTAATCAAGGAAAA-TATCCATTTTTTACTTGCTCAAACAATCCTTTA--
HPF16_0441    730  ------CACCCAAAAGTTGTCATATATACGAGCA-TAAAGAGGGTTACATTCGTTTCATACAAAA---CA
HPF30_0860    672  CCCCTTTAAACAAAAAGTGTATTATGAAACAAAA-CATATACAAGAAACCCTATCTCTTAATCAAGGA-C
HPF32_0871    664  ACCATT--AGCAAAA----CCTTACTAAATGATA-GTTATTCTT-ATAGTGTATATGGTGGCGGTGGA-A
HPF57_0489    713  -------AAACAAAAAT---GCTAATCACTCATCTAATCAAGGAAAATATCCATTTTTTACTTGC-----
KHP_0426      672  ---TTTTTTACAAGCG---------GAGATAATATCCTATCTTATCCTCAAGCGATCATTGATGG-----
HPKB_0443     674  GTTGTCATATATACG-----AGCATAAAGAGGGT-TACATTCGTTTCATACAAAACAGAGATTATAGCTC
hp908_0454    431  -------TAGCAAAACC----TTACTAAATGATA--GTTATTCTTATAGTGTATATGGTGGCGGTGGA-A
HELPY_0444    727  TCTTTTCAATCAAATAC------ATACAGCAACAACAAAAATGATTATATGTTAATCACAAATAA----A
HPB8_1123     681  ---TTTTTTACAAGCG---------GAGATAATATCCTATCCTATCCTAAAGCGATCATTGATGG-----
HPCU_02530    713  -------AAACAAAAAT---GCTAATCACTCATCTAATCAAGGAAAATATCCATTTTTTACTTGC-----
HPPC_02215    718  TCTTTTCAATCAAATAC------ATACAGCAACAACAAAAATGATTATATTTTAATCACAAATAA----A
HPSJM_02310   712  TCTTTTCAATCAAATAC------ATACAGCAACAACAAAAATGATTATATTTTAATCACAAATAA----A
HPSAT_02200   739  TCTTTTCAATCAAATAC------ATACAGCAACAACAAAAATGATTATATTTTAATCACAAATAA----A
HPV225_0460   666  ---TTTTTTACAAGCG---------GAGATAATATCCTATTCTATCCTAAAGCGATCATTGATGG-----
```

```
HP0462        725  -------------CAGAAATTGCTTTTTAAACACTGGGGGTAATGCTGGTATTA------AATTTTATGT
jhp0414       782  GAGATTATAGCTCTAATAATTATGTTACATATATT--CCTATATCAAAAAATAA---CAAGATTTGTTAT
HPAG1_0437    807  TAAAGT-TAGCTATAACAAACGCCCTAATAGAGCCAACATGCAACCCACCATATACTCAGTATGGTTTGC
HPG27_419     746  -------------CAGAAATTGCTTTTTAAATACTGGGGGTAATGCTGGTATTA------AATTTTATGT
HPP12_0434    779  CAAACA-ATCCTTTAAAATGCG----AAACATATCAATTTGAAGGAAAACATAT------TATTATTTCT
HPSH_02265    726  --------AGATGCGAAACATATCAATTTGAAGGAA------AACATATTAT--------TATTTCT---
HPF16_0441    791  GAGATTATAGCTCTAATGATTATATTACATATATT--CCTATATCAAAAAATAA---CAAGATTTGTTAT
HPF30_0860    740  TTAAAGTTAGCTATAACAAACGCCCTAATAGAGCCAACATGCAACCCACTATCCATTCAGTATGGTTTGC
HPF32_0871    725  TAATAGGGAGATTTACAGAATACAATCAT-GAGCAATCA--GAATTTATTAT---TTCA-TGTCGTG-GT
HPF57_0489    769  TCAAACAATCCTTTAAGATGCG----AAACATATCAATTTGAAGGAAAACATAT------TATTATTTCT
KHP_0426      725  -------------CAGAAATTGTTTTTTAAACACTGGGGGTAATGCTGATATTA------AATTTTATGT
HPKB_0443     738  TAATGATTATATTACATATATTCCTATATCAAAAAATAACAAGATTTGTTAT-----CAATATGAT----
hp908_0454    488  TAATAGGAAGATTTACAGAATA---TAATCATGAGCAATCAGAATTTATTATTT----CATGTCGTG-GT
HELPY_0444    787  AATGTTCAACATTCATTAATTGATTTAAGTATTACAACTAACCTTTTATTTTTA-CCGAAAAAACTCCCT
HPB8_1123     734  -------------CAGAAATTGCTTTTTAAACACTGGAGGTAATGCTGGTATTA------AATTTTATGT
HPCU_02530    769  TCAAACAATCCTTTAAGATGCG----AAACATATCAATTTGAAGGAAAACATAT------TATTATTTCT
HPPC_02215    778  AATGTTCAACATTCATTAGTTGATTTAAGTATTACAACTAACCTTTTATTTTTA-CCGAAAAAACTCCCT
HPSJM_02310   772  AATGTTCAACATTCATTAGTTGATTTAAGTGTTACAACTAACCTTTTATTTTTA-CCGAAAAAACTCCCT
HPSAT_02200   799  AATGTTCAACATTCATTAGTTGATTTAAGTATTACAACTAACCTTTTATTTTTA-CCGAAAAAACTCCCT
HPV225_0460   719  -------------CAGAAATTGTTTTTTAAACACTGGCGGTAATGCTGGTATTA------AATTTTATGT

HP0462        777  AGGCAAAGC-------------------TTCTTAT--------------TCAACGGATACTTGGTGTATT
jhp0414       847  CAATATGATA--TTATGATGGACAAATACGGAGAA-GCTGGATCCGTGCGTTTTGGACTTCAAGGGGCT-
HPAG1_0437    876  CAAAATGAAAGACACTAAAAAACATTTATTTTTAA-ACCAACACATGCAATCATGGATAAAAGAAAGCA-
HPG27_419     798  AGGCAAAGC-------------------TTCTTAT--------------TCAACGGATACTTGGTGTATT
HPP12_0434    838  GGGAATGGAA-----------------ATTTTTAT-GT---TACACACTATGATGGGAAATTTGACGCC-
HPSH_02265    772  GGGAATGGAAATTTTTATGTTACACATTATGATGG-GAAATT--------TGATGCCTATCAAAGAACA-
HPF16_0441    856  CAATATGATA--TTATGATAGACAAATACGGAGAG-GCCGGAGCCGTGCGTTTTGGACTTCAAGGGTCT-
HPF30_0860    810  CAAAATGAAAGACACTAAAAAACACTTATTTTTAA-ACCAACACATGCAATCATGGATAAAAGAAAGCA-
HPF32_0871    787  CAATGCGGAATAT-------CGTATCTAACTTT---ACCAA-------AATCTTGGATTACAGGTAATG-
HPF57_0489    829  GGGAATGGAA-----------------ATTTTTAT----GTTACACACTATGATGGGAAATTTGATGCC-
KHP_0426      777  AGGCAAAGC-------------------TTCTTAT--------------TCAACGGATACTTGGTGTATT
HPKB_0443     798  -ATTATGATAGACA----AATACGGAGAGGCTGGA-GCCGTG------CGTTTTGGACTTCAAGGGGCT-
hp908_0454    550  CAATGCGGAATAT-------------CATATTTAACATTGCCA----AAGTCTTGGATTACAGGTAATGC
HELPY_0444    856  AAATATTGCCTATTAGAGCCAACAAATATTTTAATCACATTAACAGGGCATATTGGACGCTGTGCCTTAG
HPB8_1123     786  AGGCAAAGC-------------------TTCTTAT--------------TCAACGGATACTTGGTGTATT
HPCU_02530    829  GGGAATGGAA-----------------ATTTTTAT----GTTACACATTATGATGGGAAATTTGATGCC-
HPPC_02215    847  AAATATTGCTTATTAGAGCCAACAAATATTTTAATCACATTAACAGGACATATTGGACGCTGTGCCTTAG
HPSJM_02310   841  AAATATTGCCTATTAGAGCCAACAAATATTTTAATCACATTAACAGGACATATTGGACGCTGTGCCTTAG
HPSAT_02200   868  AAATATTGCCTATTAGAGCCAACAAATATTTTAATCACATTAACAGGACATATTGGACGCTGTGCCTTAG
HPV225_0460   771  AGGCAAAGC-------------------TTCTTAT--------------TCAACGGATACTTGGTGTATT

HP0462        814  TGCGCT--AACGAATTTAGCGAC------TATTTATATTTACTGCTCTCAAGTATAAAAA-----ACCAT
jhp0414       913  TATAAT--GTTGCCTTAAGTAAAAT-TAGCGTATTAAATCAATCCATGCAGGAATATATA-CGCAGTTAT
HPAG1_0437    944  TATTATCCACAGGTTTTTGTGGCTTGCAATG-TCAAAAACA-TACTTTTGAATACATA-G-CTTCCACGA
HPG27_419     835  GGCGCT--AACGAATTTAGCGAC------TATTTATATTTACTGCTCTCAAGCATAAAAA-----ACCAT
HPP12_0434    886  TATCAAAGAACATATGTTGTAAATCCAAATAATCCAAATCACTATGTTTTAATATATCTA-TTTGTAAAA
HPSH_02265    832  TATGTT--GTAAGTCCAAATAA----------TCCAAATCACTATGTTTTAATATATTTA-TTTGTAAAA
HPF16_0441    922  TATAAT--GTTGCCTTAAGTAAAAT-TAGCGTATTAAATCAATCCATGCAGGAATATATA-CGCAGTTAT
HPF30_0860    878  TATTATCCACAGGTTTTTGTGGCTT-ACA-ATGTCAAA--AACATACTTTTGAATACATAGCTTCCACGA
HPF32_0871    839  CGATGGTAATAAGACCTACTAAATC-TTATACTTCTAA--AACTTATCTTT-ATCACACAATAAAGAAAT
HPF57_0489    877  TATCAAAGAACATATGCTGTAAGTCCAAATAATCCAAATCATTATGTTTTAATATATTTA-TTTGTAAAA
KHP_0426      814  TGCGCT--AACGAATTTAGCGAC------TATTTATATTTACTGCTTTCAAGTATAAAAA-----CCCAT
HPKB_0443     856  TATAAT--GTTGCCTTAAGTAAAAT-TAGCGTAATAAATCAATCCATGCAGGAATATATA-CGCAGTTAT
hp908_0454    603  GATGGT-AATACGACCTACTAAATC-TTATACTTCTAA--AACTTATCTTT-ATCACACAATAAAGAAAT
HELPY_0444    926  TGTTTTCAAAAAATTGTATTTTAAATCAGCGCGTCGGCGTAGT-TTTACCAAAAGAAAAA-GAGCTAAAC
HPB8_1123     823  TGTGCT--AACGAATTTAGCGAC------TATTTATATTTACTGCTCTCAAGTATAAAAA-----ACCAT
HPCU_02530    877  TATCAAAGAACATATGTTGTAAGTCCAAATAATCCAAATCACTATGTTTTAATATATTTA-TTTGTAAAA
HPPC_02215    917  TGTTTTCAAAAAATTGCATTTTAAATCAGCGCGTCGGCGTAGT-TTTACCAAAAGAAAAA-GAGCTAAAT
HPSJM_02310   911  TGTTTTCAAAAAATTGCATTTTAAATCAGCGTGTCGGCGTAGT-TTTACCAAAAGAAAAA-GAACTAAAC
HPSAT_02200   938  TGTTTTCAAAAAATTGTATTTTAAATCAGCGCGTCGGCGTAGT-TTTACCAAAAGAAAAA-GAGCTAAAC
HPV225_0460   808  TGTGCT--AACGAATTTAGCGAC------TATTTATATTTACTGCTTTCAAATATAAAAA-----CCCAT
```

```
HP0462       871  ATCAATCAAAGCTTTTTTCAAGGAA------CTAGCCTTA-------------AACAC-TTGCAAAAAAA
jhp0414      979  CTAAATTCAAAACCTATAAAAAAATA-----TCTTTCTAA---TGCTTGCATGGCAT-CTACGAGAGCA
HPAG1_0437   1010 TCAAATACTCCCCTTTTGAAACAAGAAAAA-ATAATCTAG---CGACCG-GTGCTAC-ACAAAAAGCTA
HPG27_419    892  ATCAATCAAAGCTTTTTTCAAGGAA------CTAGCCTTA-------------AACAC-TTACAAAAAAA
HPP12_0434   955  TCATATACAAATTACTTAAAATTA--------CAATCTCG---TGGCT-----CTAT-TATTAAATTTA
HPSH_02265   889  TCATATACAAATTACTTAAAATTACAATCTCGTGGTTCTA---TTATTA-AATTTAT-TACAAAATCAG
HPF16_0441   988  CTAAATTCAAAGCCTATAAAAAAAT---A-TCTTTCTAA---TGCTTGCATGGCAT-CTACAAGATCA
HPF30_0860   944  TTAAATACTCCCCTTTTGAAACAAGAAAAA-ATAATCTAG---CAACCG-GCGCTAC-ACAAAAAGCTA
HPF32_0871   905  ACAAGCTCACAAATTATATAACTGGA-----TCCGTTCAA---CCCCAA-ATTACAC-GACAAAATCTA
HPF57_0489   946  TCATATACAAATTACTTAAAATTA--------CAATCTCG--------TGGTTCTAT-TATTAAATTTA
KHP_0426     871  ATCAATCAAAGCTTTTTTCAAGGAA------CTAGCCTTA-------------AACAC-TTACAAAAAAA
HPKB_0443    922  CTAAATTCAAAACCTATAAAAAAATA-----TCTTTCTAA---TGCTTGCATGGCAT-CTACAAGATCA
hp908_0454   668  ACAAGCTCACAAACTATATAACTGGA-----TCCGTTCAA----CCCCAAATCACAC-GACAAAACCTA
HELPY_0444   994  CCCTTTTATTATTCTTTAATAAGAAATCCC-CTATTCTCAGCCATACTGCAGAGAAACGCTATAGGCTCA
HPB8_1123    880  ATCAATCAAAGCTTTTTTCAAGGAA------CTAGCCTTA-------------AACAC-TTACAAAAAAA
HPCU_02530   946  TCATATACAAATTACTTAAAATTA--------CAATCTCG--------TGGTTCTAT-TATTAAATTTA
HPPC_02215   985  CCCTTTTATTATTCTTTGATAAGAAATCCC-CTATTCTCAGCCATACTACAGAGAAACGCTATAGGATCA
HPSJM_02310  979  CCCTTTTATTATTCTTTGATAAGAAATCCC-CTATTCTCAGCCATACTACAGAGAAACGCTATAGGATCA
HPSAT_02200  1006 CCCTTTTATTATTCTTTGATAAGAAATCCC-CTATTCTCAGCCATACTACAGAGAAACGCTATAGGATCA
HPV225_0460  865  ATCAATCAAAGCTTTTTTCAAGGAA------CTAGCCTTA-------------AACAC-TTACAAAAAAA

HP0462       921  TTT--ACTTAAAAAAATATCCTA---------------TTTACATGCCGTCCGTGCATGAA--ATTAAAAA
jhp0414      1039 TC---ACTAAATGAAAATCATATTTATTCTTTAATGCTCCCCATACCA---CCTATTAATCTATTACAAA
HPAG1_0437   1073 TC---A-ACATTGAGATGCTAGACTATATTTTCAT---TCTTATACCA---AATAAAGAGCTACTAGATA
HPG27_419    942  TTT--GCTTAAAAAAATATCCTA---------------TTTACATGCCGTCCGCGCATGAA--ATTAAAAA
HPP12_0434   1007 TT---ACAAAATCAGATATTGAAA-ATATTAAAAT--CGTATTACCA---AAT---------TTAAAAA
HPSH_02265   953  AT---ATTGA--AGATATTAAA--------------ATCGTATTACCA---AATTTAAAAACCTATGCCA
HPF16_0441   1048 TC---ACTAAATGAAAATCATATTTATTCTTTAATGCTCCCCATACCA---CCTATTAATCTATTACAAA
HPF30_0860   1007 TC---AACATTGAGATGCTAGACTATATTTTCA----TTCTTATACCA---AATAAAGAGCTACTAGATA
HPF32_0871   964  TC---CACA-----ATGCCA------------A----TTCTTATACCC---AAAAGAAAAACCCTAAATA
HPF57_0489   998  TT---ACAAAATCAGATATTGA---AAATATTAAA-ATCGTATTACCA---AATTTAAAAACCTATGCCA
KHP_0426     921  TTT--GCTTAAAAAAATATCCTA---------------TTTACATGCCGTCCGCGCATGAA--ATTAAAAA
HPKB_0443    982  TC---ACTAAATGAAAATCATATTTATTCTTTAATGCTCCCCATACCA---CCTATTAATCTATTACAAA
hp908_0454   727  TC-------CACAATGCCAA----------------TTCTTATACCC---AAAAGAAAAATCCTAAATA
HELPY_0444   1063 TCTCAACAAAATTTAAGCCCTATTGACACCTTAAAAATTCAAATCCCTTTCAACCATAAAATAATCAAGC
HPB8_1123    930  TTT--GCTTAAAAAAATATCCTA---------------TTTACATGCCGTCCGCGCATGAA--ATTAAAAA
HPCU_02530   998  TT---ACAAAATCAGATATTGA---AAATATTAAA-ATCGTATTACCA---AATTTAAAAACCTATGCCA
HPPC_02215   1054 TCTCAACAAAATTTAAGCCCTATTGACACCTTAAAAATTCAAATCCCTTTCAACCATAAAATAATCAAGC
HPSJM_02310  1048 TCTCAACAAAATTTAAGCCCTATTGACACCTTAAAAATTCAAATCCCTTTCAACCATAAAATAATCAAGC
HPSAT_02200  1075 TCTCAACAAAATTTAAGCCCTATTGACACCTTAAAAATTCAAATCCCTTTCAACCATAAAATAATCAAGC
HPV225_0460  915  TTT--GCTTAAAAAAATATCCTA---------------TTTACATGCCGTCCGTGCATGAA--ATTAAAAA

HP0462       972  ATTTAATCAAATTATGATGCC--CCTACTCACGCTTATATCCATTAACACAAGAACTTCTAAAAAATTAG
jhp0414      1103 AATACGAAAAAATCGCAAAAAATATCAT-AACAGCCATTATTAAAAACAATCAATCAACCCAAACCCTAA
HPAG1_0437   1133 ATTATTCTAAAATAACTAAACCACTCTA-TGAAAAAATCTCTAATAATATCATTGAAGCCCAAACCCTAA
HPG27_419    993  ATTTAATCAAATTATTATGCC--CCTACTCACGCTTATATCCATTAACACAAGAACTTCTAAAAAATTAG
HPP12_0434   1058 CTTATACCAAATGGAACAATGTATTA---AAAATAATTGAGAATAACAACCAATCAACCCAAACCCTAA
HPSH_02265   1001 AATGGAACAATGTATTAAAAA-------------TGATTGAGAACAACATGCAATCAACCCAAACCCTAA
HPF16_0441   1112 AATACGAAAAAATCGCAAAAAATATCAT-AACAGCTATTATTAAAAACAATCAATCAACCCAAACCCTAA
HPF30_0860   1067 ATTATTCTAAAATAACTAGAC-CACTCTATGAAAAAATCTCTAATAATATCATTGAAACCCAAACCCTAA
HPF32_0871   1007 AGTGGAATAATATATCTAGTC-TATTATGGAATTTAATTCACAATAACATGCAATCAACCCAAACCCTAA
HPF57_0489   1058 AATGGAACAATGTATTAAAAA-------------TGATTGAGAACAACAATCAATCAACCCAAACCCTAA
KHP_0426     972  ATTTAATCAAATTATTATGCC--CCTACTCACGCTTATATCCATTAACACAAGAACTTCTAAAAAATTAG
HPKB_0443    1046 AATACGAAAAAATCGCAAAAAATATCATAACAGCTA-TTATTAACAACAACCAATCAACCCAAACCCTAA
hp908_0454   770  AGTGGAATAATATATCTAGTC-TATTATGGAATTTAATTCATAGTAACATGCAATCAACCCAAACCCTAA
HELPY_0444   1133 AATACTCAAAAACATGCGAAAATATCAT-TAAATTACTTGTATCCAACATGCAAGCAACCCAAACCCTAA
HPB8_1123    981  ATTTAATCAAATTATGATGCC--CCTACTCACGCTTATATCCATTAACACAAGAACTTCTAAAAAATTAG
HPCU_02530   1058 AATGGAACAATGTATTAAAAA-------------TGATTGAGAACAACATGCAATCAACCCAAACCCTAA
HPPC_02215   1124 AATACTCAAAAACATGCGAAAATATCAT-TAAATTACTTGTATCCAACATGCAAACAACCCAAACCCTAA
HPSJM_02310  1118 AATACTCAAAAACATGCGAAAATATCAT-TAAATTACTTGTATCCAACATGCAATCAACCCAAACCCTAA
HPSAT_02200  1145 AATACTCAAAAACATGCGAAAATATCAT-TAAATTACTTGTATCCAACATGCAATCAACCCAAACCCTAA
HPV225_0460  966  ATTTAATCAAATTATTATGCC--CCTACTCACGCTTATATCTATTAACACAAGAACTTCTAAAAAATTAG
```

conserved region

```
HP0462        1040 AACAAATCAGAGACTTCCTACTCCCCCTACTCTTAAAACAGCAAGTCAAACCCCAATGA
jhp0414       1172 CCGCGCTCAGAGACTTTCTACTCCCCCTACTCTTAAAACAACAAGTCAAACCACAATGA
HPAG1_0437    1202 CCGCGCTCAGAGACTTTCTACTCCCCCTACTCTTAAAACAGCAAGTCAAACCTAAATAA
HPG27_419     1061 AACAAATCAGAGACTTCCTACTCCCCCTACTCCTAAAACAACAAGTCAAACCCCAATGA
HPP12_0434    1124 CCGCGTTCAGGGACTTCCTACTCCCCCTACTCTTAAAGCAACAAGTCAAACCCCAATGA
HPSH_02265    1058 CCGCGCTCAGAGATTTTCTACTCCCCCTACTCCTAAAACAGCAAGTCAAACCCCAATGA
HPF16_0441    1181 CCGCGCTCAGAGATTTTCTACTCCCCCTACTCCTAAAACAACAAGTCAAACCCCAATGA
HPF30_0860    1136 CCGCACTCAGAGATTTTCTACTCCCCTTACTCCTAAAACAACAAGTCAAACCCCAATGA
HPF32_0871    1076 CCGCGCTCAGAGATTTTCTACTCCCCCTACTCCTAAAGCAACAAGTCAAACCTAAATAA
HPF57_0489    1115 CCGCGCTCAGAGATTTTCTACTCCCCCTACTCCTAAAACAACAAGTCAAACCCCAATGA
KHP_0426      1040 AACAAATCAGAGATTTTCTACTCCCCCTACTCCTAACACAACAAGTCAAACCCCAATGA
HPKB_0443     1115 CCGCGCTCAGAGATTTTCTACTCCCCCTACTCCTAAAACAACAAGTCAAACCTAAATAA
hp908_0454    839  CCGTGCTCAGAGACTTTCTACTCCCCCTACTCTTAAAACAACAAGTCAAACCACAATGA
HELPY_0444    1202 CCACGCTCAGAGACTTTCTGCTCCCCCTACTCTTAAAACAACAAGTCAAACCTAAATAA
HPB8_1123     1049 AACAAATCAGAGACTTCCTACTCCCCCTACTCTTAAAACAACAAGTCAAACCCCAATGA
HPCU_02530    1115 CCGCACTCAGAGATTTTCTACTCCCCCTACTCCTAAAACAGCAAGTCAAACCCCAATGA
HPPC_02215    1193 CCGCACTGAGAGATTTTCTACTCCCCCTACTCTTAAAACAACAAGTCAAACCCCAATGA
HPSJM_02310   1187 CCGCGCTCAGAGATTTTCTACTCCCCCTACTCCTAACACAACAAGTCAAACCCTAA---
HPSAT_02200   1214 CCGCGCTCAGAGATTTTCTACTCCCCCTACTCCTAAAACAACAAGTCAAACCCCAATGA
HPV225_0460   1034 AACAAATCAGAGATTTTCTACTCCCCCTACTCTTAAAACAGCAAGTCAAACCCCAATGA
```
