# Supplementary material for: Domain Movement within a Gene: A Novel Evolutionary Mechanism for Protein Diversification
Source: PLoS One. 2011 Apr 14;6(4):e18819. doi: 10.1371/journal.pone.0018819 (PMC3077401; doi:10.1371/journal.pone.0018819)
Supplement: Figure S2 — Nucleotide sequence alignment of Group 2 S (See Fig. 3 ). (PDF) [file pone.0018819.s002.pdf]

```
                                        conserved region       repeat x
locus1_HP0790                 1    ATGCATAAAATAGAGCGCTTACTCCAAACTCTAGCGCCTAAGGGGGTGGAGTTTAAAACG
locus1_jhp0726                1    ATGAACAAAATAGAGTTATTACTCCACACTCTAGCACCTAAGGGGGTGGAGTTTAGGAAG
locus1_HPAG1_0775             1    ATGCATAAAATAGAGCGCTTACTCCAAACTCTAGCGCCTAAGGGGGTGGAGTTTAGGAAG
locus1_HPG27_746              1    ATGCATAAAATAGAGCGCTTACTCCAAACCTTAGCGCCTAAGGGGGTGGAGTTTAAAACG
locus1_HPP12_0797             1    ATGCATAAAATAGAGCGACTGCTCCACACTCTAGCGCCTAAGGGGGTGGGGTTTAGGAAG
locus1_HPSH_02865             1    ATGCATAAAATAGAGCGCTTACTCCAAACTTTAGTGCCTAAGGGGGTGGAGTTTAAAACG
locus1_HPF16_0572             1    ATGCATAAAATAGAGCGCTTACTCCAAACTTTAGCGCCTAAGGGGGTGGAGTTTAGGAAA
locus1_HPF30_0541-HPF30_0542  1    ATGCATAAAATAGAGCGCTTACTCCAAACTTTAGCGCCTAAGGGGGTGGAGTTTAGGAAG
locus1_HPF32_0757             1    ATGCATAAAATAGAGCACTTACTCCAAACTTTAGCGCCTAAGGGGGTGGAGTTTAGGAAG
locus1_HPF57_0810             1    ATGCATAAAATAGAGCGCTTACTCCAGACTTTAGCGCCTAAGGGGGTGGAGTTTAAAACG
locus2_HP0848-HP0849          1    ATGCATAAAATAGAGCAACTGCTCCAAACTCTAGCGCCTAAGGGGGTGGAGTTTAGGAAG
locus2_jhp0785                1    ATGAATAAAATAGAGCGA------------------------------------------
locus2_HPAG1_0832             1    ATGCATAAAATAGAGCGCTTGCTCCAAACTCTAGCGCTTAAGGGGGTGGGGTTTAGGAAG
locus2_HPG27_805              1    ATGCATAAAATAGAGCGCTTACTCCAAACCTTAGCGCCTAAGGGGGTGGAGTTTAAAACG
locus2_HPP12_0849             1    ATGCATAAAATAGAGCGCTTACTCCAAACTTTAGCGCCTAAGGGGGTGGAGTTTAGGAAA
locus2_HPSH_02565             1    ATGCATAAAATAGAGCGCTTACTCCAAACTTTAGTGCCTAAGGGGGTGGAGTTTAAAACG
locus2_HPF16_0513             1    ATGCATAAAATAGAGCGCTTACTCCAAACTTTAGCGCCTAAGGGGGTGGAGTTTAAAACG
locus2_HPF30_0484             1    ---------------------------------------------GTGGAGTTTAAAACG
locus2_HPF32_0814             1    ATGCATAAAATAGAGCGCTTACTCCAAACTTTAGCGCCTAAGGGGGTGGAGTTTAGGAAG
locus2_HPF57_0869             1    ATGCATAAAATAGAGCGCTTACTCCAAACTTTAGCGCCTAAGGGGGTGGAGTTTAGGAAG
                                   TRD1
locus1_HP0790                 61   CTTGAAGAGGTTTTTGAAATTAAAAA---TGGTTACACCCCATCAAAAAACAATCCTGAA
locus1_jhp0726                61   TTGGGGGATATTGGGGAATTTTA-------TGGCGGACTTGTTGGAAAAAGTAAAAAATC
locus1_HPAG1_0775             61   TTGGGGGAGGT--------------------GCTAGAGTATGATCAACCCAA--------
locus1_HPG27_746              61   CTTGAAGAGGTTTTTGAAATTAAAAA---TGGTTACACCCCATCAAAAAACAATCCTGAA
locus1_HPP12_0797             61   TTGGGGGAGAT--------------------TCTAGAGTATGATCAACCCAA--------
locus1_HPSH_02865             61   CTTGAAGAGGTTTTTGAAATTAAAAA---TGGTTACACCCCATCAAAAAACAATCCTGAA
locus1_HPF16_0572             61   TTGGGGGAGGT-------------------GTGTGAAAGCACAAATAAAAAAACACTCAA
locus1_HPF30_0541-HPF30_0542  61   TTGGGGGAGGT--------------------GTGTGATTTTCAAAAAGGAAAA-------
locus1_HPF32_0757             61   TTGGGGGAGGT-------------------GTGTGAAATTTTAGATAATCGGC-------
locus1_HPF57_0810             61   CTTGAAGAGGTTTTTGAAATTAAAAA---TGGTTACACCCCATCAAAAAACAATCCTGAA
locus2_HP0848-HP0849          61   TTGGGGGATATTATCTTATCGTTAAAAACCGGATTAAATCCAAGAAAATTTTTTAAACTT
locus2_jhp0785                18   ------------------------------------------------------------
locus2_HPAG1_0832             61   TTGGGGGAGGT--------------------GTGTGATTTTCAAAAAGGAAAA-------
locus2_HPG27_805              61   CTTGAAGAGGTTTTTGAAATTAAAAA---TGGTTACACCCCATCAAAAAACAATCCTGAA
locus2_HPP12_0849             61   TTGGGGGAGGT--------------------GTGTGATTTTCAAAAAGGAAAG-------
locus2_HPSH_02565             61   CTTGAAGAGGTTTTTGAAATTAAAAA---TGGTTACACCCCATCAAAAAACAATCCTGAA
locus2_HPF16_0513             61   CTTGAAGAGGTTTTTGAAATTAAAAA---TGGTTACACCCCATCAAAAAACAATCCTGAA
locus2_HPF30_0484             16   CTTGAAGAGGTTTTTGAAATTAAAAA---TGGTTACACCCCATCAAAAAACAATCCTGAA
locus2_HPF32_0814             61   TTGGGGGAGGT--------------------GTGTGATTTTCAAAAAGGAAAA-------
locus2_HPF57_0869             61   TTGGGGGAGGT--------------------GCTAGAATATAATCAACCCAA--------

locus1_HP0790                 118  TTTTGGAAAAATGGGACTATCCCTTGGTTTAGAATGGAAGACATTAGAGAAAATGGGAGG
locus1_jhp0726                114  TTTCTCTCAAGGGAATAAATTTTATGTTCCTTATATCAATGTTTTTAATAATCCACAATT
locus1_HPAG1_0775             92   --TCAATATTGCGTAACGAGT--------------AAAGAATTTGATAAAAGTTATCCTA
locus1_HPG27_746              118  TTTTGGAAAAATGGGACTATCCCTTGGTTTAGAATGGAGGACATTAGAGAAAATGGGAGG
locus1_HPP12_0797             92   --TCAATATTGCGTAACGAGT--------------AAAGAATTTGATAAAAGTTATCCTA
locus1_HPSH_02865             118  TTTTGGGAAAAAGGGACTATCCCTTGGTTTAGAATGGAAGACATTAGAGAAAATGGGAGG
locus1_HPF16_0572             102  AATAAGCGAAGTGAGCGAAGT----------------AAAAAATAAGGGAATGTATCCAG
locus1_HPF30_0541-HPF30_0542  93   --TCAATA--ACAAAAAAAGC--------------CGTAACCTTTGGAAAAGT-------
locus1_HPF32_0757             94   -GTATCCCAATTGCGAAAAAT--------------AAAAGAAATCCGGGAATTTATCCTT
locus1_HPF57_0810             118  TTTTGGGAAAAAGGGACTATCCCTTGGTTTAGAATGGAGGACATTAGAGAAAATGGGAGG
locus2_HP0848-HP0849          121  AATACCCCTAATGCAAATAATTACTATGTAACTGTGAGAGAATTAGAAGAATATACTATA
locus2_jhp0785                18   ------------------------------------------------------------
locus2_HPAG1_0832             93   --TCAATA--ACAAAAAAAGC--------------CGTAACTTTTGGAAAAGT-------
locus2_HPG27_805              118  TTTTGGAAAAATGGGACTATCCCTTGGTTTAGAATGGAGGACATTAGAGAAAATGGGAGG
locus2_HPP12_0849             93   --TCAATA--ACAAAAAAAGC--------------CGTAACTTTTGGAAAAGT-------
locus2_HPSH_02565             118  TTTTGGGAAAAAGGGACTATCCCTTGGTTTAGAATGGAAGACATTAGAGAAAATGGGAGG
locus2_HPF16_0513             118  TTTTGGGAAAAAGGGACTATCCCTTGGTTTAGAATGGAGGACATTAGAGAAAATGGGAGG
locus2_HPF30_0484             73   TTTTGGGAAAAAGGGACTATCCCTTGGTTTAGAATGGATGACATTAGAGAAAATGGGAGG
locus2_HPF32_0814             93   --TCAATA--ACAAAAAAAGC--------------CGTAACTTTTGGAAAAGT-------
locus2_HPF57_0869             92   --TAAATATTGCGTAACGGGT--------------AAAGAATTTGATGAAAGTTATCCTA
```

```
locus1_HP0790                   178  ATTTTAAAAGACTCTATCCAACACATTACCCCAAAGGCTTTAAAGGGTAAGAAATTATTC
locus1_jhp0726                  174  AGATCTAAATGCT-TTAGAAAGCGTTCAAATAGGGGATAAAGAAAAACAAAATACAATTC
locus1_HPAG1_0775               137  CCCCCGTTTTAACCGCAGGAAAAACCTTTATTTTAGGTTATACAAACGAAAAAGACAATA
locus1_HPG27_746                178  ATTTTAAAAGACTCTATCCAACACATTACCCCAAAGGCTTTAAAGGGTAAGAAATTATTC
locus1_HPP12_0797               137  CCCCTGTTTTAACCGCAGGAAAAACCTTTATTTTAGGTTATACAAACGAAAAAGACAATA
locus1_HPSH_02865               178  ATTTTAAAAGACTCTATCCAACACATTACCCCAAAGGCTTTAAAGGGTAAGAAATTATTC
locus1_HPF16_0572               146  TGA---------TAAATTCAGGGAGGGATTTGTATGGTTATTACCATGATTTTAACAATG
locus1_HPF30_0541-HPF30_0542    128  -CCCCGTTATTTCGGGGGGAAGACAACCCGCTT---ATTATCACAATGAGGTAAATCGTA
locus1_HPF32_0757               140  A-----------T-TATGGAGCAAATGGAATTCAAGATTAT-ATTGATAGCT-ATATTTT
locus1_HPF57_0810               178  ATTTTAAAAGACTCTATCCAACACATTACCCCAAAGGCTTTAAAGGGTAAGAAATTATTC
locus2_HP0848-HP0849            181  AAATTCA----CT-CATCAAACAGATAGAATTGATGACAAT-GCTCTTTCTTTATGTTTA
locus2_jhp0785                  18   ------------------------------------------------------------
locus2_HPAG1_0832               128  -CCCCGTTATTTCGGGGGGAAGACAACCCGCTT---ATTATCACAATGAGGCAAATCGTA
locus2_HPG27_805                178  ATTTTAAAAGACTCTATCCAACACATTACCCCAAAGGCTTTAAAGGGTAAGAAATTATTC
locus2_HPP12_0849               128  -CCCCGTTATTTCGGGAGGAAGACAACCCGCTT---ATTATCACAATGAGGCAAATCGTA
locus2_HPSH_02565               178  ATTTTAAAAGACTCTATCCAACACATTACCCCAAAGGCTTTAAAGGGTAAGAAATTATTC
locus2_HPF16_0513               178  ATTTTAAAAGACTCTATCCAACACATTACCCCAAAGGCTTTAAAGGGTAAGAAATTATTC
locus2_HPF30_0484               133  ATTTTAAAAGACTCTATCCAACACATTACCCCAAAGGCTTTAAAGGGTAAGAAATTATTC
locus2_HPF32_0814               128  -TCCCGTTATTTCGGGGGGAAGACAACCCGCTT---ATTATCACAATGAGGCAAATCGTA
locus2_HPF57_0869               137  CTCCCGTTTTAACCGCAGGGAAAACCTTTATTTTAGGTTATACAAACGAAAAAGACAATA

locus1_HP0790                   238  CCTAAA--AATTCTATTATTATTTCTACGAAAGCAACCATAGGAGA-GCATGCCCTTTTA
locus1_jhp0726                  233  AATTAGGCGATGTGCTTTTTACTGGTTCATCTGAAAATTTAGAGGA-TTGTGCGATGTCT
locus1_HPAG1_0775               197  TTTATC---AAGCGAGTAAAAACGCTCCGGTTATCATCTTTGACGA-TTTCACAACAGCG
locus1_HPG27_746                238  CCTAAA--AATTCTATTATTATTTCTACAACAGCAACGATAGGAGA-GCATGCCCTTTTA
locus1_HPP12_0797               197  TTTATC---AAGCGAGTAAAAGCTCTCCGGTTATCATCTTTGACGA-TTTCACAACAGCG
locus1_HPSH_02865               238  CCTAAA--AATTCTATTATTATTTCTACAACGGCAACGATAGGAGA-GCATGCCCTTTTA
locus1_HPF16_0572               197  ATGGAGAAAATATAACTATTGCATCTAGGGGAGAATATGCAGGATTTATAAACTACTTCA
locus1_HPF30_0541-HPF30_0542    185  GTGGAG---AAACAATAGCGATTTCTTCATCTGGAGTGTATGCTGG-CT--ATGTTAGTT
locus1_HPF32_0757               186  TGATGGTGATTTTGTGCTAGTTGGTGAAGATGGGAGTGTTATCAAT-AAGGATAATACTC
locus1_HPF57_0810               238  CCTAAA--AATTCTATTATGATTTCTACAACAGCAACCATAGGCGA-ACATGCCCTTTTA
locus2_HP0848-HP0849            235  AAACGCTCTAATCTTGAAAAAGATGATATATTTTATTCTCTGGAAC-TGGAACAATAGGA
locus2_jhp0785                  18   ------------------------------------------------------------
locus2_HPAG1_0832               185  GCGGAG---AAACAATAGCGATTTCTTCATCTGGAGTGTATGCTGG-CT--ATGTTAGTT
locus2_HPG27_805                238  CCTAAA--AATTCTATTATTATTTCTACAACAGCAACGATAGGAGA-GCATGCCCTTTTA
locus2_HPP12_0849               185  GCGGAG---AAACAATAGCGATTTCTTCATCTGGAGTGTATGCTGG-CT--ATGTTAGTT
locus2_HPSH_02565               238  CCTAAA--AATTCTATTATTATTTCTACAACGGCAACGATAGGAGA-GCATGCCCTTTTA
locus2_HPF16_0513               238  CCTAAA--AATTCTATTATTATTTCTACAACAGCAACCATAGGCGA-ACATGCCCTTTTA
locus2_HPF30_0484               193  CCTAAA--AATTCTATTATTATTTCTACAACAGCAACCATAGGCGA-ACATGCCCTTTTA
locus2_HPF32_0814               185  GCGGAG---AAACAATAGCGATTTCTTCATCTGGAGTGTATGCTGG-CT--ATGTTAGTT
locus2_HPF57_0869               197  TTTATC---AAGCGAGTAAAAGCTCTCCGGTTATCATTTTTGATGA-TTTCACAACAGCA

locus1_HP0790                   295  ATCGTTGATTCGTTAGCGAATCAACAATTCACTTTTTTAAGCAAAAAAGCGAATTGTGAT
locus1_jhp0726                  292  TGCGTAGTTACTCAAAAGATTGAGGAAGATATTTATCTTAATAGTTTTTGTTTTGGTTTT
locus1_HPAG1_0775               253  ACCCA-ATGGGTTGATTTCCCCTTCAAAGTAAAATCAAGCGCCATGAAAATCTTATTCTC
locus1_HPG27_746                295  ATCGTTGATTCGTTAGCGAATCAACAATTCACTTTTTTAAGCAAAAAAGCGAATTGTGAT
locus1_HPP12_0797               253  ACCCA-ATGGGTTGATTTCCCTTTCAAAGTAAAATCAAGCGCTATGAAAATCTTATTCTC
locus1_HPSH_02865               295  ATCGTTGATTCGTTAGCGAATCAACAATTCACTTTTTTAAGCAAAAAAGCGAATTGTGAT
locus1_HPF16_0572               257  ATGAAAAATTTTTTGCAGGGGGTCTATGTTATCCCTATAAAGTTAA-----AGATACTAA
locus1_HPF30_0541-HPF30_0542    239  ATTGGGATATTCCTGTTTTTCTCGCTGACTCTTTTTCTGTTTCACCAAAACAAAAAACCC
locus1_HPF32_0757               245  CTATTGTGAATTGGGCAAGCGGAAAAATATGGGTGAACAATCATG-------CTCATGTG
locus1_HPF57_0810               295  ATCGTTGATTCGTTAGCGAATCAACGATTCACTTTTTTAAGCAAAAAAGCGAATTGTGAT
locus2_HP0848-HP0849            294  AAAGTATCAATTATTAAAGAAAATCCAAATAATTGGGCGATTAAAGAGGGAATTTATTCA
locus2_jhp0785                  18   ------------------------------------------------------------
locus2_HPAG1_0832               239  ATTGGGATATTCCTGTTTTTCTTGCTGACTCTTTTTCTGTTTCACCAAAACAAAAAACCC
locus2_HPG27_805                295  ATCGTTGATTCGTTAGCGAATCAACAATTCACTTTTTTAAGCAAAAAAGCGAATTGTGAT
locus2_HPP12_0849               239  ATTGGGATATTCCTGTTTTTCTTGCTGACTCTTTTTCTGTTTCGCCAAAACAAAAAACCC
locus2_HPSH_02565               295  ATCGTTGATTCGTTAGCGAATCAACAATTCACTTTTTTAAGCAAAAAAGCGAATTGTGAT
locus2_HPF16_0513               295  ATCGTTGATTCGTTAGCGAATCAACAATTCACTTTTTTAAGCAAAAAAGCGAATTGTGGT
locus2_HPF30_0484               250  ATCGTTGATTCGTTAGCGAATCAACGATTCACTTTTTTAAGCAAAAAAGCGAATTGTAAT
locus2_HPF32_0814               239  ATTGGGATATTCCTGTTTTTCTTGCTGACTCTTTTTCTGTTTCGCCAAAACAAAAAACCC
locus2_HPF57_0869               253  ACCCA-ATGGGTTGATTTCCCTTTCAAAGTAAAATCAAGCGCTATGAAAATCTTACTCCC
```

```
locus1_HP0790                  355  CTTGCTTTAGACATGAAATTCTTTTT--------TTATCAATGTTTTCTTTTGGGGGAAT
locus1_jhp0726                 352  AGATTTTTTGATAAGAATTTATTTAATCCATCATTTCTTAAACATTTTTTAAGAGACTAC
locus1_HPAG1_0775              312  AAAAAATCCTACAA--TTAATATTAG----------ATTTATCTTTTTTTACATGCAAAT
locus1_HPG27_746               355  CTTGCTTTAGACATGAAATTCTTTTT--------TTACCAATGCTTTCTTTTAGGGGAAT
locus1_HPP12_0797              312  AAAAAATCCTACAA--TTAACATTAG----------ATTTATCTTTTTTTGCATGCAAAC
locus1_HPSH_02865              355  ATTGCTTTAGACATGAAATTCTTTTT--------TTATCAATGTTTTCTTTTGGGGGAAT
locus1_HPF16_0572              312  CGAGCTTTTAACAAAATTTTTATACT---------TTTATCTCAAAACTAATGAAATCCA
locus1_HPF30_0541-HPF30_0542   299  TAATGCCTAAATATCTTTTTCATTAT----------CTTACAACACAACAAGATGCGATC
locus1_HPF32_0757              298  CTTCAAACAAAAAATGAACTAAAATT---AAAGTTTTTGTATTTTTATTTACAAACGATA
locus1_HPF57_0810              355  CTTGCTTTAGACATGAAATTCTTTTT--------TTATCAATGTTTTCTTTTGGGGGAAT
locus2_HP0848-HP0849           354  ATCAAACCAAATAAAAAAATAGTATTTCCAAGATTTTTAATGTTTTGCTTTGAAAATTTG
locus2_jhp0785                 18   ------------------------------------------------------------
locus2_HPAG1_0832              299  TAATGCCTAAATATCTTTTTCATTAC----------CTTACAACGCAACAAGATGCGATC
locus2_HPG27_805               355  CTTGCTTTAGACATGAAATTCTTTTT--------TTACCAATGCTTTCTTTTAGGGGAAT
locus2_HPP12_0849              299  TAATGCCTAAATATCTTTTTCACTAT----------CTTACAACGCAACAAGATGCAATC
locus2_HPSH_02565              355  ATTGCTTTAGACATGAAATTCTTTTT--------TTATCAATGTTTTCTTTTGGGGGAAT
locus2_HPF16_0513              355  ATTGCTTTAGACATGAAATTCTTTTT--------TTATCAATGTTTTCTTTTAGGGGAAT
locus2_HPF30_0484              310  ATTGCTTTAGACATGAAATTCTTTTT--------TTATCAATGTTTTCTTTTGGGGGAAT
locus2_HPF32_0814              299  TAATGCCTAAATATCTTTTTCACTAT----------CTTACAACGCAACAAGATGCGATC
locus2_HPF57_0869              312  AAAAAATCCTACAA--TTAACATCAG----------ATTTATCTTTTTTTATATGCAAAC

locus1_HP0790                  407  GGTGC-AAAAATAATA------------TTAATGTTTCAGGTTTTGCTTCTGTGGATATG
locus1_jhp0726                 412  AATTTTAGGAAAAATATTTCAAAAGTTGCTAATGGTGTAACGCGCTTCAATGTTTCAAAG
locus1_HPAG1_0775              360  TATTCCTTATAATATC-----------------GGTGGGGAACATGCAAGGCAGTGGATT
locus1_HPG27_746               407  GGTGC-AAAAATAATA------------TTAATGTTTCAGGTTTTGCTTCTGTGGATATG
locus1_HPP12_0797              360  TATTCCTTATAATATC-----------------GGTGGGGAACATGCAAGGCATTGGATT
locus1_HPSH_02865              407  GGTGC-AAAAAAAACA------------CTAATGTTTCAGGTTTTGCTTCTATGGATATG
locus1_HPF16_0572              363  AATTATGGAGAACCTT-----------GTTTCTTGTGGCAGTATCCCCGCACTCAATAAA
locus1_HPF30_0541-HPF30_0542   349  CATGCAACAAAAAGCA-----------------CAGGGGGAATTCCTCATGT-TTATAGC
locus1_HPF32_0757              355  GATGTTAGCTATTGT-------------GTTGCTGGAACTCCGCCAAAAATCAACCAAGA
locus1_HPF57_0810              407  GGTGC-AAAAAAAATA------------CTAATGTTTCAGGTTTTGCTTCTGTGGATATG
locus2_HP0848-HP0849           414  AATATTCAAAATGATATAAAAAGTAAAAGTTTTGGAGGAATAGTTAAAAGTATATCAATG
locus2_jhp0785                 18   ------------------------------------------------------------
locus2_HPAG1_0832              349  CATGCAACAAAAAGCA-----------------CAGGAGGAATTCCTCATGT-TTATAGC
locus2_HPG27_805               407  GGTGC-AAAAATAATA------------TTAATGTTTCAGGTTTTGCTTCTGTGGATATG
locus2_HPP12_0849              349  CATGCAACAAAAAGCA-----------------CAGGGGGAATTCCTCATGT-TTATAGC
locus2_HPSH_02565              407  GGTGC-AAAAAAAACA------------CTAATGTTTCAGGTTTTGCTTCTATGGATATG
locus2_HPF16_0513              407  GGTGC-AAAAAAAATA------------CTAATGTTTCAGGTTTTGCTTCTGTGGATATG
locus2_HPF30_0484              362  GGTGC-AAAAAAAATA------------CTAATGTTTCAGGTTTTGCTTCTGTGGATATG
locus2_HPF32_0814              349  CATGCAACAAAAAGCA-----------------CAGGGGGAATTCCTCATGT-TTATAGC
locus2_HPF57_0869              360  TATTCCCTATAATATC-----------------AGTGGGGAACATACAAGGCAGTGGATT

                                                           repeat y
locus1_HP0790                  454  ACTGCTTTTAAAAAATATAAGTTCCCCATCCCACCCCTAGAGATCCAACAAGAGATCGTT
locus1_jhp0726                 472  CAATTACTTTTAAAAATAACCATCCCCATCCCGCCCCTAGAGATCCAGCAAGAGATCGTT
locus1_HPAG1_0775              403  TCTCGCTATTCACAATTAGAAGTCCCCATCCCACCCCTAGAGATCCAACAAGAGATCGTT
locus1_HPG27_746               454  ACTGCTTTTAAAAAATATAAGTTCCCCATCCCACCCCTAGAGATCCAACAAGAGATCGTT
locus1_HPP12_0797              403  TCTCGCTATTCACAATTAGAAGTCCCCATCCCACCCCTAGAGATCCAACAAGAGATCGTT
locus1_HPSH_02865              454  ACTGCTTTTAAAAAATATAAGTTCCCCATCCCACCCCTAGAAATCCAACAAGAGATCGTT
locus1_HPF16_0572              412  GCAGATATTGAAACTTTAACAATCCCCATCCCACCCCTAGAAATCCAACAAGAGATCGTT
locus1_HPF30_0541-HPF30_0542   391  AAAGACTTACAAAATTTTTTAATCCCCATCCCACCCCTAGAAATCCAACAAGAGATCGTT
locus1_HPF32_0757              402  AA--ATTTAAAAAAAAATAACAATCCCCATCCCACCCCTAGAAATCCAACAAGAGATCGTT
locus1_HPF57_0810              454  ACTGCTTTTAAAAAATATAAGTTCCCCATCCCACCCCTAGAAATCCAACAAGAGATCGTT
locus2_HP0848-HP0849           474  AACGATTTACAACAAATAACCATCCCCATCCCACCCCTAGAGATCCAACAAGAGATCGTT
locus2_jhp0785                 18   ------------------------------------------------------------
locus2_HPAG1_0832              391  AAAGACTTACAAAATTTTTTAATCCCCATCCCACCCCTAGAAATCCAACAAGAGATCGTT
locus2_HPG27_805               454  ACTGCTTTTAAAAAATATAAGTTCCCCATCCCACCCCTAGAGATCCAACAAGAGATCGTT
locus2_HPP12_0849              391  AAAGACTTACAAAATTTTTTAATCCCCATCCCACCCCTAGAGATCCAACAAGAGATCGTT
locus2_HPSH_02565              454  ACTGCTTTTAAAAAATATAAGTTCCCCATCCCACCCCTAGAAATCCAACAAGAGATCGTT
locus2_HPF16_0513              454  ACCGCTTTTAAAAAATATAAGTTCCCCATCCCACCCCTAGAAATCCAACAAGAGATCGTT
locus2_HPF30_0484              409  ACCGCTTTTAAAAAATATAAGTTCCCCATCCCACCCCTAGAAATCCAACAAGAGATCGTT
locus2_HPF32_0814              391  AAAGACTTACAAAATTTTTTAATCCCCATCCCACCCCTAGAAATCCAACAAGAGATCGTT
locus2_HPF57_0869              403  TCTCGCTATTCAAAAATAACAATCCCCATCCCACCCCTAGAAATCCAACAAGAGATCGTT
```

```
                                     conserved region        center repeat region
locus1_HP0790                   514  AAGATTTTGGACGCTTTCACAGAATTAAACACAGAATTAAACACAGAATTAAACACAGAA
locus1_jhp0726                  532  ACGATTTTGGACGCTTTCACAGAATTAAACACAGAATTAAACACAGAATTAAACACAGAA
locus1_HPAG1_0775               463  AAGATTTTGGACGCTTTCACAGAATTAAACACAGAATTAAACACAGAATTAAACACAGAA
locus1_HPG27_746                514  AAGATTTTGGACGCTTTCACAGAATTAAACACAGAATTAAACACAGAATTAAACACAGAA
locus1_HPP12_0797               463  AAGATTTTGGACGCTTTCACAGAATTAAACACAGAATTAAACACAGAATTAAACACAGAA
locus1_HPSH_02865               514  AAGATTTTGGACGCTTTCACAGAATTAAACACAGAATTAAAC------------ACAGAA
locus1_HPF16_0572               472  AAGATTTTGGACGCTTTCACAGAATTAAACACAGAATTAAACACAGAATTAAACACAGAA
locus1_HPF30_0541-HPF30_0542    451  AAGATTTTGGACGCTTTCACAGAATTAAACACAGAATTAAA-------------------
locus1_HPF32_0757               460  AATATTTTGGACGCTTTCACAGAATTAAACACAGAATTAAACACAGAATTAAAA------
locus1_HPF57_0810               514  AAGATTTTGGACGCTTTCACAGAATTAAACACAGAATTAAACACAGAATTAAACACAGAA
locus2_HP0848-HP0849            534  AAGATTTTGGACGCTTTCACAGAATTAAACACAGAATTAAACACAGAATTAAAA------
locus2_jhp0785                  18   ------------------------------------------------------------
locus2_HPAG1_0832               451  AAGATTTTGGACGCTTTCACAGAATTAAACACAGAATTAAACACAGAATTAAACACAGAA
locus2_HPG27_805                514  AAGATTTTGGACGCTTTCACAGAATTAAACACAGAATTAAACACAGAATTAAACACAGAA
locus2_HPP12_0849               451  AAGATTTTGGACGCTTTCACAGAATTAAACACAGAATTAAA-------------------
locus2_HPSH_02565               514  AAGATTTTGGACGCTTTCACAGAATTAAACACAGAATTAAACACAGAATTAAACACAGAA
locus2_HPF16_0513               514  AAGATTTTGGACGCTTTCACAGAATTAAACACAGAATTAAACACAGAATTAAACACAGAA
locus2_HPF30_0484               469  AAGATTTTGGACGCTTTCACAGAATTAAACACAGAATTAAACACAGAATTAAACACAGAA
locus2_HPF32_0814               451  AAGATTTTGGACGCTTTCACAGAATTAAACACAGAATTAAACACAGAATTAAA-------
locus2_HPF57_0869               463  AAGATTTTGGACGCTTTCACAGAATTAAACACAGAATTAAACACAGAATTAA--------

                                                                conserved region
locus1_HP0790                   574  TTAAA------------------------AGCGCGCAAAAAGCAATATGAATATTACCAA
locus1_jhp0726                  592  TTAAA------------------------CGCGCGAAAAAAGCAATATCAGTATTACCAA
locus1_HPAG1_0775               523  TTAAACACAGAATTAAACACAGAATTAAAAGCGCGCAAAAAGCAATACGAATATTACCAA
locus1_HPG27_746                574  TTAAACACAGAATTAA------------ACACGCGAAAAAAGCAATACCAGTATTACCAA
locus1_HPP12_0797               523  TTAA------------------------AAGCGCGCAAAAAGCAATATGAGTATTACCAA
locus1_HPSH_02865               562  TTAAA------------------------AGCGCGAAAAAAGCAATACCAGTATTACCAA
locus1_HPF16_0572               532  TTAAA------------------------AGCGCGAAAAAAGCAATACCAATATTACCAA
locus1_HPF30_0541-HPF30_0542    491  -----------------------------AGCGCGAAAAAAGCAATACCAGTATTACCAA
locus1_HPF32_0757               513  ------------------------------GCGCGCAAAAAGCAATACCAGTATTACCAA
locus1_HPF57_0810               574  TTAAA------------------------AGCGCGAAAAAAGCAATATGAGTATTACCAA
locus2_HP0848-HP0849            587  ------------------------------GCGCGCAAAAAGCAATATGAGTATTACCAA
locus2_jhp0785                  18   ------------------------------------------------------------
locus2_HPAG1_0832               511  TTAAACACAGAATTAA------------ACGCGCGCAAAAAGCAATACCAGTATTACCAA
locus2_HPG27_805                574  TTAAA------------------------CACGCGAAAAAAGCAATACCAGTATTACCAA
locus2_HPP12_0849               491  -----------------------------CACAGAATTAAAAGCGCTTAAATCCATCATA
locus2_HPSH_02565               574  TTAAA------------------------AGCGCGAAAAAAGCAATACCAGTATTACCAA
locus2_HPF16_0513               574  TTAAA------------------------AGCGCGAAAAAAGCAATACCAATATTACCAA
locus2_HPF30_0484               529  TTAAA------------------------AGCGCGAAAAAAGCAATACCAGTATTACCAA
locus2_HPF32_0814               503  -----------------------------AGCGCGCAAAAAGCAATACCAGTATTACCAA
locus2_HPF57_0869               514  ----------------------------AAGCGCGAAAAAAGCAATACCAGTATTACCAA

locus1_HP0790                   610  AACATGCTTTTAGACTTTAACGATATTAACCAAAGCCACAAAGACGCCAAAGAAAGATTG
locus1_jhp0726                  628  AACATGCTTTTAGACTTTAACGACATCAACCAAAGCCGCAAAGACGCCAAAGAAAGATTG
locus1_HPAG1_0775               583  AACATGCTTTTAGACTTCAATGACATTAATTCAAACCACAAAGACGCC-----AAAATGA
locus1_HPG27_746                622  AACATGCTTTTAGACTTCAATGATATTAATCAAAACCATAAAGACGCCAAAGAAAAATTA
locus1_HPP12_0797               559  AACATGCTTTTAGACTTCAATGACATTAATTCAAACCACAAAGACGCCAAAGAAAAATTA
locus1_HPSH_02865               598  AACATGCTTTTAGACTTTAAGGACATCCATTCAAACCATAAAGACGC-----AAAAATTA
locus1_HPF16_0572               568  AACATGCTTTTAGACTTTAAGGGCATCAATCAAAACCACAAAGACGC-----AAAAATGA
locus1_HPF30_0541-HPF30_0542    523  AACATGCTTTTAGACTTTAAGGGCATTCATTCAAACCACAAAGACGC-----AAAAATGG
locus1_HPF32_0757               544  AACATGCTTTTAGACTTTAAGGACACCAACCAAAACCACCAAGACGCAAAAA------TG
locus1_HPF57_0810               610  AACATGCTTTTAGACTTTAAGGGCATCCATTCAAACCACAAAGACGC-----AAAAATGA
locus2_HP0848-HP0849            618  AACATGCTTTTAGACTTTAACGATATTAATCAAAACCACAAAGACGCCAAAA------T-
locus2_jhp0785                  18   ------------------------------------------------------------
locus2_HPAG1_0832               559  AACATGCTTTTAGACTTTAATGATATTAACCAAAACCACAAAGACGC-----CAAAATGA
locus2_HPG27_805                610  AACATGCTTTTAGACTTCAATGATATTAATCAAAACCATAAAGACGCCAAAGAAAAATTA
locus2_HPP12_0849               523  AAGGCGTTTTAA------------------------------------------------
locus2_HPSH_02565               610  AACATGCTTTTAGACTTTAAGGACATCCATTCAAACCATAAAGACGC-----AAAAATTA
locus2_HPF16_0513               610  AACATGCTTTTAGACTTTAAGGGCATCAATCAAAACCACAAAGACGC-----AAAAATGA
locus2_HPF30_0484               565  AACATGCTTTTAGACTTTAAGGGCATTCATTCAAACCACAAAGACGC-----AAAAATGG
locus2_HPF32_0814               535  AACATGCTTTTAGACTTTAAGGACACCAACCAAAACCACCAAGACGC-----AAAAATGA
locus2_HPF57_0869               547  AACATGCTTTTAGACTTTAAGGGCATTAATTCAAACCACAAAGACGC-----AAAAAT--
```

```
                                                                  repeat x
locus1_HP0790                    670  GCGCAAAAAACCTACCCTAAACGCTTGAAAACC-TTACTCCAAACTCTAGCGCCTAAGGG
locus1_jhp0726                   688  GCGCAAAAACCCTACCCCAAACGCTTGA-AACAACTCCTCCACACTCTAGCGCCTAAGGG
locus1_HPAG1_0775                638  GCGC-CAAACCCTACCCCAAACGCTTGAAAACC-TTACTCCAAACTCTAGCGCCTAAGGG
locus1_HPG27_746                 682  GCGTGCAAAACCTACCCCAAACGCTTAAAAACC-TTACTCCAAACTCTAGCGCCTAAGGG
locus1_HPP12_0797                619  ACGCAAAAAACCTACCCTAAACGCTTGAAAACC-TTACTCCAAACATTAGCGCCTAAGGG
locus1_HPSH_02865                653  GCGCAAAAA-CCTACCCCAAACGCTTAAAAACC-TTACTCCAAACCTTAGCGCCTAAGGG
locus1_HPF16_0572                623  GCGC-AAAACCCTACCCCAAACGCTTGAAAACC-TTACTCCAAACTTTAGCGCCTAAGGG
locus1_HPF30_0541-HPF30_0542     578  GCGC-AAAACCCTACCCTAAACGCTTGAAAACC-TTACTCCAAACCTTAGCGCCTAAGGG
locus1_HPF32_0757                598  AGCGCAAAACCCTACCCTAAACGCTTGAAAACC-TTACTCCAAACTTTAGCGCCTAAGGG
locus1_HPF57_0810                665  GCGCAAAAA-CCTACCCCAAACGCTTAAAATCG-TTACTCCAAACTTTAGCGCCTAAGGG
locus2_HP0848-HP0849             670  -----AAAAACCTACCCTAAACGCTTGAAAACC-TTACTCCACACTTTAGCGCCTAAGGG
locus2_jhp0785                   18   ----------------------------------TTACTCCAAACTCTAGCGCCTAAGGG
locus2_HPAG1_0832                614  GCGC-TAAAACCTACCCTAAACGCTTAAAAACC-TTACTCCAAACTCTAGTGCCTAAGGG
locus2_HPG27_805                 670  GCGTGCAAAACCTACCCCAAACGCTTAAAAACC-TTACTCCAAACTCTAGCGCCTAAGGG
locus2_HPP12_0849                534  ------------------------------------------------------------
locus2_HPSH_02565                665  GCGCA-AAAACCTACCCCAAACGCTTAAAAACC-TTACTCCAAACCTTAGCGCCTAAGGG
locus2_HPF16_0513                665  GCGCA-AAACCCTACCCCAAACGCTTGAAAACC-TTACTCCAAACTTTAGCGCCTAAGGG
locus2_HPF30_0484                620  GCGCAAAAC-CCTACCCTAAACGCTTACAAACC-TTACTCCAAACCTTAGCGCCTAAGGG
locus2_HPF32_0814                590  GCGC-AAAACCCTACCCTAAACGCTTGAAAACC-TTACTCCAAACTTTAGCGCCTAAGGG
locus2_HPF57_0869                599  -----AAAAACCTACCCCAAACGCTTGAAAACC-TTACTCCAAACTTTAGCGCCTAAGGG
                                                TRD2
locus1_HP0790                    729  GGTGGAGTTTAGGAAGTTGGGGGAGGTGTGTGAAATTTTAGA---------------TAA
locus1_jhp0726                   747  AGTGGAGTTTAGGAAGTTGGGGGATATTGGGGAATTTACTAGAGGTA-------ATGGGC
locus1_HPAG1_0775                696  GGTGGAGTTTAGGAAGTTGGGGGATATTGGGGAATTTTATGGCGGACTTGTTGGAAAAAG
locus1_HPG27_746                 741  GGTGGAGTTTAGGAAGTTGGGGGAGGTGTGTGAAATAATTAG------------------
locus1_HPP12_0797                678  GGTGGAGTTTAAAACGCTTGAAGAGGTTTTTGAAATTAGAAATGGTTACACCCCATCAAA
locus1_HPSH_02865                711  GGTGGAGTTTAGGAAATTGGGGGAGGTGTGTGAAATTTTAGA---------------TAA
locus1_HPF16_0572                681  GGTGGAGTTTAGGAAGTTGGGGGAGGTGTGTGAAATAATTAG------------------
locus1_HPF30_0541-HPF30_0542     636  G-TGGAGTTTAAAACGCTTGAAGAGGTTTTTGAAATTAAAAATGGTTACACCCCATCAAA
locus1_HPF32_0757                657  GGTGGAGTTTAGGAAGTTGGGGGAGGTGTGTGAAAGCACAAATAAAA-------AAACAC
locus1_HPF57_0810                723  GGTGGAGTTTAGGAAATTGGGGGAGGT-GCTAGAATATGATC---------------AAC
locus2_HP0848-HP0849             725  GGTGGAGTTTAGGAAATTGGGGGAGGTGTGTGAAAGCACAAATAAAA-------AAACAC
locus2_jhp0785                   45   GGTGGGGTTTAGGAAGTTGGGGGAGGTGTGTGAAAGCACCAATAAAA-------AAACAC
locus2_HPAG1_0832                672  GGTGGAGTTTAGGAAGTTGGGGGAGGTGTGTGAAATTTTAGATAATCGGCGTATCCCAAT
locus2_HPG27_805                 729  GGTGGAGTTTAGGAAGTTGGGGGAGGTGTGTGAAATAATTAG------------------
locus2_HPP12_0849                534  ------------------------------------------------------------
locus2_HPSH_02565                723  GGTGGAGTTTAGGAAATTGGGGGAGGTGTGTGAAATAATTAG------------------
locus2_HPF16_0513                723  GGTGGAGTTTAGGAAGTTGGGGGAGGTGTGTGAAATAATTAG------------------
locus2_HPF30_0484                678  GGTGGAGTTTAGGAAGTTGGGGGATAT-TGGGGAATTTTATAGCGGACTTGTTGGAAAAA
locus2_HPF32_0814                648  GGTGGAGTTTAGGAAGTTGGGGGAGGTGTGTGAAAGCACAAATAAAAAAACACTCAAAAT
locus2_HPF57_0869                654  GGTGGAGTTTAGGAAGTTGGGGGAGGTGTGTGATTTTCAAAAAGGAAAATCAATAACAAA

locus1_HP0790                    774  TCGGCGTATCCCAATTGCGAAA-AATAAAAGAAATCCG------------GGAATTT---
locus1_jhp0726                   800  TTTTGAAAAGTGATTTACAAGACAAGGGGAGACCTGTTGT---TC---ATTATGGACAAA
locus1_HPAG1_0775                756  TAAAAAATCTTTCTCTCAAGGGAATAA----ATTTTATGTTCCTTATATCAATGTTTTTA
locus1_HPG27_746                 782  ---AGGTAAAAGGGTTACAAAAAAAGAAATACTAGATAA---------AGGAAAATATC--
locus1_HPP12_0797                738  AAACAATCCTGAATTTTGGAAAAATGGGACTATCCCTTGGTTTAGAATGGAAGACCTTAG
locus1_HPSH_02865                756  TCGGCGTATCCCAATTGCGAAA-AATAAAAGAAATCCG------------GGAATTT---
locus1_HPF16_0572                722  ---AGGTAAAAGGGTTACAAAAAAAGAAATATTAGATAA---------AGGAAAATATCCC
locus1_HPF30_0541-HPF30_0542     695  AAACAATCCTGAATTTTGGGAAAAAGGGACTATCCCTTGGTTTAGAATGGATGACATTAG
locus1_HPF32_0757                710  TCAAAATAAGCGAAGTG--AGCGAAGTAAAAA----------------ATAAGAGA----
locus1_HPF57_0810                767  CCAATAAATATTGCGTAACGAGTAAAGAATTTGATA--------------AAAGTT----
locus2_HP0848-HP0849             778  TCAAAATAAGCGAAGTA--AGTGAAGTAAAAA----------------ATAAGGGA----
locus2_jhp0785                   98   TTAAAATAAGTGAAGTG--AGCGAAGTAAAAA----------------ATAAGGGA----
locus2_HPAG1_0832                732  TGCGAAAAATAAA---AGAAAACCGGGAATTTATCCTTATTATGGAGCAAATGGAATTCA
locus2_HPG27_805                 770  ---AGGTAAAAGGGTTACAAAAAAAGAAATACTAGATAA---------AGGAAAATATC--
locus2_HPP12_0849                534  ------------------------------------------------------------
locus2_HPSH_02565                764  ---GGGTAAAAGGGTTACAAAAAAAGAAATATTAGATAA---------AGGAAAATATC--
locus2_HPF16_0513                764  ---AGGTAAAAGGGTTACAAAAAAAGAAATATTAGATAA---------AGGAAAATATC--
locus2_HPF30_0484                737  GTAAAAAATCTTTCTCTCAAGGGAATAAATTTTATGTTCC---TTATGTCAATGTTTTTA
locus2_HPF32_0814                708  AAGC------GAAGTGAGCGAAGTAAAAAATAAG---------AGAATGTATCCAGTGAT
locus2_HPF57_0869                714  AAA-AGCCGTAACCTTTGGAAAAGT-------CCCTGTTATTTCGGGGGAAGAC------
```

```
locus1_HP0790                   817  ---ATCCTTATTATGGAGCAAATGGAA-TTCAAGATTATATTGATAGCTATATTTTTGAT
locus1_jhp0726                  854  TACACACTCAATACAATCTTTCAATTG-ATAAGACAATTTCATATGTTAATGACG-----
locus1_HPAG1_0775               812  ATAATCCACAACTAGATCTAAATGCTTTAGAAAGCGT-TCAAATAGGAGATAAAGAAAAA
locus1_HPG27_746                829  ---CCGTTGTATCTGGTGGAATAGGAT-TTATGGGATATTTAAATGAATATAACAGAGAG
locus1_HPP12_0797               798  AGAAAATGGGAGGATTTTAAAAGACTCTATCCAACACATTACCCCAAAGGCTTTAAAGGG
locus1_HPSH_02865               799  ---ATCCTTATTATGGAGCAAATGGAA-TTCAAGATTATATTGATAGCTATATTTTTGAT
locus1_HPF16_0572               772  GTTGTATCTGGTGGAAT-----AGGAT-TTATGGGATATTTAAATGAATATAACAGAGAG
locus1_HPF30_0541-HPF30_0542    755  AGAAAATGGGAGGATTTTAAAAGACTCTATCCAACACATTACCCCAAAGGCTTTAAAGGG
locus1_HPF32_0757               747  -ATGTATCCAGTGATA------AATTC-AGGGAGGGATTT-GTATGGTTATTAC------
locus1_HPF57_0810               808  ---ATCCTACTCCCGTTTTAACCGCAG-GGAAAACCTTTATTTTAGGTTATACAAACGAA
locus2_HP0848-HP0849            815  -ATGTATCCAGTGATA------AATTC-AGGGAGGGATTT-GTATGGTTATTAC------
locus2_jhp0785                  135  -ATGTATCCAGTGATA------AATTC-AGGGAGGGAATT-GTATGGTTATTAC------
locus2_HPAG1_0832               789  AGATTATATTGATAGCT---ATATTTTTGATGGGGATTTTGTGCTAG---TTGGTGAAGA
locus2_HPG27_805                817  ---CCGTTGTATCTGGTGGAATAGGAT-TTATGGGATATTTAAATGAATATAACAGAGAG
locus2_HPP12_0849               534  ------------------------------------------------------------
locus2_HPSH_02565               811  ---CCGTTGTATCTGGTGGAATAGGAT-TTATGGGATATTTAAATGAATATAACAGAGAG
locus2_HPF16_0513               811  ---CCGTTGTATCTGGTGGAATAGGAT-TTATGGGATATTTAAATGAATATAACAGAGAG
locus2_HPF30_0484               794  ATAATCCACAATTAGATTTAAATGCTT-TAGAAAGCGTTCAAATAGGGGATAAAGAAAAA
locus2_HPF32_0814               753  AAATTCAGGGAGGGATTTGTATGGTT-----------ATTACCATGA----TTTTAACAA
locus2_HPF57_0869               760  ----AACCCGCTTATTAT------CACAATGAGGCAAATCGTAGCGGAG-----AAACAA

locus1_HP0790                   874  GGGGATTTTGTGCTAGTTGGTGAAGA-----TGGGAGCGTTATC----AATAAGGATAAT
locus1_jhp0726                  908  CTTTATTCCATAAATTAAAAAAAGCAAAGCCAAACGATATTCTCA---TCGCCACAACCT
locus1_HPAG1_0775               871  CAAAATACAATTCAATTAGGCGATGTGCTTTTTACCGGTTCGTCTGAAAATTTAGA--GG
locus1_HPG27_746                886  GAAAATACAATTACTATAGC----------------------TC----AATATGGA--AC
locus1_HPP12_0797               858  TAAGAAATTATTCCCTAAAA--ATTCTATTATTATTTCTACAACAGCAACGATAGG--AG
locus1_HPSH_02865               856  GGGGATTTTGTGCTAGTTGGTGAAGA-----TGGGAGCGTTATC----AATAAGGATAAT
locus1_HPF16_0572               826  GAAAATACAATTACTATAGC----------------------TC----AATATGGA--AC
locus1_HPF30_0541-HPF30_0542    815  TAAGAAATTATTCCCTAAAA--ATTCTATTATTATTTCTACAACAGCAACCATAGG--CG
locus1_HPF32_0757               793  CATGATTTTAACAATGATGGAGA---AAATATAACTATTGCATCT---AGGGGAGAATAT
locus1_HPF57_0810               865  AAAGACAATATTTA-TCAAGCGAGTAAAAGCTCTCCGGTT-ATC----ATTTTTGATGAT
locus2_HP0848-HP0849            861  CATGATTTTAACAATGATGGAGA---AAATATAACTATTGCATCT---AGGGGAGAATAT
locus2_jhp0785                  181  CATGATTTTAATAATGATGGAGA---AAATATAACTATTGCATCT---AGAGGAGAATAT
locus2_HPAG1_0832               843  TGGGAGCGTTATCAATAAGA--ATAATACTCCTGTTGTGAATTGGGCAAGTGGAAA----
locus2_HPG27_805                874  GAAAATACAATTACTATAGC----------------------TC----AATATGGA--AC
locus2_HPP12_0849               534  ------------------------------------------------------------
locus2_HPSH_02565               868  GAAAATACAATCACTATAGC----------------------TC----AATATGGA--AC
locus2_HPF16_0513               868  GAAAATACAATTACTATAGC----------------------TC----AATATGGA--AC
locus2_HPF30_0484               853  CAAAATACAATTCAATTAGGCGATGTGCTTTTTACTGGTTCATCTGAAAATTTAGAGGAT
locus2_HPF32_0814               798  TGATGGA---------GAAA--ATATAACTATTGCATCTAGGGGAGAATATGCAGGA-TT
locus2_HPF57_0869               806  TAGCGATTTCTTCATCTGGA--GTG----TATGCTGGCTATGTTAGTTAT--TGGG--AT

locus1_HP0790                   925  ACTCCTGT---------TGTGAATTGGGCAAGCGGAAAA-------ATATGGGTGAA--C
locus1_jhp0726                  965  CAGAAAT---------GTAAAAGATGTAGGAAAGAGTAT---TGCATGGTTAGGAAATGA
locus1_HPAG1_0775               929  ATTGTGCGA------TGTCTTGCGTGGTTACTCAAAAGA-----TTGAAAAAGATATTTA
locus1_HPG27_746                918  CGCCGGAT--------TTGTCAATTGGCAAAATCAAAAG-------TTTTGGGCAAA--T
locus1_HPP12_0797               914  AGCATGC--------CCTTTTAATCGTTGATTCGTTAG-------CGAATCAACAATTCA
locus1_HPSH_02865               907  ACTCCTGT---------TGTGAATTGGGCAAGCGGAAAA-------ATATGGGTGAA--C
locus1_HPF16_0572               858  TGCCGGAT--------TTGTCAATTGGCAAAATCAAAAG-------TTTTGGGCAAATGA
locus1_HPF30_0541-HPF30_0542    871  AACATGC--------CCTTTTAATCGTTGATTCGTTAG-------CGAATCAACGATTCA
locus1_HPF32_0757               846  -GCAGGAT--------TTATAAACTACTTCAATGAAAAA---TTTTTTGCAGGGGGTCTA
locus1_HPF57_0810               919  TTCACAAC---------AGCAACCCAATGGGTTGATT----TCCCTTTCAAAGTAAAATC
locus2_HP0848-HP0849            914  -GCAGGAT--------TTATAAACTATTTCAATGAAAAA---TTTTTTGCAGGGGGTCTA
locus2_jhp0785                  234  -GCAGGAT--------TTATAAATTACTTTAATGAAAAA---TTTTTTGCAGGGGGTCTA
locus2_HPAG1_0832               897  AATATGG--------GTGAATAATCAT---------------------------GCTCAT
locus2_HPG27_805                906  CGCCGGAT--------TTGTCAATTGGCAAAATCAAAAG-------TTTTGGGCAAA--T
locus2_HPP12_0849               534  ------------------------------------------------------------
locus2_HPSH_02565               900  TGCCGGAT--------TTGTCAATTGGCAAAATCAAAAG-------TTTTGGGCAAA--T
locus2_HPF16_0513               900  TGCCGGAT--------TTGTCAATTGGCAAAATCAAAAG-------TTTTGGGCAAA--T
locus2_HPF30_0484               913  TGTGCGATGTCTTGCGTAGTTACTCAAAAAATTGAAAAAGATATTTATCTTAATAGTTTT
locus2_HPF32_0814               846  TATAAAC--------TACTTCAATGAAAAATTTTTTGC-------AGGGGGTCTATGTTA
locus2_HPF57_0869               856  AT--TCC--------TGTTTTTCTTGCTGACTC-----------------------TTTT
```

```
locus1_HP0790                   967  AATCAT--GCTCATGTGCTTCAAACAAAAAATGAACTAAAA---TTAAAGTTTTTATATT
locus1_jhp0726                  1014 AGAAGTTGCCTTTTCGGGTGAAATGTATAGCTATTCTACAA---ATGAAAACCCTAAATT
locus1_HPAG1_0775               978  TCTTAATAGTTTTTGTTTTGGTTTTAGATTTTTTGATAAGAATTTATTTAATCCATCGTT
locus1_HPG27_746                961  GATGTT--TGTTTTTCTCTCATTCCAAAAGAAACCCTAATC---AATAGATACCTTTATT
locus1_HPP12_0797               959  CTTTTTTAAGCAAAAAAGCGAATTGTGATCTTGCTTTAGA----CATGAAATTCTTTTTT
locus1_HPSH_02865               949  AATCAT--GCTCATGTGCTTCAAACAAAAAATGAATTAAAA---TTAAAGTTTTTGTATT
locus1_HPF16_0572               903  TGTTTG----TTTTTCTGTCATTCCAAAAGAAACCCTAATC---AATAGATACCTTTATT
locus1_HPF30_0541-HPF30_0542    916  CTTTTTTAAGCAAAAAAGCGAATTGTAATATTGCTTTAGA----CATGAAATTCTTTTTT
locus1_HPF32_0757               895  TGTTAT--CCCTATAAAGTTAAAGATACTAACGAGCTTTTA---ACAAAATTTTTATACT
locus1_HPF57_0810               966  AAGCGC--TATGAAAATCTTACTCCCAAAAAATCCTACAAT---TAACATCAGATTTATC
locus2_HP0848-HP0849            963  TGTTAT--CCCTATAAAGTTAAAGACACTAACGAGCTTTTA---ACAAAATTTTTATACT
locus2_jhp0785                  283  TGTTAT--CCCTATAAAGTTAAAGATACTAACGAGCTTTTA---ACAAAATTTTTATACT
locus2_HPAG1_0832               922  GTGCTTCAAACAAAAAA--------------TGAACTAAA----ATTAAAGTTTTT----
locus2_HPG27_805                949  GATGTT--TGTTTTTCTCTCATTCCAAAAGAAACCCTAATC---AATAGATACCTTTATT
locus2_HPP12_0849               534  ------------------------------------------------------------
locus2_HPSH_02565               943  GATGTT--TGTTTTTCTGTCATTCCAAAAGAAACCCTAATC---AATAGATACCTTTATT
locus2_HPF16_0513               943  GATGTT--TGTTTTTCTGTCATTCCAAAAGAAACCCTAATC---AATAGATACCTTTATT
locus2_HPF30_0484               973  TGTTTT--GGTTTTAGATTTTTTGATGAGAATTTATTTAAT---CCATCGTTTCTTAAGC
locus2_HPF32_0814               891  TCCCTATAAAGTTAAA----GATACTAACGAGCTTTTAAC----AAAATTTTTATACTTT
locus2_HPF57_0869               883  TCTGTTTCACCAAAACAAAAAACCCTAATGCC----TAAA----TATCTTTTTCATTATC

locus1_HP0790                   1022 T--------TTATTTACAA------------ACGATAGATGTTAGCTATTGTGT-TGCTG
locus1_jhp0726                  1071 TATCATTTATTACTTTCAAACTTATTTTTTTCAAAAAGAAAAAGAAAAGAAAATAACAGG
locus1_HPAG1_0775               1038 TCTTAAACATTTTTTAAGAGACTACAATTTTAGGAAAAATATTTCAAAAGTTGC-TAATG
locus1_HPG27_746                1016 A--------TGTATTAACAAAC----ATGCAAAATTATTTATATTCTATTTCAAATAGAA
locus1_HPP12_0797               1015 TACCAATGCTTTCTTTTAGGGGAATGGT----GTAAAAATAATATTAATGTTTC-A---G
locus1_HPSH_02865               1004 T--------TTATTTACAA------------ACGATAGATGTTAGCTATTGTGT-TGCTG
locus1_HPF16_0572               956  A--------TGTATTAACAAAC----ATGCAAAATTATTTATATTCTATTTCAAATAGAA
locus1_HPF30_0541-HPF30_0542    972  TATCAATGTTTTCTTTTGGGGGAATGGT----GCAAAAAAAATACTAATGTTTC-A---G
locus1_HPF32_0757               950  T--------TTATCTCAAAACTAATGAAATCCAAATT---ATGGAGAACCTTGTTTCTCG
locus1_HPF57_0810               1021 T--------TTTTTTA--------------TATGCAAACTATTCCCTATAATAT-CAGTG
locus2_HP0848-HP0849            1018 T--------TTATCTCAAAACTAATGAAATCCAAATT---ATGGAGAACCTTGTTTTTCG
locus2_jhp0785                  338  T--------TTATCTCAAAACCAATGAAACCCAAATT---ATGGAAAATCTTGTTTTTCG
locus2_HPAG1_0832               959  -----GTATTTTTATTTACAAACAATAG----ATGTTAGCTATTATGTTGCTGG-A---A
locus2_HPG27_805                1004 A--------TGTATTAACAAAC----ATGCAAAATTATTTATATTCTATTTCAAATAGAA
locus2_HPP12_0849               534  ------------------------------------------------------------
locus2_HPSH_02565               998  A--------TGTATTGACAAAC----ATGCAAAATTATTTATATTCTATTTCAAATAGAA
locus2_HPF16_0513               998  A--------TGTATTAACAAAC----ATGCAAAATTATTTATATTCTATTTCAAATAGAA
locus2_HPF30_0484               1028 A--------TTTTTTAAGAGACTACAATTTTAGGAAAAATATTTCAAAAGTTGC-TAATG
locus2_HPF32_0814               943  TATCTCAAAACTAATGAAATCCAAATTA----TGGAGAACCTTGTTTCTCGTGG-C---A
locus2_HPF57_0869               935  TTACAACACAACAAGAT--GCGATCCAT----GCAACAAAAAGCACAGGG---------G

locus1_HP0790                   1061 GAACTCCGC----CAAAAATCAACCAAGAAAATTTAAAAAAAATAGCAATCCCCATCCCA
locus1_jhp0726                  1131 AACTAAAGTTA--TGCGTATCCATGAAAATGATTTAAAACAAATAACAATCCCCATCCCA
locus1_HPAG1_0775               1097 GTGTAACACGCTTCAATGTTTCAAAGCAATTACTTTCACAAATA-ACCATCCCCATCCCA
locus1_HPG27_746                1064 GCGCTATACCTTATAGCATTTC-TAGTAATAACATTATGCAAATAACAATCCCCATCCCA
locus1_HPP12_0797               1067 GTTT----TGCTTCTGTGGAT---ATGAGTGCTTTTAAAAAATATAAGTTCCCCATCCCA
locus1_HPSH_02865               1043 GAACTCCGC----CAAAAATCAACCAAGAAAATTTAAAACAAATAACAATCCCCATCCCA
locus1_HPF16_0572               1004 GTGCTATACCTTATAGCATTTC-TAGTAATAACATTATGCAAATCACAATCCCCATCCCA
locus1_HPF30_0541-HPF30_0542    1024 GTTT----TGCTTCTGTGGAT---ATGACCGCTTTTAAAAAATATAAGTTCCCCATCCCA
locus1_HPF32_0757               999  TGGCAGTATCC--CCGCACTCAATAAAGCAGATATTGAAACTTTAACAATCCCCATCCCA
locus1_HPF57_0810               1058 GGG-AACATA---CAAGGCAGTGGATTTCTCGCTATTCAAAAATAACAATCCCCATCCCA
locus2_HP0848-HP0849            1067 TGGCAGTATCC--CCGCACTCAATAAAGCAGATATTGAAACTTTAACAATCCCCATCCCA
locus2_jhp0785                  387  TGGTAGTATCC--CCGCACTCAATAAAGCAGATATTGAAACTTTAACCATCCCCATCCCA
locus2_HPAG1_0832               1007 CTCC----GCCAAAAATCAAC---CAAGAAAATTTAAAAAAAATAACCATCCCCATCCCA
locus2_HPG27_805                1052 GCGCTATACCTTATAGCATTTC-TAGTAATAACATTATGCAAATAACAATCCCCATCCCA
locus2_HPP12_0849               534  ------------------------------------------------------------
locus2_HPSH_02565               1046 GCGCTATACCTTATAGCATTTC-TAGTAACAACATTATGCAAATAACAATCCCCATCCCA
locus2_HPF16_0513               1046 GTGCTATACCTTATAGCATTTC-TAGTAATAACATTATGCAAATCACAATCCCCATCCCA
locus2_HPF30_0484               1079 GCGTAACACGTTTCAATGTTTCAAAGCAATTACT-TTCAAAAATAACAATCCCCATCCCA
locus2_HPF32_0814               995  GTAT----CCCCGCACTCAAT---AAAGCAGATATTGAAACTTTAACAATCCCCATCCCA
locus2_HPF57_0869               980  GAAT----TCCTCATGTTTAT---AGCAAAGACTTACAAAATTTTTTAATCCCCATCCCA
```

```
                                  repeat y                                     conserved region
locus1_HP0790                1117 CCCCTAGAGATCCAACAAGAGATCGTTAAGATTTTGGATCAATTTTCAATTTTAACCACC
locus1_jhp0726               1189 CCCCTAGAGATCCAACAAGAGATCGTTACGATTTTGGATCAATTTTCAGCCCTAACTACT
locus1_HPAG1_0775            1156 CCCCTAGAGATCCAACAAGAGATCGTTAAGATTTTGGATCAGTTTTCAGCCCTAACCACC
locus1_HPG27_746             1123 CCCCTAGAGATCCAACAAGAGATCGTTAAGATTTTGGATCAATTTTCACTTTTAACCACC
locus1_HPP12_0797            1120 CCCCTAGAGATCCAGCAAGAGATCGTTAAGATTTTGGATCAATTTTCACTTTTAACCACC
locus1_HPSH_02865            1099 CCCCTAGAAATCCAACAAGAGATCGTTAAGATTTTGGATCAATTCTTGGCCCTAACCACC
locus1_HPF16_0572            1063 CCCCTAGAAATCCAACAAGAGATCGTTAAGATTTTGGATCAATTTTCAATCCTAACCACC
locus1_HPF30_0541-HPF30_0542 1077 CCCCTAGAAATCCAACAAGAGATCGTTAAGATTTTGGATCAATTTTCAACCCTAACCACC
locus1_HPF32_0757            1057 CCCCTAGAAATCCAGCAAGAGATCGTTAAGATTTTGGATCAATTTTTGGCTCTAACTACC
locus1_HPF57_0810            1114 CCCCTAGAAATCCAACAAGAGATCGTTAAGATTTTGGATCAATTTTCAATCCTAACCACC
locus2_HP0848-HP0849         1125 CCTCTAGAGATCCAACAAGAGATCGTTAAGATTTTGGATCAATTTTCAGCCCTAACCACC
locus2_jhp0785               445  CCCCTAGAGATCCAACAAGAGATCGTTACGATTTTGGATCAATTTTCAGCCCTAACTACT
locus2_HPAG1_0832            1060 CCCCTAGAGATCCAGCAAGAGATCGTTAAGATTTTGGATCAATTTTCAGCCCTAACCACC
locus2_HPG27_805             1111 CCCCTAGAGATCCAACAAGAGATCGTTAAGATTTTGGATCAATTTTCACTTTTAACCACC
locus2_HPP12_0849            534  ------------------------------------------------------------
locus2_HPSH_02565            1105 CCCCTAGAAATCCAACAAGAGATCGTTAAGATTTTGGATCAATTCTTGGCCCTAACCACC
locus2_HPF16_0513            1105 CCCCTAGAAATCCAACAAGAGATCGTTAAGATTTTGGATCAATTTTCAATCCTAACCACC
locus2_HPF30_0484            1138 CCCCTAGAAATCCAACAAGAGATCGTTAAGATTTTGGATCAATTTTCAACCCTAACCACC
locus2_HPF32_0814            1048 CCCCTAGAAATCCAACAAGAGATCGTTAAGATTTTGGATCAATTTTCAATCCTAACTACC
locus2_HPF57_0869            1033 CCCCTAGAAATCCAACAAGAGATCGTTAAGATTTTGGATCAATTTTTGGCTCTAACCACC

locus1_HP0790                1177 GATTTATTAGCCGGTATCCCCGCTGAAATAAAAGCGAGAAAAAAGCAATACGAATATTAC
locus1_jhp0726               1249 GATTTGCAAGCCGGTATCCCTGCTGAAATCAAAGCCAGGAAAAAACAATACGAATATTAC
locus1_HPAG1_0775            1216 GATTTATTAGCCGGTATCCCCGCTGAAATAAAAGCCCGAAAAAAGCAATACGAATATTAC
locus1_HPG27_746             1183 GATTTATTAGCCGGTATCCCCGCTGAAATAAAAGCCCGAAAAAAACAATACGAATACTAC
locus1_HPP12_0797            1180 GATTTATTAGCCGGTATCCCCGCTGAAATTAAAGCCCGAAAAAAGCAATACGAATATTAC
locus1_HPSH_02865            1159 GATTTATTAGCCGGTATCCCGGCTGAAATAGAAGCCAGGAAAAAGCAATACCAATATTAC
locus1_HPF16_0572            1123 GATTTATTAGCCGGTATCCCGGCTGAAATAGAAGCCAGGAAAAAGCAATACGAATATTAC
locus1_HPF30_0541-HPF30_0542 1137 GATTTATTAGCCGGTATCCCGGCTGAAATAGAAGCCAGGAAAAAGCAATACGAATATTAC
locus1_HPF32_0757            1117 GATTTATTAGCCGGTATCCCAGCTGAAATAGAAGCCAGGAAAAAGCAATACGAATATTAT
locus1_HPF57_0810            1174 GATTTATTAGCCGGTATCCCAGCTGAAATAGAAGCCAGGAAAAAGCAATACGAATATTAC
locus2_HP0848-HP0849         1185 GATTTATTAGCCGGTATCCCCGCTGAAATAAAAGCCCGAAAAAAGCAATACGAATATTAC
locus2_jhp0785               505  GATTTACAAGCCGGTATCCCCGCTGAAATCAAAGCCAGGAAAAAGCAATACGAATATTAC
locus2_HPAG1_0832            1120 GATTTATTAGCCGGTATCCCCGCTGAAATAAAAGCCCGAAAAAAGCAATACGAATATTAC
locus2_HPG27_805             1171 GATTTATTAGCCGGTATCCCCGCTGAAATAAAAGCCCGAAAAAAACAATACGAATACTAC
locus2_HPP12_0849            534  ------------------------------------------------------------
locus2_HPSH_02565            1165 GATTTATTAGCCGGTATCCCGGCTGAAATAGAAGCGAGAAAAAAGCAATACGAATATTAC
locus2_HPF16_0513            1165 GATTTATTAGCCGGTATCCCGGCTGAAATAGAAGCCAGGAAAAAGCAATACGAATATTAC
locus2_HPF30_0484            1198 GATTTATTAGCCGGTATCCCGGCTGAAATAGAAGCCAGGAAAAAGCAATACGAATATTAC
locus2_HPF32_0814            1108 GATTTATTAGCCGGTATCCCAGCTGAAATAGAAGCCAGGAAAAAGCAATACGAATATTAT
locus2_HPF57_0869            1093 GATTTATTAGCCGGTATCCCCGCTGAAATAGAAGCCAGGAAAAAGCAATACGAATATTAC

locus1_HP0790                1237 CGAGAAAAATTATTGACCTTCAAGCCCCTAACCCCACTAAATAGCAAAGAGCTTGCGTAA
locus1_jhp0726               1309 CGAGAAAAATTACTGACCTTCAAACCCCTAACCCTCA------ATAAAGAAGTTAAAAAA
locus1_HPAG1_0775            1276 CGAGAAAAACTATTGACTTTCAAACCTCTAACCCCCCA------CAAAGAAGTTAAAAAA
locus1_HPG27_746             1243 CGAGAAAAACTACTGACCTTCAAACCCCTAACCCCCATAAAGAAGTTAAAAAATGAGTTT
locus1_HPP12_0797            1240 CGAGAAAAACTACTGACCTTCAAACCCCTAACCCCAC-------TAAATAACAAAGAGCT
locus1_HPSH_02865            1219 AGAGAAAAACTACTGACCTTCAAACCACTAACCCCCC------ACAAAGAAGTTGAAAAA
locus1_HPF16_0572            1183 CGAGAAAAACTATTGGCATTCAAACCACTAACCCCCC------ACAAAGAAGTTAAAAAA
locus1_HPF30_0541-HPF30_0542 1197 AGAGAAAAACTATTGAGTTTCAAACCACTCAAGGCAT-------AA--------------
locus1_HPF32_0757            1177 AGAGAAAAATTATTGGCATTCAAACCACTAACCCCAC------ACAAAGAAGTTAAAAAA
locus1_HPF57_0810            1234 AGAGAAAAACTATTGACCTTCAAACCACTAACCCCAC------ACAAAGAAGTTAAAAAA
locus2_HP0848-HP0849         1245 CGAGAAAAACTACTGACCTTCAAACCTCT---CCAAA------ACAAGGAATAA------
locus2_jhp0785               565  CGAGAAAAATTACTGGCCTTCAAACCCCTAACCCCAC-------TAAATAACAAAGAGCT
locus2_HPAG1_0832            1180 CGAGAAAAATTACTGGCCTTCAAACCCCTAACCCCAC-------TAAATAACAAAGAGCT
locus2_HPG27_805             1231 CGAGAAAAACTACTGACCTTCAAACCCCTAACCCCACTAAATAACAAAGAGCTTGCGTAA
locus2_HPP12_0849            534  ------------------------------------------------------------
locus2_HPSH_02565            1225 AGAGAAAAACTACTGACTTTTAAACC----ACTCCAAAACAAGGCATAA-----------
locus2_HPF16_0513            1225 CGAGAAAAACTATTGGCATTCAAACC----ACTCCCAAACAAGGCATAA-----------
locus2_HPF30_0484            1258 AGAGAAAAACTATTGAGTTTCAAACCACTCAAGGCAT------AA---------------
locus2_HPF32_0814            1168 AGAGAAAAATTATTGGCATTCAAACCACTCAAGGCAT-------AA--------------
locus2_HPF57_0869            1153 CGAGAAAAACTATTGGCCTTCAAACCACTCAAGGCAT-------GA--------------
```

**locus1_HP0790**	1296	-------
**locus1_jhp0726**	1363	TGA----
**locus1_HPAG1_0775**	1330	TGCTAA-
**locus1_HPG27_746**	1303	TGA----
**locus1_HPP12_0797**	1293	TGCGTAA
**locus1_HPSH_02865**	1273	TGA----
**locus1_HPF16_0572**	1237	TGCTAA-
**locus1_HPF30_0541-HPF30_0542**	1235	-------
**locus1_HPF32_0757**	1231	TGC-TAA
**locus1_HPF57_0810**	1288	TGCTAA-
**locus2_HP0848-HP0849**	1289	-------
**locus2_jhp0785**	618	TGCGTAA
**locus2_HPAG1_0832**	1233	TGCGTAA
**locus2_HPG27_805**	1290	-------
**locus2_HPP12_0849**	534	-------
**locus2_HPSH_02565**	1269	-------
**locus2_HPF16_0513**	1269	-------
**locus2_HPF30_0484**	1296	-------
**locus2_HPF32_0814**	1206	-------
**locus2_HPF57_0869**	1191	-------
