# Supplementary material for: Domain Movement within a Gene: A Novel Evolutionary Mechanism for Protein Diversification
Source: PLoS One. 2011 Apr 14;6(4):e18819. doi: 10.1371/journal.pone.0018819 (PMC3077401; doi:10.1371/journal.pone.0018819)
Supplement: Figure S3 — Nucleotide sequence alignment of Group 3 S (See Fig. 3 ). (PDF) [file pone.0018819.s003.pdf]

```
                              conserved region                         repeat x'
locus3_jhp1422       1    GTGATGGAACGCATGGACGCTTTAATAACGCCCTCAAATTGGCAAAAAGTAAGGCTTGGG
locus3_HPAG1_1462    1    ------------------------ATGCTGCCCTTAAATTGGCAAAGAGTGAGACTTGGG
locus3_HPG27_1455    1    ------------ATGGACGCATTAACAACGCCC---------------------------
locus3_HPP12_1508    1    GTGACAGAACGCATGGACGCGTTAACAACGCCCTCAAATTGGCAAAGAGTGAGACTTGGG
locus3_HPSH_07885    1    ------------------------------------------------------------
locus3_HPF16_1428    1    ------------------------ATGCTGCCC---------------------------
locus3_HPF30_1405    1    ------------ATGGACGCATTAATGCTGCCC---------------------------
locus3_HPF32_1419    1    ------------ATGGACGCATTAATGCTGCCCTTAAATTGGCAAAGAGTAAGGCTTGGG
locus3_HPF57_1447    1    ------------------------ATGCTGCCC---------------------------
locus5_HPSH_07300    1    ------------------------ATGCTGCCCTTAAATTGGCAAAGAGTGAGGCTTGGG
locus5_HPF30_1299    1    ------------ATGCTCCCATTAATGCTGCCCTTAAATTGGCAAAGAGTAAGGCTTGGG

                                TRD1
locus3_jhp1422       61   GATATATTAGAACTTCTAACCGACTACCATGCAAATGGATCTT-ATGAGATACTTAAAAA
locus3_HPAG1_1462    37   GATAT--TGGCAA------------ACCATGCATGTGTAAAAGAGTTATGAAACATCAAA
locus3_HPG27_1455    21   ------------------------------------------------------------
locus3_HPP12_1508    61   GATATGACAACAAGCTT-------TACCAAACAAACAGGCTTTGATTATTCTGCTTCTAT
locus3_HPSH_07885    1    --------------------------GTGATAGAACGCATGGACGCATTAATGTTGCCCT
locus3_HPF16_1428    9    ------------------------------------------------------------
locus3_HPF30_1405    21   ------------------------------------------------------------
locus3_HPF32_1419    49   GATAT--TGGCAA------------ACCATGCATGTGTAAAAGAGTGATGAAGCATCAAA
locus3_HPF57_1447    9    ------------------------------------------------------------
locus5_HPSH_07300    37   GATATA--GGCAA------------ACCATGCATGTGTAAAAGAGTGATGAAGCATCAAA
locus5_HPF30_1299    49   GATATGACAACAAGCTT-------TACCAAACAAACAGGCTTTGATTATTCTGCTTTTAT

locus3_jhp1422       120  TAATGTAACATT-ATTAAAAAATGTAGATTTTGCTAT------TATGATAAGAACTACTA
locus3_HPAG1_1462    83   CAACACGATAT-----GGTGAAATTCCATTTTATAAA-------ATAGGCACATTTGGCA
locus3_HPG27_1455    21   ------------------------------------------------------------
locus3_HPP12_1508    114  TAAACCAACACTCATAAAAGAACAACTGCCTAACTATATTCCATTTATTCAAAATAAGGA
locus3_HPSH_07885    35   TAAATTGGCAAAGAGTGAGGCTTGGGGATATATTTTTCATTA--CGGCTGGAGGCGATTT
locus3_HPF16_1428    9    ------------------------------------------------------------
locus3_HPF30_1405    21   ------------------------------------------------------------
locus3_HPF32_1419    95   CAACACGATAT-----GGTGAAATTCCATTCTATAAA-------ATAGGCACATTTGGCA
locus3_HPF57_1447    9    ------------------------------------------------------------
locus5_HPSH_07300    83   CAACACGATAT-----GGTGAAATTCCATTCTATAAA-------ATAGGCACATTTGGCA
locus5_HPF30_1299    102  TAAGCCGACACTCATAAAAGAATATCTGCCTAACTATATTCCATTTATTCAAAATAAGGA

locus3_jhp1422       173  ATTTTGAAAACA----ATGATTTTAAAAATGATTTAATTTATATTGATAAAAAAGCCTAT
locus3_HPAG1_1462    131  ACACCGCTGATGCC---TTTATTTCAAAAAAGCTATTTTTA----GAATACAAAACAAAA
locus3_HPG27_1455    21   ------------------------------------------------------------
locus3_HPP12_1508    174  TTTTTTAGGACATTATATTAATTACAAAACAGATTATTTTATTCCTAATGAAATTGCAAT
locus3_HPSH_07885    93   ATCTAAGCCACATTATTCAAATACAAAACAAAGTGATTTTAATTACCCCATTTATTCAAA
locus3_HPF16_1428    9    ------------------------------------------------------------
locus3_HPF30_1405    21   ------------------------------------------------------------
locus3_HPF32_1419    143  ACACCGCTGATGCC---TTTATTTCAAAAAAGCTATTTTTA----GAATACAAAACAAAA
locus3_HPF57_1447    9    ------------------------------------------------------------
locus5_HPSH_07300    131  ACACCGCTGATGCC---TTTATTTCAAAAAAGCTATTTTTA----GAATACAAAACAAAA
locus5_HPF30_1299    162  TTTTTTAGGACACTATATTAATTATAAAACGGATTATTTTATTCCCAATGAAATTGCAAT

locus3_jhp1422       229  GAGTTTTTATCAAAGTCAAAAGTTTTTGCTGGAGATATTTTAGTTAATAAAATTGCCAAT
locus3_HPAG1_1462    184  TATTCTTTTCCAAA------------AAAAGGCGATATTTTA---ATTTCTGCTTCCGGA
locus3_HPG27_1455    21   ------------------------------------------------------------
locus3_HPP12_1508    234  TAGATTTCCACAAATTTTA-----TTGAATGAAAAATGCTTACTTATTTCAATCTCTGGG
locus3_HPSH_07885    153  TGCGATTGAAAAAAAAGGTTTATGTGGATATTCATCTTTTTTTATTATTAAAAATAAGAG
locus3_HPF16_1428    9    ------------------------------------------------------------
locus3_HPF30_1405    21   ------------------------------------------------------------
locus3_HPF32_1419    196  TATTCTTTTCCAAA------------AAAAGGCGATATTTTA---ATTTCTGCTTCCGGA
locus3_HPF57_1447    9    ------------------------------------------------------------
locus5_HPSH_07300    184  TATTCTTTTCCAAA------------AAAAGGCGATATTTTA---ATTTCTGCTTCCGGA
locus5_HPF30_1299    222  TAGGTTTCCACAAATTTTA-----TTGAATGAAAAATGCTTGCTTATTTCAATCTCTGGG
```

```
locus3_jhp1422     289  GCT----GGAACAGCATATTTTATGCCTAAATTAAATCAGCCTGTTTCTTTAGGTATGAA
locus3_HPAG1_1462  229  ACTATTGGTAAGGCAGTCATTTATGATGGAA---AACCCGCTTATTTTCAAGACTCAAAT
locus3_HPG27_1455  21   ------------------------------------------------------------
locus3_HPP12_1508  289  GCTATTGGGAATGTAGCAGTTTTTAACCACTCTCAAGATGCTT-TTATTGGTGGTGCTAT
locus3_HPSH_07885  213  CATAACAATAACGGCTAGAGGAACAATAGGTGTTGCTTTTTTTAGAGACTATCCTTATGT
locus3_HPF16_1428  9    ------------------------------------------------------------
locus3_HPF30_1405  21   ------------------------------------------------------------
locus3_HPF32_1419  241  ACTATTGGTAGGGCAGTCATTTATGACGGAA---AACCCGCTTATTTTCAAGACTCAAAT
locus3_HPF57_1447  9    ------------------------------------------------------------
locus5_HPSH_07300  229  ACTATTGGTAGGGCAGTCATTTATGACGGAA---AACCCGCTTATTTTCAAGACTCAAAT
locus5_HPF30_1299  277  GCTATTGGGAATGTAGCAGTTTTTAACCACTCTCAAGATGCTT-TTACTGGTGGTGCTAT

locus3_jhp1422     345  TTTATTTTTATTACGAATAAAACCAAGTTATAACAATCTTTTCAT---CTTTAAGCAAAT
locus3_HPAG1_1462  286  ATTGTTTGGATTGACAACGATGAAACATTAGTAAAAAATGATTTTTTATTTTATGCTTAT
locus3_HPG27_1455  21   ------------------------------------------------------------
locus3_HPP12_1508  348  TGCGGTATTAAAATTTAAAGAAAAAAAGTCGCTTGATTTTGTCATGCACTTTTTAATGTC
locus3_HPSH_07885  273  CCCTATCGGTAGACTTTTAGTCTTACAGCCAAAAATTAGTAATATTGATTGTAGGTTTTA
locus3_HPF16_1428  9    ------------------------------------------------------------
locus3_HPF30_1405  21   ------------------------------------------------------------
locus3_HPF32_1419  298  ATCGTTTGGATTGACAACGATGAAACATTAGTAAAAAATGATTTTTTATTTTATGCTTAT
locus3_HPF57_1447  9    ------------------------------------------------------------
locus5_HPSH_07300  286  ATCGTTTGGATTGACAACGATGAAACATTAGTAAAAAATGATTTTTTATTTTATGCTTAT
locus5_HPF30_1299  336  TGCAGTATTAAAATTTAAAGAAAAAAAGTCGCTTGATTTTGTCATGCACTTTTTAATGTC

locus3_jhp1422     402  AGCAAATTACGAAAGGGTTTTAAAAACTTTTGCTA--A-TGGTTCAGCAACAAAAACAAT
locus3_HPAG1_1462  346  T-CTAATGTTAAATGGAATACAGAGCATACAACTA--TCTTAAGACTTTATAATGA----
locus3_HPG27_1455  21   ------------------------------------------------------------
locus3_HPP12_1508  408  AGCTAGTGGGCAAAAATCATTATTGAATATCGTTA--AATCAAGCTCTCATAAAAATCTT
locus3_HPSH_07885  333  TGCGGAATATATCAATTCAAAAGTAAAATTTAACACCGAGCAAACTACTATTCCACAATT
locus3_HPF16_1428  9    ------------------------------------------------------------
locus3_HPF30_1405  21   ------------------------------------------------------------
locus3_HPF32_1419  358  T-CTAATGTTAAATGGAATACAGAGCATACAACTA--TCTTAAGACTTTATAATGA----
locus3_HPF57_1447  9    ------------------------------------------------------------
locus5_HPSH_07300  346  T-CCAATGTTAAATGGAATACAGAGCATACAACTA--TCTTAAGACTTTATAATGA----
locus5_HPF30_1299  396  AGCTAGTGGGCAAAAATTATTATTGAATGGCGTTA--AATCAAGCTCTCATAAAAATCTT
                                                           repeat y'
locus3_jhp1422     459  AACAAAAAATGTTATTAAAAATTTACTAATCCCCCTACCCCCCCTAAACGAACAAATCGC
locus3_HPAG1_1462  398  ---------TAATTTTAGAAATACTTTAATCCCCCTACCCCCTCTAAACGAACAAATCGC
locus3_HPG27_1455  21   ------------------------------------------------------------
locus3_HPP12_1508  466  A-CTATAGCTGACTTAAGAGACTTACTAATCCCCCTACCCCCCCTAAACGAACAAATCGC
locus3_HPSH_07885  393  AACAATCCCAAAAGTTGCACTATGTGAAATCCCCCTACCCCCTCTAAACGAACAGAACGC
locus3_HPF16_1428  9    ------------------------------------------------------------
locus3_HPF30_1405  21   ------------------------------------------------------------
locus3_HPF32_1419  410  ---------TAATTTTAGAAATACTTTAATCCCCCTACCCCCTCTAAACGAACAAATCGC
locus3_HPF57_1447  9    ------------------------------------------------------------
locus5_HPSH_07300  398  ---------TAATTTTAGAAATACTTTAATCCCCCTACCCCCCCTAAACGAACAAATCGC
locus5_HPF30_1299  454  A-CTATAGCTGACTTAAGAGATTTATTAATCCCCCTACCCCCTCTAAACGAACAGATCGC
                                              conserved region
locus3_jhp1422     519  TATCGCTAATATTTTAAGCGATGTGGATCGTTACCTTTGTTCTTTAGACGCCCTCATTCT
locus3_HPAG1_1462  450  TATCGCTAACATTTTAAGCGATTTGGATCATTATCTTTATTCTTTAGACGCCCTCATTCT
locus3_HPG27_1455  21   ------------------------------------------------------------
locus3_HPP12_1508  525  TATCGCTAACATTTTAAGCGATTTGGATCATTATCTTTATTCTTTAGACGCCCTCATTCT
locus3_HPSH_07885  453  TATCGCTAACATTTTAAGCGCTTTGGATCGTTATCTTTGTGCATTAGACGCTCTCATTCT
locus3_HPF16_1428  9    ------------------------------------------------------------
locus3_HPF30_1405  21   ------------------------------------------------------------
locus3_HPF32_1419  462  TATCGCTAACATTTTAAGTGCTTTGGATCGCTATCTTTATGCGTTAGACGCTCTCATTCT
locus3_HPF57_1447  9    ------------------------------------------------------------
locus5_HPSH_07300  450  TATCGCTAACATTTTAAGCGCTTTGGATCATTATCTTTATGCATTAGACGCTCTCATTCT
locus5_HPF30_1299  513  TATCGCTAACATTTTAAGCGGTTTGGATCGTTATCTTTGTGCGTTAGACGCTCTCATCCT
```

```
                                       repeat z'
locus3_jhp1422      579  TAAAAAAGAAAGCGTTAAAAAAGCTTTAAGCTTTGAACTATTGAGCCAAAGAAAACGCTT
locus3_HPAG1_1462   510  TAAAAAAGAAAGCGTTAAAAAAGCTTTAAGCTTTGAACTATTGAGCCAAAGAAAACGCTT
locus3_HPG27_1455   21   ------------------------------------------------------------
locus3_HPP12_1508   585  TAAAAAAGAAAGCGTTAAAAAAGCTTTAAGCTTTGAACTATTGAGCCAAAGAAAACGCTT
locus3_HPSH_07885   513  TAAAAAAGAGAGCGTTAAAAAAGCTCTAAGCTTTGAACTATTGAGCCAAAAAAAACGCTT
locus3_HPF16_1428   9    ------------------------------------------------------------
locus3_HPF30_1405   21   ------------------------------------------------------------
locus3_HPF32_1419   522  TAAAAAAGAGGGCGTTAAAAAAGCTTTAAGCTTTGAACTATTGAGCCAAAGAAAACGCTT
locus3_HPF57_1447   9    ------------------------------------------------------------
locus5_HPSH_07300   510  TAAAAAAGAGAGCGTTAAAAAAGCTCTAAGCTTTGAACTATTGAGCCAAAGAAAACGCTT
locus5_HPF30_1299   573  TAAAAAAGAGAGCGTTAAAAAATCCTTAAGCTTTGAACTATTGAGCCAAAGAAAACGCTT
                                         repeat x'
locus3_jhp1422      639  GAAAGGCTTCAATCAAAATTGGCAAAAAGTAAGGCTTGGGGATAT-AGGAATAACTATAA
locus3_HPAG1_1462   570  GAAAGGCTTCAATCAAGCTTGGCAAAGAGTAAGGCTTGGGGATAT-ATGCGAAATCACAA
locus3_HPG27_1455   21   ------------TCAAA-TTGGCAAAGAGTGAGGCTTGGGGATATATGCGAA-ATTAAAA
locus3_HPP12_1508   645  GAAAGGCTTCAATCAAGCTTGGCAAAGAGTGAGGCTTGGGGATAT-ATTTTTCATTACGG
locus3_HPSH_07885   573  GAAAGGTTTCAATCAAGCTTGGCAAAGAGTGAGGCTTGGGGATATTGCCGAA-ATTAAAA
locus3_HPF16_1428   9    ------------TTAAA-TTGGCAAAGAGTAAGGCTTGGAGATATTGCCAACTATTTAAC
locus3_HPF30_1405   21   ------------TTAAA-TTGGCAAAGAGTAAGGCTTGGGGATATTGGCAAACCATGCAT
locus3_HPF32_1419   582  GAAAGGCTTCAATCAAGCTTGGCAAAGAGTAAGGCTTGGGGATATTGCCAACTATTTAAC
locus3_HPF57_1447   9    ------------TTAAA-TTGGCAAAGAGTAAGGCTTGGGGATGTTGGCAAACCATGCAT
locus5_HPSH_07300   570  GAAAGGCTTCAATCAAGCATGGCAAAGAGTGAGGCTTGGGGATATTGCCAACTATTTAAC
locus5_HPF30_1299   633  GAAAGGCTTCAATCAAGCTTGGCAAAGAGTAAGGCTTGGGGATATTGGCAAACCATGCAT

locus3_jhp1422      698  GTGGATTAGCGGGAAAAACAAAGCAAGATTTTATCAATGGCAATGCTAAATATATTACAT
locus3_HPAG1_1462   629  CCGGTT-----CATTAGATGCAA-ATGAAATGGTTCATTATGGAAA-ATATAGATTTTAT
locus3_HPG27_1455   68   GGGGTGTT---CGCATAACAAAAAATGAATTAGATGTATTTGGAAA-----ATACCCTGT
locus3_HPP12_1508   704  CTGGAG----GCGATTTATCTAAGCCGCATTATTCAAATACAAAAC-AAAGTGATTTTA-
locus3_HPSH_07885   632  GGGGTGTT---CGCATAACAAAAAATGAATTAGATGTATTTGGAAA-----ATACCCTGT
locus3_HPF16_1428   57   ATCAAATT---TA---AGTGTAGAACAAATC--ACGCAACAAGGAA-----AAAT--TAA
locus3_HPF30_1405   69   GTGTAA----AAGAGTGATGAAGCACCAAACAACACGATATGGTGA-AATTCCATTCTAT
locus3_HPF32_1419   642  ATCAAA------TTTAAGTGTAGAACAAATCACGCAACAAGGAAAA--ATTAAAGTCTAT
locus3_HPF57_1447   57   GTGTAA----AAGAGTGATGAAGCATCAAACAACACGATATGGTGA-AGTTCCATTCTAC
locus5_HPSH_07300   630  ATCAAA------TTTAAGTGTAGAACAAATCACGCAACAAGGAAAA--ATTAAAGTCTAT
locus5_HPF30_1299   693  GTGTAA----AAGAGTGATGAAGCACCAAACAACACGATATGGTGA-AATTCCATTCTAT

locus3_jhp1422      758  TTTTGAATGTTTTAAATAATGTCATAATAGATACTTCTATACTAGAAAATGTAAAAATAT
locus3_HPAG1_1462   681  -----ACATGTGCAAAAG-----------AATATTATTTTATAGATAAATACGCTTTTG-
locus3_HPG27_1455   119  -------GGTTTCAGGTGG---------AGTTGGCTTTTT-------GGGTTACACAA--
locus3_HPP12_1508   757  -----ATTACCCCATTTA-------TTCAAATGCGATTGA--CAAAAAAGGTTTATATGG
locus3_HPSH_07885   683  -------GGTTTCAGGTGG---------CGTTGGCTTTTT-------GGGTTACACAA--
locus3_HPF16_1428   101  -------AGTCTATGATGT---------GAATAATTTCAT-------AGGCTATACAG--
locus3_HPF30_1405   124  AA--AATAGGCACATTTGGCAACACTGCTGATGCCTTTATTTCAAAAAAGCTATTTTTAG
locus3_HPF32_1419   693  ------------GATGTG-----------AATAATTTCAT-------AGGCTATACAG--
locus3_HPF57_1447   112  AA--AATAGGCACATTTGGCAACACCGCTGATGCCTTTATTTCAAAAAAGCTATTTTTAG
locus5_HPSH_07300   681  ------------GATGTG-----------AATAATTTCAT-------AGGCTATACAG--
locus5_HPF30_1299   748  AA--AATAGGCACATTTGGCAACACTGCTGATGCCTTTATTTCAAAAAAGCTATTTTTAG

locus3_jhp1422      818  ACCCAAATGAAAAACAAAATTCCTTTAAAAAATATGATTTATTCTTTAATACTTCTTCAG
locus3_HPAG1_1462   724  ------ACACAGAAGCAATTTTAATTTCTGGAAATGGTGCGTATGTAGGGTATGTGCATT
locus3_HPG27_1455   154  ------A--CAAC------TT-CAATAGATATGAAAATACAATTACTATCGCTCAATATG
locus3_HPP12_1508   804  A-T---ATTCATCT---TTTTTTATTATTAAAAATAAGAGCATAACAATAACAGCTAGAG
locus3_HPSH_07885   718  ------A--CAAC------TT-CAATAGATATGAAAATACAATTACTATCGCTCAATATG
locus3_HPF16_1428   136  ------ATACAAC------TTTCATTAGTGATAAGCCTTATATTTCTATCGTTAAAGATG
locus3_HPF30_1405   182  AAT---ACAGAACAAAATATTCTTTTCCAAAAAAAGGCGATATTTTAATTTCTGCTTCCG
locus3_HPF32_1419   721  ------ATACAAC------TTTCATTAGTGATAAGCCTTATATTTCTATCGTTAAAGATG
locus3_HPF57_1447   170  AAT---ACAAAACAAAATATTCTTTTCCAAAAAAAGGCGATATTTTAATTTCTGCTTCCG
locus5_HPSH_07300   709  ------ATACAAC------TTTCATTAGTGATAAGCCTTATATTTCTATTGTCAAAGATG
locus5_HPF30_1299   806  AAT---ACAGAACAAAATATTCTTTTCCAAAAAAAGGCGATATTTTAATTTCTGCTTCCG
```

```
locus3_jhp1422      878  AAACTCCAAAAGAAGTAGGCATGTGTGCCGTGCTTTTAGATGATATAGACCAAGTTTTTT
locus3_HPAG1_1462   779  ACTATAAGGGCAAATTTAACGC----------CTATCAAAGAACCTA--------TGTGT
locus3_HPG27_1455   200  GAACGGCTGGTTATGTTA--ATT---------TTCAAA---AAAATA----AATTTTGGG
locus3_HPP12_1508   857  GAACAATGGGTGTTGCT----T----------TTTTTAGAGACTATCCTTATGTCCCTAT
locus3_HPSH_07885   764  GAACGGCTGGCTATGTTA--ACT---------TTCAAA---AAAATA----AATTTTGGG
locus3_HPF16_1428   185  GAAGTGTAGGCAGAGTAAGGATT---------TTGCCACCTAAAACA----AATATTTTA
locus3_HPF30_1405   239  GAACTATTGGTAAGGCAGTCAT----------TTATGACGGAAAACCCGCTTATTTTCAA
locus3_HPF32_1419   770  GAAGTGTAGGCAGAGTAAGGATT---------TTGCCACCTAAAACA----AATATTTTA
locus3_HPF57_1447   227  GAACTATTGGTAGGGCAGTCAT----------TTATGACGGAAAACCCGCTTATTTTCAA
locus5_HPSH_07300   758  GAAGTGTAGGCAGAGTAAGGATT---------TTGCCACCTAAAACA----AATATTTTA
locus5_HPF30_1299   863  GAACTATTGGTAAGGCAGTCAT----------TTATGACGGAAAACCCGCTTATTTTCAA

locus3_jhp1422      938  TGAATAGTTTTTGTTTTGGTTTTAGAATATTTGACAAGGCAGTTGATAGC--TTGTTTTT
locus3_HPAG1_1462   821  TGGATAATTTTAGCGAACATATTATATTTATA-AAATATTTTTTAACAATGTTTTTGCAA
locus3_HPG27_1455   242  CTAATGATGTTTGTTTTTGTATATACCCTAATAAAGATATTATAAAAAATATTTTCTTGT
locus3_HPP12_1508   903  CGGTAGACTTTTAGTCTTACAGCCAAAAATTAGTAATATTGATTGTAGGTT-TTATGCGG
locus3_HPSH_07885   806  CTAATGATGTTTGTTTTTGTATATACCCTAATAAAGATATTATAAAAAATATTTTCTTGT
locus3_HPF16_1428   232  TCTACTATGGGAGCGTTAAT-----TGCTAATCATAGAACAACAACAGAGTTTTTATTTT
locus3_HPF30_1405   289  GACTCAAATATTGTTTGGATTGACAACGATGA--AACATTAGTAAAAAATGATTTTTTAT
locus3_HPF32_1419   817  TCTACTATGGGAGCGTTAATTGCTAATCATAG--AACAACAACAG--AGTTTTTATTTTA
locus3_HPF57_1447   277  GACTCAAATATTGTTTGGATTGACAACGATGA--AACATTAGTAAAAAATGATTTTTTAT
locus5_HPSH_07300   805  TCTACTATGGGAGCGTTAATTGCTAATCATAG--AACAACAACAG--AGTTTTTATTTTA
locus5_HPF30_1299   913  GACTCAAATATTGTTTGGATTGACAACGATGA--AACATTAGTAAAAAATGATTTTTTAT

locus3_jhp1422      996  ATCATACTTAATTAATAGCGAAATAGGCAGAAAAGCTTTTGAAAATTTAGCGCAAGGTTC
locus3_HPAG1_1462   880  TCT---CATATTCAAAC---AAAT-----------------AGAAATGAAGGTA------
locus3_HPG27_1455   302  ATTA---TTTTTTGAAAGTTAATCAAAATTATTTA-TATGAAATTTCAAATAGAAAT-GC
locus3_HPP12_1508   962  AATATATCAATTCAAAAGTAAAAT---------------TTAACACCGAGCAAA----CT
locus3_HPSH_07885   866  ATTA---TTTTTTGAAAGTTAATCAAAATTATTTA-TATGAAATTTCAAATAGAAAT-GC
locus3_HPF16_1428   287  ATTT---GTTATCTAATTTTGATTTTAAAAATTT----------CACAAGCAGTAGC-AT
locus3_HPF30_1405   347  TTTATGCTTATTCTAATGTTAAAT---------------GGAATACAGAGCATA----CA
locus3_HPF32_1419   873  TTTG---TTATCTAATTTTGATTT---------------TAAAAATTTCACAAGCGG--T
locus3_HPF57_1447   335  TTTATGCTTATTCTAATGTTAAAT---------------GGAATACAGAGCATA----CA
locus5_HPSH_07300   861  TTTG---TTATCTAATTTTGATTT---------------TAAAAATTTCACAAGCGG--T
locus5_HPF30_1299   971  TTTATGCTTATTCTAATGTTAAAT---------------GGAATACAGAGCATA----CA

locus3_jhp1422      1056 AACACGATACAATTTATCAAAGAGTGGTTTTAATAATGTTTGCC--TTATTTTACCCCCT
locus3_HPAG1_1462   910  -ATACCCCTT-ATATCGTAACGG-CAACTTTAAAAGATTTTGAAA-TCCTTTTACCCCCT
locus3_HPG27_1455   357  TACACCTTATAGCATTTCAAAAGATAAAATTTTAGATTTTGAAA--TCCCCCTACCTCCC
locus3_HPP12_1508   1003 ACTATTCCACAA-TTAACAATTC-CAAAAGTTGCACTATGTGAAA-TCCTTTTACCCCCC
locus3_HPSH_07885   921  TACACCTTATAGCATTTCAAAAGATAAAATTTTAGATTTTGAAA--TCCTTTTACCCCCT
locus3_HPF16_1428   333  AATACCGCATATTTATTTCAAAGATTATAAAGAAAAGACT---A--TTTTTTTACCCCCT
locus3_HPF30_1405   388  ACTATCTTAAGACTTTATAATGA-TAATTTTAGAAATACTTTAA--TCCCTCTACCCCCT
locus3_HPF32_1419   913  AGCATAATACCGCATATTTATTTCAAAGATTATAAAGAAAAGACTATTTTTTTACCCCCT
locus3_HPF57_1447   376  ACTATCTTAAGACTTTATAATAA-TAATTTTAGAAATACTTTAA--TCCCCCTACCACCT
locus5_HPSH_07300   901  AGCATAATACCGCATATTTATTTCAAAGATTATAAAGAAAAGACTATTTTTTTACCCCCT
locus5_HPF30_1299   1012 ACTATCTTAAGACTTTATAATGA-TAATTTTAGAAATACTTTAA--TCCCTCTACCCCCT
                         repeat y'                                   conserved region
locus3_jhp1422      1114 CTAAACGAACAAATCGCTATCGCTAATATTTTAAGCGATGTGGATAGTGAAATCATTAGC
locus3_HPAG1_1462   967  CTAAACGAACAAATCGCTATCGCTAACATTTTAAGCGATTTGGATAATGAAATCATTAGC
locus3_HPG27_1455   415  CTAAACGAACAAATCGCTATCGCTAACATTTTAAGCGATGTGGATCATGAAATCATTAGC
locus3_HPP12_1508   1060 ATAAACGAACAAATCGCTATCGCTAACATTTTAAGCGCTTTGGATAATGAAATCATTAGT
locus3_HPSH_07885   979  CTAAACGAACAAATCGCTATCGCTAACATTTTAAGCGATTTGGATAATGAGATCGCCAGC
locus3_HPF16_1428   388  CTAAACGAACAGAGCGCTATCGCTAACATTTTAAGCGCTTTGGATAATGAGATCATAAGC
locus3_HPF30_1405   445  CTAAACGAACAGAGCGCTATCGCTAACGTTTTAAGTGCTTTGGATAATGAGATCATAAGC
locus3_HPF32_1419   973  CTAAACGAACAGAGCGCTATCGCTAACATTTTAAGCGCTTTGGATAATGAGATCGCAAGC
locus3_HPF57_1447   433  CTAAACGAACAGAGCGCTATCGCTAACATTTTGAGCGGTTTGGATAATGAGATCGCAAGC
locus5_HPSH_07300   961  CTAAACGAACAAATCGCTATCGCTAACATTTTAAGCGATTTGGATAATGAGATCGCCAGC
locus5_HPF30_1299   1069 CTAAACGAACAGAGCGCTATCGCTAACGTTTTAAGTGCTTTGGATAATGAGATCATAAGC
```

```
                                                  repeat z'
locus3_jhp1422      1174 CTTAAAAACAAAAAACGCCAATTTGAAAATGTCAAAAAAGCCTTAAGC------------
locus3_HPAG1_1462   1027 CTTAAAAACAAAAAACGCCAATTTGAAAGCATCAAAAAAGCTTTAAACCACGATTTAATG
locus3_HPG27_1455   475  CTTAAAAACAAAAAACGCCAATTTGAGAACATCAAAAAAGCTTTAAACCACGATTTGATG
locus3_HPP12_1508   1120 CTTAAAAACAAAAAACGCCAATTTGACAACATCAAAAAAGCTTTAAACCACGATTTAATG
locus3_HPSH_07885   1039 CTTAAAAACAAAAAACGCCAATTTGAAAACATCAAAAAAGCTTTAAACCACGATTTAATG
locus3_HPF16_1428   448  CTTAAAAACAAAAAACGCCAATTTGAAAACATCAAAAAAGCTTTAAACCACGATTTAATG
locus3_HPF30_1405   505  CTTAAAAACAAAAAACGCCAATTTGAAAACATCAAAAAAGCTTTAAACCACGATTTAATG
locus3_HPF32_1419   1033 CTTAAAAACAAAAAACGCCAATTTGAAAACATCAAAAAAGCTTTAAACCATGATTTAATG
locus3_HPF57_1447   493  CTTAAAAACAAAAAACGCCAATTTGAAAACATCAAAAAAGCTTTAAACCACGATTTAATG
locus5_HPSH_07300   1021 CTTAAAAACAAAAAACGCCAATTTGAAAACATCAAAAAAGCTTTAAACCACGATTTAATG
locus5_HPF30_1299   1129 CTTAAAAACAAAAAACGCCAATTTGAAAACATCAAAAAAGCTTTAAACCACGATTTAATG

locus3_jhp1422      1221 ------------------------------------------------------------
locus3_HPAG1_1462   1087 AGCGCTAAAATCAGGGTTTTAGAAAATTAA------------------------------
locus3_HPG27_1455   535  AGCGCTAAAATCAGGGTTTTAAAAAAATTAACGCCCCAAAAGTCAAGGACAAACCCGCTT
locus3_HPP12_1508   1180 AGCGCTAAAATCAGGGTTTTAAACAAATAA------------------------------
locus3_HPSH_07885   1099 AGCGCTAAAATTAGGGTTTTAAAAAAATAA------------------------------
locus3_HPF16_1428   508  AGCGCTAAAATCAGGGTTTTAAAGAAATAA------------------------------
locus3_HPF30_1405   565  AGCGCTAAAATCAGGGTTTTAGAAAAATAA------------------------------
locus3_HPF32_1419   1093 AGTGCTAAAATCAGGGTTTTAAAACAATAA------------------------------
locus3_HPF57_1447   553  AACGCTAAAATTAGGGTTTTAAAAAAATAA------------------------------
locus5_HPSH_07300   1081 AGCGCTAAAATTAGGGTTTTAAAAAAATAA------------------------------
locus5_HPF30_1299   1189 AGCGCTAAAATCAGGGTTTTAGAAAAATAA------------------------------

locus3_jhp1422      1221 ---------------------------------------------TTTGAACTATTGAGC
locus3_HPAG1_1462   1116 ------------------------------------------------------------
locus3_HPG27_1455   595  CATAAGGAAACCCCTAAGCTTTTAGGATTAGGGGAACGCTCTTAG---------------
locus3_HPP12_1508   1209 ------------------------------------------------------------
locus3_HPSH_07885   1128 ------------------------------------------------------------
locus3_HPF16_1428   537  ------------------------------------------------------------
locus3_HPF30_1405   594  ------------------------------------------------------------
locus3_HPF32_1419   1122 ------------------------------------------------------------
locus3_HPF57_1447   582  ------------------------------------------------------------
locus5_HPSH_07300   1110 ------------------------------------------------------------
locus5_HPF30_1299   1218 ------------------------------------------------------------
                                                          repeat x'
locus3_jhp1422      1237 CAAAGAAAACGCTTGAAAGGCTTCAATCAAAATTGGCAAAAAGTAAGGCTTGGGGATATA
locus3_HPAG1_1462   1116 ------------------------------------------------------------
locus3_HPG27_1455   639  ------------------------------------------------------------
locus3_HPP12_1508   1209 ------------------------------------------------------------
locus3_HPSH_07885   1128 ------------------------------------------------------------
locus3_HPF16_1428   537  ------------------------------------------------------------
locus3_HPF30_1405   594  ------------------------------------------------------------
locus3_HPF32_1419   1122 ------------------------------------------------------------
locus3_HPF57_1447   582  ------------------------------------------------------------
locus5_HPSH_07300   1110 ------------------------------------------------------------
locus5_HPF30_1299   1218 ------------------------------------------------------------
                         duplicated TRD
locus3_jhp1422      1297 GGAATAACTATAAGTGGATTAGCGGGAAAAACAAAGCAAGATTTTATCAATGGCAATGCT
locus3_HPAG1_1462   1116 ------------------------------------------------------------
locus3_HPG27_1455   639  ------------------------------------------------------------
locus3_HPP12_1508   1209 ------------------------------------------------------------
locus3_HPSH_07885   1128 ------------------------------------------------------------
locus3_HPF16_1428   537  ------------------------------------------------------------
locus3_HPF30_1405   594  ------------------------------------------------------------
locus3_HPF32_1419   1122 ------------------------------------------------------------
locus3_HPF57_1447   582  ------------------------------------------------------------
locus5_HPSH_07300   1110 ------------------------------------------------------------
locus5_HPF30_1299   1218 ------------------------------------------------------------
```

```
locus3_jhp1422      1357 AAATATATTACATTTTTGAATGTTTTAAATAATGTCATAATAGATACTTCTATACTAGAA
locus3_HPAG1_1462   1116 ------------------------------------------------------------
locus3_HPG27_1455   639  ------------------------------------------------------------
locus3_HPP12_1508   1209 ------------------------------------------------------------
locus3_HPSH_07885   1128 ------------------------------------------------------------
locus3_HPF16_1428   537  ------------------------------------------------------------
locus3_HPF30_1405   594  ------------------------------------------------------------
locus3_HPF32_1419   1122 ------------------------------------------------------------
locus3_HPF57_1447   582  ------------------------------------------------------------
locus5_HPSH_07300   1110 ------------------------------------------------------------
locus5_HPF30_1299   1218 ------------------------------------------------------------

locus3_jhp1422      1417 AATGTAAAAATATACCCAAATGAAAAACAAAATTCCTTTAAAAAATATGATTTATTCTTT
locus3_HPAG1_1462   1116 ------------------------------------------------------------
locus3_HPG27_1455   639  ------------------------------------------------------------
locus3_HPP12_1508   1209 ------------------------------------------------------------
locus3_HPSH_07885   1128 ------------------------------------------------------------
locus3_HPF16_1428   537  ------------------------------------------------------------
locus3_HPF30_1405   594  ------------------------------------------------------------
locus3_HPF32_1419   1122 ------------------------------------------------------------
locus3_HPF57_1447   582  ------------------------------------------------------------
locus5_HPSH_07300   1110 ------------------------------------------------------------
locus5_HPF30_1299   1218 ------------------------------------------------------------

locus3_jhp1422      1477 AATACTTCTTCAGAAACTCCAAAAGAAGTAGGCATGTGTGCCGTGCTTTTAGATGATATA
locus3_HPAG1_1462   1116 ------------------------------------------------------------
locus3_HPG27_1455   639  ------------------------------------------------------------
locus3_HPP12_1508   1209 ------------------------------------------------------------
locus3_HPSH_07885   1128 ------------------------------------------------------------
locus3_HPF16_1428   537  ------------------------------------------------------------
locus3_HPF30_1405   594  ------------------------------------------------------------
locus3_HPF32_1419   1122 ------------------------------------------------------------
locus3_HPF57_1447   582  ------------------------------------------------------------
locus5_HPSH_07300   1110 ------------------------------------------------------------
locus5_HPF30_1299   1218 ------------------------------------------------------------

locus3_jhp1422      1537 GACCAAGTTTTTTTGAATAGTTTTTGTTTTGGTTTTAGAATATTTGACAAGGCAGTTGAT
locus3_HPAG1_1462   1116 ------------------------------------------------------------
locus3_HPG27_1455   639  ------------------------------------------------------------
locus3_HPP12_1508   1209 ------------------------------------------------------------
locus3_HPSH_07885   1128 ------------------------------------------------------------
locus3_HPF16_1428   537  ------------------------------------------------------------
locus3_HPF30_1405   594  ------------------------------------------------------------
locus3_HPF32_1419   1122 ------------------------------------------------------------
locus3_HPF57_1447   582  ------------------------------------------------------------
locus5_HPSH_07300   1110 ------------------------------------------------------------
locus5_HPF30_1299   1218 ------------------------------------------------------------

locus3_jhp1422      1597 AGCTTGTTTTTATCATACTTAATTAATAGCGAAATAGGCAGAAAAGCTTTTGAAAATTTA
locus3_HPAG1_1462   1116 ------------------------------------------------------------
locus3_HPG27_1455   639  ------------------------------------------------------------
locus3_HPP12_1508   1209 ------------------------------------------------------------
locus3_HPSH_07885   1128 ------------------------------------------------------------
locus3_HPF16_1428   537  ------------------------------------------------------------
locus3_HPF30_1405   594  ------------------------------------------------------------
locus3_HPF32_1419   1122 ------------------------------------------------------------
locus3_HPF57_1447   582  ------------------------------------------------------------
locus5_HPSH_07300   1110 ------------------------------------------------------------
locus5_HPF30_1299   1218 ------------------------------------------------------------
```

```
locus3_jhp1422      1657 GCGCAAGGTTCAACACGATACAATTTATCAAAGAGTGGTTTTAATAATGTTTGCCTTATT
locus3_HPAG1_1462   1116 ------------------------------------------------------------
locus3_HPG27_1455   639  ------------------------------------------------------------
locus3_HPP12_1508   1209 ------------------------------------------------------------
locus3_HPSH_07885   1128 ------------------------------------------------------------
locus3_HPF16_1428   537  ------------------------------------------------------------
locus3_HPF30_1405   594  ------------------------------------------------------------
locus3_HPF32_1419   1122 ------------------------------------------------------------
locus3_HPF57_1447   582  ------------------------------------------------------------
locus5_HPSH_07300   1110 ------------------------------------------------------------
locus5_HPF30_1299   1218 ------------------------------------------------------------
                           repeat y'
locus3_jhp1422      1717 TTACCCCCTCTAAACGAACAAATCGCTATCGCTAATATTTTAAGCGATGTGGATAGTGAA
locus3_HPAG1_1462   1116 ------------------------------------------------------------
locus3_HPG27_1455   639  ------------------------------------------------------------
locus3_HPP12_1508   1209 ------------------------------------------------------------
locus3_HPSH_07885   1128 ------------------------------------------------------------
locus3_HPF16_1428   537  ------------------------------------------------------------
locus3_HPF30_1405   594  ------------------------------------------------------------
locus3_HPF32_1419   1122 ------------------------------------------------------------
locus3_HPF57_1447   582  ------------------------------------------------------------
locus5_HPSH_07300   1110 ------------------------------------------------------------
locus5_HPF30_1299   1218 ------------------------------------------------------------
                                                                  repeat z'
locus3_jhp1422      1777 ATCATTAGCCTTAAAAACAAAAAACGCCAATTTGAAAATGTCAAAAAAGCCTTAAACCAC
locus3_HPAG1_1462   1116 ------------------------------------------------------------
locus3_HPG27_1455   639  ------------------------------------------------------------
locus3_HPP12_1508   1209 ------------------------------------------------------------
locus3_HPSH_07885   1128 ------------------------------------------------------------
locus3_HPF16_1428   537  ------------------------------------------------------------
locus3_HPF30_1405   594  ------------------------------------------------------------
locus3_HPF32_1419   1122 ------------------------------------------------------------
locus3_HPF57_1447   582  ------------------------------------------------------------
locus5_HPSH_07300   1110 ------------------------------------------------------------
locus5_HPF30_1299   1218 ------------------------------------------------------------

locus3_jhp1422      1837 GATTTAATGAGCGCTAAAATCAGGGTTTTAAACAAATAA
locus3_HPAG1_1462   1116 ---------------------------------------
locus3_HPG27_1455   639  ---------------------------------------
locus3_HPP12_1508   1209 ---------------------------------------
locus3_HPSH_07885   1128 ---------------------------------------
locus3_HPF16_1428   537  ---------------------------------------
locus3_HPF30_1405   594  ---------------------------------------
locus3_HPF32_1419   1122 ---------------------------------------
locus3_HPF57_1447   582  ---------------------------------------
locus5_HPSH_07300   1110 ---------------------------------------
locus5_HPF30_1299   1218 ---------------------------------------
```
