# Supplementary material for: Domain Movement within a Gene: A Novel Evolutionary Mechanism for Protein Diversification
Source: PLoS One. 2011 Apr 14;6(4):e18819. doi: 10.1371/journal.pone.0018819 (PMC3077401; doi:10.1371/journal.pone.0018819)
Supplement: Figure S4 — Nucleotide sequence alignment of the S subunit of Type IIG systems (See Fig. 5 ). (PDF) [file pone.0018819.s004.pdf]

```
                                             conserved region                                 repeat x''
HP1471                     1    ----------------------------------------------------TTGGGGATTTG
jhp1364                    1    ----GTGATTGGCCCCCTTAGCAGCCAACTCAACGCTATTAAGTGGGGCGAGTTCAAATTAGGGGATTTG
HPAG1_1444                 1    ----GTGATTGGCCCCCTTAGTAGCCAACTCAACGCTATTAAGTGGGGCGAGTT----------------
HPG27_1394                 1    ----GTGATTGGCCCCCTTAGCAGCCAACTCAACGCTATTAAGTGGGGCGAGTTCAAATTAGGGGATTTG
HPP12_1449                 1    ----GTGATTGGCCCCCTTAGTAGCCAACTCAACGCTATTAAGTGGGGCGAGTTCAAATTAGGGGATTTG
HPSH_7535                  1    -----------GGCCCCTTAGTAGCCAACTCAACGCTATTAAGTGGGGCGAGTTCAAATTAGGGGATTTG
HPF16_1367-HPF16_1368      1    CAAAGTGATTGGCCCCCTTAGTAGCCAACTCAACGCTATTAAGTGGGGCGAGTT----------------
HPF30_1338-HPF30_1339      1    ----GTGATTGGCCCCCTTAGTAGCCAACTCAACGCTATTAAGTGGGGCGAGTTCAAATTGGGGGATTTG
HPF32_1358                 1    ----GTGATTGGCCCCTTTAGTAGCCAACTCAGCGCTATTAAGTGGGGCGAGTTCAAATTAGGGGATTTG
HPF57_1386                 1    ----GTGATTGGCCTCCTTAGTAGCCAACTCAACGCTATTAAGTGGGGCGAGTTCAAATTGGGGGATTTG
KHP_1322                   1    ----GTGATTGGCCCCCTTAGTAGCCAACTCAACGCTATTAAGTGGGGCGAGTTCAAATTAGTGGATTTG
HPKB_1373                  1    ----------------------------------------------------------------------
hp908_1453-hp908_1454      1    ----GTGATTGGCCCCCTTAGTAGCCAACTCAACGCTATTAAGTGGGGCGAGTTCAAATTAGGGGATTTG
HELPY_1443-HELPY_1444      1    ----GTGATTGGCCCCCTTAGCAGCCAACTCAACGCTATTAAGTGGGGCGAGTTCAGATTGGGGGATTTG
HPB8_59-HPB8_60            1    ----GTGATTGGCCCCCTTAGTGGCCAACTCAACGCTATTAAGTGGGGCGAGTTCAGATTAGGGGATTTG
HPCU_07415                 1    ----GTGATTGGCCCCCTTAGTAGCCAACTCAACGCTATTAAGTGGGGCGAGTTCAAATTGGGGGATTTG
HPSJM_07500                1    ----GTGATTGGCCCCCTTAATAGCCAACTCAACGCTATTAAGTGGGGCGAGTTCAAATTAGGGGATTTG
HPSAT_07075                1    ----GTGATTGGCCCCCTTAGTAGCCAACTCAACGCTATTAAGTGGGGCGAGTTCAGATTAGGGGATTTG
HPV225_1544                1    -------ATTGGCCCCCTTAGTAGCCAACTCAACGCTATTAAGTGGGGCGAGTTCAAATTAGGGGATTTG

                                      TRD1
HP1471                     13   TTTGAAGTGTTGTCAAGTAAGAAAATTTATCATGCCAACACGATAAAA-ATCCATGACACGCAAATAGAA
jhp1364                    67   TTTGAAGCGA-GTAACG---GCGATTTTGACATTCAAAAACGCCACATCAATCATAAGGGCGAATTTGTC
HPAG1_1444                 50   --------TTCATACGAAACCATATACGAGCTACAAAAAACAACAAAATTATTAAGCAAAAAAGATTGCG
HPG27_1394                 67   TTTGAAGCGA----GTAACGGCGATTTTGACATTCAAAAACGCCACATCAATCATAAGGGCGAATTTGTC
HPP12_1449                 67   TTTGAAGTGTTGCCAAGTAAGAAAATTTATCATGCCAATACGATAAAA-ATCCATGACACGCAAATAGAA
HPSH_7535                  60   TTTGAAGCGA----GTAACGGCGATTTTGACATTCAAAAACGCCACATCAATCATAAGGGCGAATTTGTC
HPF16_1367-HPF16_1368      54   --------TTCATACGAAACCATATACGAGCTACAAAAAACAACAAAATTATTAAGCAAAAAAGATTGCG
HPF30_1338-HPF30_1339      67   TTTGAGATCTACACTGGCTCTTTATTAAGCCAAGAGGATTTAGCAAAA-GGTGATATTGTCCGCATATCA
HPF32_1358                 67   TTTGAGATCTACACTGGCTCTTTATTAAGCCAAGAGGATTTAGCAAAA-GGTGATATTGTCCGCATATCA
HPF57_1386                 67   TTTGAGGTCTACACTGGCTCTTTATTAAGCCAAGAGGATTTAGCAAAA-GGTGATATTGTCCGCATATCA
KHP_1322                   67   TTTGAAAAAATTATTATAAAGCCATTACCTTATCAAACTGCACAATTACCAAAGGAAAAAACTCCAACGC
HPKB_1373                  1    -------------------------TTTGACATTCAAAAACGCCACATCAATCATAAGGGCGAATTTGTC
hp908_1453-hp908_1454      67   TTTGAAAAAATTATTATAAAGCCATTACCTTATCAAACTGCACAATTACCAAAGGAAAAAACTCCAACGC
HELPY_1443-HELPY_1444      67   TTTGAAAAAATTATTATAAAGCCATTACCTTATCAAACTGCACAATTACCAAAGGAAAAAACTCCAACGC
HPB8_59-HPB8_60            67   TTTGAGATCT----ACACTGGCTCTTTATTAAGCCAAGAGGATTTAGCAAAAGGTGATATTGTCCGCATA
HPCU_07415                 67   TTTGAAGCGA----GTAACGGCGATTTTGACATTCAAAAACGCCACATCAATCATAAGGGCGAATTTGTC
HPSJM_07500                67   TTTGAAATTG----AAAAAACATTAAGCTTTAATAAAGACGCTTTAACGCAAGGACAAGATTACGACTAC
HPSAT_07075                67   TTTGAA----------------------------------------------------------------
HPV225_1544                64   TTTGAAATTG----AAAAAACCTTAAGCTTTAATAAGGACGCTTTAACACAAGGACAAGACTACGATTAT

HP1471                     82   AACA--GCTACCCTTA-TGTCGTGCGCGCTGCAACCAATAATGGTATAAAAGGCTTTATTATAGATGACC
jhp1364                    133  ATCACCGCAGGGCTTAGCAATAATGGCGTTTTAGGGCAAAGCGATATAAAAGCAAAAGTTTTTGAAAGCC
HPAG1_1444                 113  TTGAAAATGGAAAAATACCATTATTTACATCAGATAGTAAGAATAATGGCATTGTTGGTTATGTGAATTG
HPG27_1394                 133  ATCACTGCAGGGCTTAGCAATAATGGTGTTTTAGGGCAAAGCGATATTAAAGCAAAAGTTTTTGAAAGCC
HPP12_1449                 136  AACA--GCTACCCTTA-TGTCGTGTGCGCTACAACCAACAATGGTATAAAAGGCTTTATTATAGATGACC
HPSH_7535                  126  ATCACTGCAGGGCTTAGCAATAATGGCGTTTTAGGGCAAAGCGATATAAAAGCAAAAGTTTTTGAAAGCC
HPF16_1367-HPF16_1368      117  TTGAAAATGGAAAAATACCATTATTTACATCAGATAGTAAGAATAATGGCATTGTTGGTTATGTGAATTG
HPF30_1338-HPF30_1339      136  GTTAAAAGTGATAATAATGGCGTTTATGGTCATTTTGACACTTTAAAAAATAAGAAAGCAAGGCATTTTG
HPF32_1358                 136  GTTAAAAGTGATAATAATGGCGTTTATGGTCATTTTGACACCTTAAAAAATAAGAAAGCAAGGCATTTTG
HPF57_1386                 136  GTTAAAAGTGATAATAATGGCGTTTATGGTCATTTTGACACCTTAAAAAATAAGAAAGCAAGGCATTTTG
KHP_1322                   137  ACGAATTACCAGCCCTAACCGCAGGGATTTTAAATCAAGGGTTAAATAATTTTGTGCCAAAAGAAAACGC
HPKB_1373                  46   ATCACCGCAGGGCTTAGCAATAATGGCGTTTTAGGGCAAAGCGATATAAAAGCAAAAGCTTTTGAAAGCC
hp908_1453-hp908_1454      137  ACGAATTACCAGCCCTAACCGCAGGAATTTTAAATCAAGGGTTAAATAATTTTGTGCCAAAAGAAAATGC
HELPY_1443-HELPY_1444      137  ACGAATTACCAGCCCTAACCGCAGGGATTTTAAATCAAGGGTTAAATAATTTTGTGCCAAAAGAAAATGC
HPB8_59-HPB8_60            133  TCAGTTAAAAGTGATAATAATGGCGTTTATGGTCATTTTGACACCTTAAAAAATAAGAAAGCAAGGCATT
HPCU_07415                 133  ATCACTGCAGGGCTTAGCAATAATGGCGTTTTAGGGCAAAGCGATATAAAAGCAAAAGTTTTTGAAAGCC
HPSJM_07500                133  ATTACAAGGACTTCGCAAAATCAAGGCGTTTTGCAAACCACAGGATTTGTCAATGCAGAAAATTTAAACC
HPSAT_07075                72   ----------------------------------------------------------------------
HPV225_1544                130  ATTACAAGGACTTCGCAAAATCAAGGCGTTTTGCAAACCACAGGATTTGTCAATGCAGAAAATTTAAACC
```

```
HP1471                     149  CTACATTTGCTAATAAAAAAAATACCCTTTCGTTCGCGCAAGACACTTTCACTGTGTTTTATCAAAAACA
jhp1364                    203  ATACCATTACTATTGA--------CATGTTTGGTTGCGCG------TTTTATCGCAGTTTTGCTTATAAA
HPAG1_1444                 183  TAAGCCTAGTTATAAATTAAAAAATAATCAATGCATGGTAGTATTTGGGGATCATACGAGAACATTTAAT
HPG27_1394                 203  ATACCATTACTATTGAC--------ATGTTTGGTTGCGCGTTTTATCGCAGTTTTGCTTATAAAATGGTA
HPP12_1449                 203  CTACATTTACTAATGAAAAAAATACCCTTTCGTTCGCGCAAGACACTTTCACCGTGTTCTATCAAAAACA
HPSH_7535                  196  ATAGCATTACTATTGAC--------ATGTTTGGTTGCGCGTTTTATCGCAGTTTTCCTTATAAAATGGTA
HPF16_1367-HPF16_1368      187  TAAGCCTAGTTATAAATTAAAAAATAATCAATGCATGGTAGTATTCGGAGACCATACGAGAACATTTAAT
HPF30_1338-HPF30_1339      206  AAAATTTTATCAGCGTA--------AATTTTTTTGGCAATTGTTTTTATCACCCTTATTTGGCAAGCGTA
HPF32_1358                 206  AAAATTTTATCAGCGTA--------AATTTTTTTGGCAATTGTTTTTATCATCCTTATTTGGCAAGCGTA
HPF57_1386                 206  AAAATTTTATCAGCGTA--------AATTTTTTTGGCAATTGTTTTTATCACCCTTATTTGGCAAGCGTA
KHP_1322                   207  CACGATTTTAAAAAATGTTATTTCAATTTCTGCTAATGGGGCTAACACCGGGGCTACATTTTACCAACCT
HPKB_1373                  116  ATAGCATTACTATTGA--------CATGTTTGGTTGCGCG------TTTTATCGCAGTTTTCCTTATAAA
hp908_1453-hp908_1454      207  TACGATTTTAAAAAATGTTATTTCAATTTCTGCTAATGGGGCTAACACCGGGGCTACATTTTACCAACCC
HELPY_1443-HELPY_1444      207  CACGATTTTAAAAAATGTTATTTCAATTTCTGCTAATGGAGCTAACACCGGGGCTACATTTTACCAACCC
HPB8_59-HPB8_60            203  TTGAAAATTTTATCAGC--------GTAAATTTTTTTGGCAATTGTTTTTATCACCCTTATTTGGCAAGC
HPCU_07415                 203  ATAGCATTACTATTGAC--------ATGTTTGGTTGCGCGTTTTATCGCAGTTTTCCTTATAAAATGGTA
HPSJM_07500                202  -CACCATTTACTTGGAG--------TTTAGGGCTTTTACAAATGGATTTTTTTCTATCGTAAAAAGTCATG
HPSAT_07075                72   ----------------------------------------------------------------------
HPV225_1544                199  -CACCATTTACTTGGAG--------TTTAGGGCTTTTGCAAATGGATTTTTTTCTATCGTAAAAAGTCATG

HP1471                     219  ACCTTATTTTACAGGCAATAAGGTTAAAATTTTAAAACCAAAATTTGCTTTCAAAAGCCCTAAAATT--T
jhp1364                    259  ATGGTA----ACACATGCTAGGGTATTTTCTCTCAAACCTAAATTTGAAATCAA---CCATAAAATCGGC
HPAG1_1444                 253  ATTGCTAAAAATGATTTTTGTATCGCAGATAATGTAAAAGTTTTAAAGCCCATAAAAGATTTTAGCATAA
HPG27_1394                 265  ACACATGCTAGGGTATTTTCTC----TCAAACCTAAATTTGAAATCAACCATAAAATCGGCTTGTTTTTA
HPP12_1449                 273  ACCTTATTTTACAGGCAATAAGGTTAAAATTTTAAAACCAAAATTTGCTTTCAAAAGCCCTAAAATT--T
HPSH_7535                  258  ACACACGCTAGGGTATTTTCTC----TCAAACCTAAATTTGAAATCAACCATAAAATCGGCTTGTTTTTA
HPF16_1367-HPF16_1368      257  ATTGCTAAAAATGATTTTTGTATCGCAGATAATGTAAAAGTTTTAAAGCCTATAAAAGATTTTAGCATAA
HPF30_1338-HPF30_1339      268  GAGATG------AAAGTCCATGTCTTAAAATTAAAAAATCGTACTCTTACAAAACGGATAGGATTATTTC
HPF32_1358                 268  GAGATG------AAAGTCCATGTCTTAAAATTAAAAAATCGTACTCTTACAAAACGGATAGGATTATTTC
HPF57_1386                 268  GAGATG------AAAGTCCATGTCTTAAAATTAAAAAATCGTACTCTTACAAAACGGATAGGATTATTTC
KHP_1322                   277  CATGAATTTTGCGTATTACAAGACGCTTACGCTATTGAATTTATTGGTGATAAAAAGCTTAACGATAAAG
HPKB_1373                  172  ATGGTA----ACACATGCTAGGGTATTTTCTCTCAAACCTAAATTTGAAATCAA---CCATAAAATCGGC
hp908_1453-hp908_1454      277  CATGAATTTTGCGTATTACAAGACGCTTATGCTATTGAATTTATTGGCGATAAAAAGCTTAACGATAAAG
HELPY_1443-HELPY_1444      277  CATGAATTTTGCGTATTACAAGATGCTTACGCTATTGAATTTATTGGTGGTAAAAAGCTTAACGATAAAG
HPB8_59-HPB8_60            265  GTAGAGATGAAAGTCCATGTCT----TAAAATTAAAAAATCGTACTC-TTACAAAACGGGTAGGATTATT
HPCU_07415                 265  ACACACGCTAGGGTATTTTCTC----TCAAACCTAAATTTGAAATCAACCATAAAATCGGCTTGTTTTTA
HPSJM_07500                264  GTATGCGGGACAATTCATGCGAAAAATCACGCCAAAAGCTGAAATCAAAAATAAAATTAATTCGCGCACA
HPSAT_07075                72   ----------------------------------------------------------------------
HPV225_1544                261  GTATGCGGGACAGTTCATGCGAAAAATCACACCAAAAACTGAAATTGAAAATAAAATTAATTCACGCATA

HP1471                     287  TACATTCTATAAGCGCGATTTTACAATTTATTTTAAAACCCTTAACTTGGGGGCTAGGCTC---------
jhp1364                    322  TTGTTTTTATCCACGCTATTTTTTGGTT-ACCATAAAAAATTCGGCTATGAAAACATG-TG---------
HPAG1_1444                 323  GGGTTTTGTTATTCATTAACACTATGTGGGGTAAAAAAATCATTGATAAGGGTTACGCTAG---------
HPG27_1394                 331  TCCACGCTATT--------TTTTGGTTACCCTAAAAAATTCGGCTATGAAAACATGTGTT----------
HPP12_1449                 341  TATATTTTATAAGCGCGATTTTACAATTTATTTTAAAACCCTTAACTTGGGGGCTAGGCTC---------
HPSH_7535                  324  TCCACGCTATT--------TTTTGATTACCCTAAAAAATTCGGCTATGAAAACATGTGTT----------
HPF16_1367-HPF16_1368      327  GGATTTTGTTATTCATTAACACTATGTGGGGTAAAAAAATCATTGATAAGGGTTACGCTAG---------
HPF30_1338-HPF30_1339      332  TTGCCAATCAACTTAATAAGTGTTTTTATGGTCAATTTACCTATGGAACGCAATTATCTAG---------
HPF32_1358                 332  TTGCCAATCAACTTAATAAGTGTTTTTATGGTCAATTTACCTATGGAACGCAATTATCTAG---------
HPF57_1386                 332  TTGCCAATCAACTTAATAAGTGTTTTTATGGTCAATTTACCTATGGAACGCAATTATCTAG---------
KHP_1322                   347  AATATTTATTTTTTGTCTGTGCTATTTCAAAAGTTATTTATAATAATAGTAAATACGAATGGACAAATAA
HPKB_1373                  235  TTGTTTTTATCCACGCTATTTTTTGATT-ACCCTAAAAAATTCGGCTATGAAAACATG-TG---------
hp908_1453-hp908_1454      347  AATATTTATTTTTTGTATGTGCTATTTCAAAAGTTATTTATAATAATAGTAAATACGAATGGACCAATAA
HELPY_1443-HELPY_1444      347  AATATTTATTTTTTGTATGTGCTATTTCAAAAGTTATTTATAATAATAGTAAATACGAATGGACAAATAA
HPB8_59-HPB8_60            330  TCTTGCCAATCAACTTAATAAGTGTTTTTATGGTCAATTTACCTATGGAACGCAATTATCTAGCTCTAAA
HPCU_07415                 331  TCCACGCTATT--------TTTTGATTACCCTAAAAAATTCGGCTATGAAAACATGTGTT-----CATGG
HPSJM_07500                334  GCCCACTATTTCACAACGCTTTTAAACGCCTTAAAACGCCCTTTATTGAGTGTATTGGTTAGAGATATTG
HPSAT_07075                72   ----------------------------------------------------------------------
HPV225_1544                331  GCCCACTACTTCACAACGCTTTTAAACGCCTTAAAACGCCCTTTATTAAGCGTATTGGTTAGAGATATTG
```

```
                                        repeat y" (annotation bar over following block); center repeat region (annotation bar over third block)
```

```
HP1471                      347  ---------------------------TACAACAGAAAGCATTGCGGAGTTTAAATTTTCTCTACCCCTA
jhp1364                     380  ---------------------------TTCATGGGCAAAAATTAAAAACGATAAAGTCATTCTACCCCTA
HPAG1_1444                  383  ---------------------------GCATTGGAGCTTGGCAAAAACTGCAAAAATCCAACTACCCCTA
HPG27_1394                  382  ---------------------------CATGGGTAAAAATT--AAAAACGATAAGGTCATTCTATCCCTA
HPP12_1449                  401  ---------------------------TACAACAGAAAGCATTGCGGAATTTAAATTTTCTCTACCCCTA
HPSH_7535                   375  ---------------------------CATGGGCAAAAATT--AAAAACGATAAAGTCATTCTACCCCTA
HPF16_1367-HPF16_1368       387  ---------------------------GCATTGGAGCTTGGCTAAAACTGCAAAAATCCAACTACCCCTA
HPF30_1338-HPF30_1339       392  ---------------------------CTCTAAATTAAAACATAATAATTTTAAAATCCAACTACCCCTA
HPF32_1358                  392  ---------------------------CTCTAAATTAAAACATAATAATTTTAAAATCCAACTACCCCTA
HPF57_1386                  392  ---------------------------CTCTAAATTAAAACATAATAATTTTAAAATCCAACTACCCCTA
KHP_1322                    417  AGCAGGGTGGAATAAAGTTAAAAACGAGTTAATTTCT------------------------CTACCCCTA
HPKB_1373                   293  ---------------------------TTCATGGGCAAAAATTAAAAACGATAAAGTCATTCTACCCCTA
hp908_1453-hp908_1454       417  GGCGAGTTGGAATAAAGTTAAAAACGAGTTAATTTCT------------------------CTCCCCCTA
HELPY_1443-HELPY_1444       417  AGCAGGGTGGAATAAAGTTAAAAACGAGTTAATTTCT------------------------CTACCCCTA
HPB8_59-HPB8_60             400  TTAAAACAT----------------------------------AATAATTTTAAGATCCAACTACCCCTA
HPCU_07415                  388  GCAAAAATT----------------------------------AAAAACGATAAAGTCATTCTACCCCTA
HPSJM_07500                 404  ATAAAACTTTTAGGG--------------------------------AGCAAAAAATCCAACTACCCCTA
HPSAT_07075                 72   ----------------------------------------------------------------------
HPV225_1544                 401  ATAAAACTTTTAGGGAGCAA--------------------------------AAAATCCAACTACCCCTA

                                                         repeat y"

HP1471                      391  AAACCCACCGCTAACGCTCAAACCCTTGAGGATATTGATTTTGATTTCATGGAAAAATTCATAGCCGAAC
jhp1364                     424  AAACCCACCGCTAACACTCAAACCCTTGAGGGTATTGATTTTGATTTCATGGAAAAATTCATAGCCGAAC
HPAG1_1444                  427  AAACCCACCGCTAAAACTCAAACCCTTGAGGACATTGATTTTGATTTCATGGAAAAATTCATAGCCGAAC
HPG27_1394                  424  AAACCCACCGCTAACACTCAAACCCTTGATGATATTGATTTTGATTTCATGGAAAAATTCATAGCCGAAC
HPP12_1449                  445  AAACCCACCGCTAACACTCAAACCCTTGATGGTATTGATTTTGATTTCATGGAAAAATTCATAGCCGAAC
HPSH_7535                   417  AAACCCAGCACTAACACTCAAACCCTTGAGGATATTGATTTTCATTTCATGGAAAAATTCATAGCCGAAC
HPF16_1367-HPF16_1368       431  AAACCCACCGCTAAAACTCAAACCCTTAAGGATATTGATTTCAATTTCATGGAAAAATTCATAGCCGAAC
HPF30_1338-HPF30_1339       436  AAACCCACTGCTAAAACTCAAACTCTTAAGGATATTGATTTCAATTTCGTGGAAAAATTCATAGCCGAAC
HPF32_1358                  436  AAACCCACCGCTAACGCTCAAACCCTTAAGGATATTGATTTCAATTTCATGGAAAAATTCATAGCCGAAC
HPF57_1386                  436  AAACCCACCGCTAACACTCAAACCCTTAAGGACATTGATTTTCATTTCATGGAAAAATTCATAGCCGAAC
KHP_1322                    463  AAACCCACCGCTAAAACTCAAACCCTTAAGGATATTGATTTCAATTTCATGGAAAAATTCATAGCCGAAC
HPKB_1373                   337  AAACCCACCGCTAACACTCAAACCCTTAAGGATATTGATTTCAATTTCATGGAAAAGTTCATCGCCGAAC
hp908_1453-hp908_1454       463  AAACCCACCGCTAACGCTCAAACCTTTGAGGATATTGATTTTCATTTCATGGAAAAATTCATAGCCGAAC
HELPY_1443-HELPY_1444       463  AAACCCACCGCTAAAATTCAAACCCTTGAGGGCATTGATTTTGATTTCATGGAAAAATTCATAGCCGAAC
HPB8_59-HPB8_60             436  AAACCCACCGCTAAAATTCAAACCCTTGAGGATATTGATTTTGATTTCATGGAAAAATTCATAGCCGAAC
HPCU_07415                  424  AAACCCACCGCTAACACTCAAACCCTTGATGATATAGATTTTCATTTCATGGAAAAATTCATAGCCGAAC
HPSJM_07500                 442  AAACCCACCGCTAAAAATCAAACCCTTGAGAATATTGATTTTGATTTCATGGAACAATTCATAGCCGAAC
HPSAT_07075                 72   ----------------------------------------------------------------------
HPV225_1544                 439  AAACCCACCGCTAACACTCAAACCCTTGATGGTATTGATTTTCATTTCATGGAAAAATTCATAGCCGAAC

                                        center repeat region

HP1471                      461  TTGAGCAGTGTCGGCTCGCCGAACTTGAGCAG--------------------------------------
jhp1364                     494  TTGAGCAG--------------------------------------------------------------
HPAG1_1444                  497  TTGAGCAG--------------------------------------------------------------
HPG27_1394                  494  TTGAGCAGTGTCGGCTCGCCGAACTTCAG-----------------------------------------
HPP12_1449                  515  TTGAGCAG--------------------------------------------------------------
HPSH_7535                   487  TTGAGCAGTGTCGGCTCGCCGAACTTGAG-----------------------------------------
HPF16_1367-HPF16_1368       501  TTGAG-----------------------------------------------------------------
HPF30_1338-HPF30_1339       506  TTGAGCAGTGTCGGCTCGCCGAACTTGAG-----------------------------------------
HPF32_1358                  506  TTGAGCAGTGTCGGCTCGCCGAACTTGAGCAG--------------------------------------
HPF57_1386                  506  TTGAG-----------------------------------------------------------------
KHP_1322                    533  TTGAGCAGTGTCGGCTCGCCGAACTTGAGCAGTGTCGGCTCGCCGAACTTGAGCAGTGTCGGCTCGCCGA
HPKB_1373                   407  TTGAGCAGTGTCGGCTCGCCGAACTTGAG-----------------------------------------
hp908_1453-hp908_1454       533  TTGAGCAGTGTCGGCTCGCCGAACTTGAG-----------------------------------------
HELPY_1443-HELPY_1444       533  TTGAGCAGTGTCGGCTCGCCGAACTTCAG-----------------------------------------
HPB8_59-HPB8_60             506  TTGAGCAGTGTCGGCTCGCCGAACTTGAG-----------------------------------------
HPCU_07415                  494  TTGAGCAGTGTCGGCTCGCCGAACTTGAG-----------------------------------------
HPSJM_07500                 512  TTGAGCAGTGTCGGCTCGCCGAACTTGAG-----------------------------------------
HPSAT_07075                 72   ----------------------------------------------------------------------
HPV225_1544                 509  TTGAGCAGTGTCGGCTCGCCGAACTTGAGCAGTGTCGGCTCGCCGAACTTGAGCAGTGTCGGCTCGCCGA
```

```
                                                                    conserved region
HP1471                     492  ----------TGTCGGCTCGCCGAACTTCAGGCTTATTTAAAAGCTACAGGGCTAGAAAACACCACCCTT
jhp1364                    501  ----------TGTCGGCTCGCCGAACTTCAGGCTTATTTAAAAGCTACAGGGCTAGAAAACACCACCCTT
HPAG1_1444                 504  ----------TGTCGGCTCGCCGAACTTGAGGCTTATTTAAAAGCTACAGGGCTAGAAAACACCACCCTT
HPG27_1394                 522  -------------------------------GCTTATTTAAAAGCTACAGGGCTAGAAAACACCACCCTT
HPP12_1449                 522  ----------TGTCGGCTCGCCGAACTTCAGGCTTATTTAAAAGCTACAGGGCTAGAAAACACCACCCTT
HPSH_7535                  515  -------------------------------GCTTATTTAAAAGCTACAGGGCTATCAAACACCACCCTT
HPF16_1367-HPF16_1368      505  -------------------------------GCTTATTTAAAAGCTACAGGACTATCAAACACCACCCTT
HPF30_1338-HPF30_1339      534  -------------------------------GCTTATTTAAAAGCTACAGGGTTATCAAACACCACCCTT
HPF32_1358                 537  ----------TGTCGGCTCGCCGAACTTGAGGCTTATTTAAAAGCTACAGGGCTATCCAACACCACCCTT
HPF57_1386                 510  -------------------------------GCTTATTTAAAAGCTACAGGGCTATCAAACACCACCCTT
KHP_1322                   603  ACTTGAGCAGTGTCGGCTCGCCGAACTTGAGGCTTATTTAAAAGCTGCAGGGCTATCAAACACCACCCTT
HPKB_1373                  435  -------------------------------GCTTATTTAAAAGCTACAGGGCTATCAAACACCACCCTT
hp908_1453-hp908_1454      561  -------------------------------GCTTACTTAAAAGCTACGGGGCTAGAAAACACCACCCTT
HELPY_1443-HELPY_1444      561  -------------------------------GCTTATTTAAAAGCTACAGGGCTAGAAAACACCACCCTT
HPB8_59-HPB8_60            534  -------------------------------GCTTACTTAAAAGCTACAGGGCTAGAAAACACTACCCTT
HPCU_07415                 522  -------------------------------GCTTATTTAAAAGCTACAGGGCTATCAAACACCACCCTT
HPSJM_07500                540  -------------------------------GCTTATTTAAAAGCTACAGGGCTAGAAAACACCACCCTT
HPSAT_07075                72   ----------------------------------------------------------------------
HPV225_1544                579  ACTTGAG------------------------GCTTATTTAAAAGCTACAGGGCTATCAAACACCACCCTT

HP1471                     553  TCTAACGATGAAGAAAACGCCCTTAATGTTTTCAAT------AATTCTGGGGGGGGGGGGGG---TAATA
jhp1364                    562  TCTAACGATGAAGAAAATGCCCTTAATGTTTTCAAT------AATTCTGGGGGGGGGGGGGG---TAATA
HPAG1_1444                 565  TCTAGCGATGAAGAAAACGCCCTTAATGTTTTCAAT------AATTCTGG---------------TAATA
HPG27_1394                 562  TCTAACGATGAACAAAACGCCCTTAATGTTTTCAAT------AATTCTATGGGGGG---------TAATA
HPP12_1449                 583  TCTAGCGAAGAAGAAAACGCCCTTAATGTTTTCAAT------AATTCTGGGGGGGGGGGGGG---TAATA
HPSH_7535                  555  TCTAACGATGAAGAAAACGCCCTTAACCTTTTCAATGGCAAAAATTCTGGGGGGGGGGGGG----TAATA
HPF16_1367-HPF16_1368      545  TCTAACGATGAAGAAAACGCCCTTAACCTTTTTGATGGCAAAAATTCTGGGGGGGGGGGGGGGG-TAATA
HPF30_1338-HPF30_1339      574  TCTAGTGATGAAGAAAACGCCCTTAACCTTTTCAATGGCAAAAATTCTGGGGGGGGGGGGGGGG-TAATA
HPF32_1358                 598  TCTAACGATGAACAAAACGCCCTTAATGCTTTCAATGGCAAAAATTCTGGGGGGGG-----GGG-TAATA
HPF57_1386                 550  TCTAACGATGAAGAAAACGCCCTTAATGTTTTCAA-----------------------------------
KHP_1322                   673  TCTAACGATGAAGAAAACGCCCTTAACCTTTTCAATGGCAAAAATTCTGGGGGGGGGGGGGGGGGTAATA
HPKB_1373                  475  TCTAGCGATGAAGAAAACGCCCTTAACCTTTTCAATGGCAAAAATTCTGGGGGGGGGGG------TAATA
hp908_1453-hp908_1454      601  TCTAGCGATGAAGAAAACGCCCTTAACGTTTTTAACGGCAACAATTCTGGGGGGGGG--------TAATA
HELPY_1443-HELPY_1444      601  TCTA-GCGAAGA-ACAAAACGCCCTTAACGTTTTCAATGGCAACAATTCTGGGGGGGGGGG----TAATA
HPB8_59-HPB8_60            574  TCTAACGATGAAGAAAACGCCCTTAATGTTTTCAAT------AATTCTATGGGGGG---------TAATA
HPCU_07415                 562  TCTAACGATGAAGAAAACGCCCTTAACCTTTTCAA-----------------------------------
HPSJM_07500                580  TCTAGCGATGAAGAAAACGCCCTTAATGTTTTCAAT------AATTCTTGGGGGGGGGG------TAATA
HPSAT_07075                72   ----------------------------------------------------------------------
HPV225_1544                625  TCTGACGATGAAGAAAACGCCCTTAATCTTTTCAATGGCAAAAATTCTGGGGGGGGGGG------TAATA
                                                           repeat x''                 TRD2
HP1471                     614  CCCCATGCGGCTTAACATGGCAAAGCTTTAGATTAGGGGATTTGTTTGAAATT--------GAAAAAACC
jhp1364                    623  CCCCATGCGGCTTGACATGGCAACACTTCAAATTAGGGGATTTGTTTGAAATT--------GAAAAAACC
HPAG1_1444                 614  CCCCATGCGGCTTAACATGGCAAAGCTTCAAACTAGGGGATTTGTTTGAAATT--------GAAAAAACC
HPG27_1394                 617  CCCCATGCGGCTTAACATGGCAAAGCTTCAAATTAGGGGATTTGTTTGAAAAAGTG---AGTGCAAGATT
HPP12_1449                 644  CCCCATGCGGCTTAACATGGCAAAGCTTCAGATTAGGGGATTTGTTTGAAATT--------GAAAAAACC
HPSH_7535                  621  CCCCATGCAGCTTAACATGGCAAAGCTTTAGATTAGGGGATTTGTTTGAAGTGTTGTCAAGTAAGAAAAT
HPF16_1367-HPF16_1368      614  CCCCATGCGGCTTAACATGGCAAAGCTTCAAATTAGGGGATTTGTTTGAAATT--------GAAAAAACC
HPF30_1338-HPF30_1339      643  CCCCATGCGGCTTAACATGGCAAAGCTTCAAATTAGGGGATTTGTTTGAAATT--------GAAAAAACC
HPF32_1358                 662  CCCCATGCGGCTTAACATGGCAAAGCTTCAAATTAGGGGATTTGTTTGAAATT--------GAAAAAACC
HPF57_1386                 584  ----ATGCGGCTTAACATGGCAAAGCTTCAAATTAGGGGATTTGTTTGAAATT--------GAAAAAACC
KHP_1322                   743  CCCCATGCGGCTTAACATGGCAAAGCTTCAAATTAGGGGATTTGTTTGAAATTGAAAAAACCTTAAGCTT
HPKB_1373                  539  CCCCATGCGGCTTAACATGGCAAAGCTTCAAATTAGGGGATTTGTTTGAAATACGCCC---CACAAAAGC
hp908_1453-hp908_1454      663  CCCCATGCGGCTTAACATGGCAACACTTCAAATTAGGGGATTTGTTTGAAATACGCCCCACAAAAGCCTA
HELPY_1443-HELPY_1444      665  CCCCATGCGGCTTAACATGGCAACACTTCAAACTAAGGGATTTGTTTGAAATTGAAAAAACATTAAGCTT
HPB8_59-HPB8_60            629  CCCCATGCGGCTTAATGTGGCAAAGCTTCAGATTAGGGGATTTGTTTGAAGTGTTGCCATATAAAAAACG
HPCU_07415                 596  ----ATGCGGCTTAACATGGCAAAGCTTCAAATTAGGGGATTTGTTTGAAATACGCCC---CACAAAAGC
HPSJM_07500                638  CCCCATGCGGCTTAACATGGCAAAGCTTCAAAATTGTAGATATTTTTGAAGTCAAAAA---CACAAGAAA
HPSAT_07075                72   -----------------------------------------------AAAATTATTATAAAGCCAT-TAC
HPV225_1544                689  CCCCATGCGGCTTAACATGGCAAAGCTTCAAATTAGGGGATTTGTTTGAAATTGAAAA---AACCTTAAG
```

```
HP1471                    676  TTA--------AGCTTTAATAAAGACGCTTTAACGCAAGGAGAAGATTATGATTATATTACAAGGA--CT
jhp1364                   685  TTA--------AGCTTTAATAAAGACGCTTTAACGCAAGGACAAGATTACGATTATATTACAAGAA--CT
HPAG1_1444                676  TTA--------AGCTTTAATAAAGACGCTTTAACGCAAGGACAAGACTACGATTATATTACAAGGA--CT
HPG27_1394                684  TT--TAGGCAAGGGGGATAAATTTAAGGCAACTTCAAAAAGCATTACAGACACGCATAATATCCCC--CT
HPP12_1449                706  TTA--------AGCTTTAATAAAGACGCTTTAACGCAAGGACAAGACTACGATTATATTACAAGGA--CT
HPSH_7535                 691  TTATCATGCCAACACGATAAAAATCCATGACACGCAAATAGAAA-ACAGCTACCCTTA-TGTCGTG--CG
HPF16_1367-HPF16_1368     676  TTA--------AGCTTTAATAAAGACGCTTTAACGCAAGGACAAGACTACGATTATATTACAAGGA--CC
HPF30_1338-HPF30_1339     705  TTA--------AGCTTTAATAAAGACGCTTTAACGCAAGGACAAGACTACGATTATATTACAAGGA--CT
HPF32_1358                724  TTA--------AGCTTTAATAAAGACGCTTTAACACAAGGACAAGACTACGATTATATTACAAGGA--CT
HPF57_1386                643  TTA--------AGCTTTAATAAAGACGCTTTAACGCAAGGACAAGACTACGATTATATTACAAGGA--CT
KHP_1322                  813  TAATAAAGACGCTTTAACGCAAGGACAAGACTACGATTATATTACAAGGACTTCGCAAAATCAAGGCGTT
HPKB_1373                 606  CTA------TAAACTCACAAACTCTCATTTATTT--GATAGTAACGCAAAAAATCCAGTCGTTACT--AA
hp908_1453-hp908_1454     732  ------TAACCTCACAAACCCTCATTTATTT--GATAGTAATGCAAAAAATCCAGTCGTTACC--AATTC
HELPY_1443-HELPY_1444     735  TAATAAAGACGCTTTAACGCAAGGAGAAGATTATGATTATATTACAAGGACTTCGCAAAATCAAGGCGTT
HPB8_59-HPB8_60           699  CTTTGATGCTAATAAGGTTAATATTTACGCCAGC--AAAACTAAAGATACTTACCCTTATGTTGTG--CG
HPCU_07415                660  CTA------TAAACTTACAAACTCCCACTTGTTT--GATAATAGTGCAAAAAATCCAGTCGTTACT--AA
HPSJM_07500               705  T----------ATTTTAGCAAGGGATGTTGT----CAAAGATAGTGGGACAACCCCCTACTTGTGT--GC
HPSAT_07075               95   CTTATCAAACTGCACAATTACCAAAGGAAAAAACTCCAACGCACGAATTACCAGCCCTAACCGCAGGGAT
HPV225_1544               755  ------------CTTTAATAAGG-ACGCTTTAACACAAGGACAAGACTACGATTATATTACAAGGA--CT

HP1471                    736  TCGCAAAATCAAGGCGTTTTGCAAACTACAGGATTTGTC--------AATGCAGAAAAT-TTAAACCCAC
jhp1364                   745  TCGCAAAATCAAGGCGTTTTGCAAACTACAGGATTTGTC--------AATGCAGAAAAT-TTAAACCCAC
HPAG1_1444                736  TCGCAAAATCAAGGCGTTTCGCAAACCACAGGATTTGTC--------AATGCAGAAAAT-TTAAACCCAC
HPG27_1394                750  AGTTTATTGCAAAA-AGGGGAATAATGGCATTATGTATT----GG-GGTAAAAAAGGTGATTTTGAAACC
HPP12_1449                766  TCGCAAAATCAAGGCGTTTTGCAAACTACAGGATTTGTC--------AATGCAGAAAAT-TTAAACCCAC
HPSH_7535                 757  CGCTGCAACCAACA-ATGGTATAAAAGGCTTTAT-TATA----GATGACTCTACATTTGCTAATGAAAAA
HPF16_1367-HPF16_1368     736  TCGCAAAATCAAGGCGTTTTGCAAACTACAGGATTTGTC--------AATGCAGAAAAT-TTAAACCCAC
HPF30_1338-HPF30_1339     765  TCGCAAAATCAAGGCGTTTTGCAAACTACAGGATTTGTC--------AATGCAGAAAAT-TTAAACCCAC
HPF32_1358                784  TCGCAAAATCAAGGCGTTTTGCAAACTACAGGATTTGTC--------AATGCAGAAAAT-TTAAACCCAC
HPF57_1386                703  TCGCAAAATCAAGGCGTTTTGCAAACCACAGGATTTGTC--------AATGCAGAAAAT-TTAAACCCAC
KHP_1322                  883  TTGCAAACCACAGGATTTGTCAATGCAGAAAATTTAAACCCACCATTTACTTGGAGTTTAGGGCTTTTAC
HPKB_1373                 666  TTCTAGTTTGAATA-ATGGGATAAGCGGTTATTCTTCTT----TA-GAACCCACCGAAAAGGGTAATCAA
hp908_1453-hp908_1454     793  TAGTTTGAATA-ATGGGATAAGCGGTTATTCTTCTT----TA-GAACCCACCGAAAAGGGCAATCAGATC
HELPY_1443-HELPY_1444     805  TTGCAAACCACAGGATTTGTCAATGCAGAAAATTTAAACCCACCATTTACTTGGAGTTTAGGGCTTTTGC
HPB8_59-HPB8_60           765  CACAAGCCTAAATA-ATGGCATAAGGGGTTATTTAAAAGAAAATACAAATTTCTTAAATGCGGGAAACAC
HPCU_07415                720  TTCTAGTTTGAATA-ATGGGATAAGCGGTTATTCTTCTT----TA-GAACCCACCGAAAAGGGTAATCAA
HPSJM_07500               759  TAGTAAAGAGAATA-ATGCTGTAAATTCTTATATTAATTACAATGCGGATTTTTTGGAT--AAGGGCAAT
HPSAT_07075               165  TTTAAATCAAGGGTTAAATAATT-TTGTGCCAAAAGAAAATGCCACGATTTTAAAAAATGTTATTTCAAT
HPV225_1544               811  TCGCAAAATCAAGGCGTTTTGCAAACCACAGGATTTGT--CAATGCAGAAAATTTAAACCCACCATTTAC

HP1471                    797  CATTTACTTGGAGTTTAGGGCTTTTGCAAATGGATTTTTTCTATCGTAAAAAGTCATGGTATGCGGGACA
jhp1364                   806  CATTTACTTGGAGTTTAGGGCTTTTGCAAATGGATTTTTTCTATCGTAAAAAGTCATGGTATGCGGGACA
HPAG1_1444                797  CATTTACTTGGAGTTTAGGGCTTTTGCAAATGGATTTTTTCTATCGTAAAAAGTCATGGTATGCGGGACA
HPG27_1394                814  TATAATAATATCATTTCTATTATCTA-TAATGGCGTAATTGCTACAGGATTAACTTATGCCCATAGA-GA
HPP12_1449                827  CATTTACTTGGAGTTTAGGGCTTTTGCAAATGGATTTTTTCTATCGTAAAAAGTCATGGTATGCGGGACA
HPSH_7535                 821  AATACCC-TTTCGTTCGCGCAAGACA-CTTTCACCGTGTTCTATCAAAAACAACCTTATTTTACAGGCAA
HPF16_1367-HPF16_1368     797  CATTTACTTGGAGTTTAGGGCTTTTACAAATGGATTTTTTCTATCGTAAAAAGTCATGGTATGCGGGACA
HPF30_1338-HPF30_1339     826  CATTTACTTGGAGTTTAGGGCTTTTACAAATGGATTTTTTCTATCGTAAAAAGTCATGGTATGCGGGACA
HPF32_1358                845  CATTTACTTGGAGTTTAGGGCTTTTGCAAATGGATTTTTTCTATCGTAAAAAGTCATGGTATGCGGGACA
HPF57_1386                764  CATTTACTTGGAGTTTAGGGCTTTTACAAATGGATTTTTTCTATCGTAAAAAGTCATGGTATGCGGGACA
KHP_1322                  953  AAATGGATTTTTTCTATCGTAAAAAGTCATGGTATGCGGGACAATTCATGCGAAAAATCACACCAAAAAC
HPKB_1373                 730  ATCACTTATAGCGATACCACGACTT--CAGAGGGGATTTTTTACCAAAAAAGACCT---TTTATAGGGTA
hp908_1453-hp908_1454     857  ACTTATAGCGATACCACGACTT--CAGAGGGTATTTTTTATCAAAAAAGACCT---TTTATAGGGTAT--
HELPY_1443-HELPY_1444     875  AAATGGATTTTTTCTATCGTAAAAAATCATGGTATGCGGGACAATTTATGCGAAAAATCACGCCAAAAGC
HPB8_59-HPB8_60           834  TATATCTTTTGGG----CAAGACAC---CGCTACAATGTTTTATCAAGAAAAGCCTTATTTCACAGGGGA
HPCU_07415                784  ATCACTTATAGCGATACCACGACTT--CAGAGGGGATTTTTTATCAAAAAAGACCT---TTTATAGGGTA
HPSJM_07500               826  TGTATTTTTATTGGTGGTAAAACTT--TTGTTGTTACATACCAACAAAAAGACTTT---TATTCAAACGA
HPSAT_07075               234  TTCTGCTAATGGGGCTAACACCGGGGCTACATTTTACCAACCTCATGAATTTTGCGTATTACAAGACGCT
HPV225_1544               879  TTGGAGTTTAGGGCTTTTGCAAATG--GATTTTTTCTATCGTAAAAAGTCATGGTA---TGCGGGACAGT
```

```
HP1471                   867  ATTCATGCGAAAAATCACACCAAAAAC-----TGAAATTGAAAATAAAATTGATTTACGCATAGCCAACT
jhp1364                  876  ATTCATGCGAAAAATCACACCAAAAAC-----TGAAATTAAAAATAAAATTAATTCACGCATAGCCCACT
HPAG1_1444               867  ATTCATGCGAAAAATCACACCAAAAAC-----TGAAATTGAAAATAAAATTGATTTACGCATAGCCCACT
HPG27_1394               882  TGAGGTAGGGATTTTAG--CTGAAAGCTATTTTATAAAATTTAAAAATGGTAACCCTAATTTTTTATGTA
HPP12_1449               897  ATTCATGCGAAAAATCACACCAAAAAC-----TGAAATTGAAAATAAAATTAATTTACGCATAGCTCACT
HPSH_7535                889  TAAGGTTAAAATTTTAAAACCAAAATTTGCTTTCAAGAGTCCTAAAAT-------TTTATATTTTATA-A
HPF16_1367-HPF16_1368    867  ATTCATGCGAAAAATCACGCCAAAAAC-----TGAAATTGAAAATAAAATTAATTCACGCATAGCCCACT
HPF30_1338-HPF30_1339    896  ATTCATGCGAAAAATCACACCAAAAAC-----TGAAATTGAAAATAAAATTAATTTACGCATAGCCCACT
HPF32_1358               915  ATTCATGCGAAAAATCACACCAAAAAC-----TGAAATTGAAAATAAAATTAATTCACGCATAGCCCACT
HPF57_1386               834  ATTCATGCGAAAAATCACACCAAAAAC-----TGAAATTGAAAATAAAATTAATTTACGCATAGCCCACT
KHP_1322                 1023 TGAAATTGAAAATAAAATTAATTCACGCATAGCCCACTACTTCACAACGCTTTTAAACGCCTTAAAACGC
HPKB_1373                795  T----TCGCATGTGCAAGGGCTGTATT--CTTTAAAATACCATGAGTT-------TTGGAATGAAAAAAC
hp908_1453-hp908_1454    919  --TCGCATGTGCAAGGGCTATACC--CTTTAAAACACCATGAGTT-------TTGGAATGAAAAAACTTT
HELPY_1443-HELPY_1444    945  TGAAATCAAAAATAAAATTAATTCGCGCGCAGCCCACTATTTCACAACGCTTTTAAACGCCTTAAAACGC
HPB8_59-HPB8_60          897  TA-------AAATCAAAATTCTACGAT-----GCAAAAATCCTAATTT--------TAATAAGATAAATG
HPCU_07415               849  T----TCGCATGTGCAAGGGCTGTATT--CTTTAAAATACCATGAGTT-------TTGGAATGAAAAAAC
HPSJM_07500              891  TA---GC-CATAATTTAGCTTTGTATC----TTAAAGACACGCATTCAA--AAACCAAACTTAACCAACT
HPSAT_07075              304  TACGCTATTGAATTTATTGGCGATAAAAAGCTTAACGATAAAGAATATTTATTTTTTGTATG---TGCTA
HPV225_1544              944  TC---ATGCGAAAAATCACACCAAAAA----CTGAAATTGAAAATAAAATTAATTCACGCATAGCCCACT

HP1471                   932  ACTTCACAACGCTTTTAAACGCCTTAAAACGCCCTTTATTAAGCGTATTGGTTAGAGATATTGATAAAAC
jhp1364                  941  ATTTCACAACGCTTTTAAACGCCTTAAAACGCCCTTTATTGAGTGTATTAGTTAGGGATATTGATAAAAC
HPAG1_1444               932  ATTTCACAACGCTTTTAAACGCCCTAAAACGCCCTTTATTAAGCGTATTGGTTAGAGATATTGATAAAAC
HPG27_1394               950  ACTTATTTATAAAAACAGCGATTGAAAAAGTTTTATACCCAAAA-TATTCACGAGACAATTTAGCGACAT
HPP12_1449               962  ATTTCACAACGCTTTTAAACGCCTTAAAACGCCCTTTATTAAGCGTATTGGTTAGAGATATTGATAAAAC
HPSH_7535                951  GCGCGATTTTACAATT--TATTTTAAAACCCTTAACTTGGGGGC-TAGGCTCTACAACAGAAAGCATTGC
HPF16_1367-HPF16_1368    932  ATTTCACAACGCTTTTAAACGCCTTAAAACGCCCTTTATTAAGCGTATTGGTTAGAGATATTGATAAAAC
HPF30_1338-HPF30_1339    961  ATTTCACAACGCTTTTAAACGCCTTAAAACGCCCTTTATTAAGCGTATTGGTTAGAGATATTGATAAAAC
HPF32_1358               980  ACTTCACAACGCTTTTAAACGCCTTAAAACGCCCTTTATTAAGCGTATTGGTTAGAGATATTGATAAAAC
HPF57_1386               899  ATTTCACAACGCTTTTAAACGCCTTAAAACGCCCTTTATTAAGCGTATTGGTTAGAGATATTGATAAAAC
KHP_1322                 1093 CCTTTATTAAGCGTATTGGTTAGAGATATTGATAAAACTTTTAGGGAGCAAAAAATCCAA----------
HPKB_1373                852  TTTACTCTATATCGTTACTGCTTTTAAAAAAGTAGCTTGCGGGCGTTTTAATTATGGTAACAAATTTAAC
hp908_1453-hp908_1454    979  ACTTTATATCGTTGTAGCTTTTAAAAAAGTAGCTTGTGGGCGTTTTGATTATGGTAATAAATTCAATCG-
HELPY_1443-HELPY_1444    1015 CCTTTATTGAGTGTATTAGTTAGGGATATTGATAAAACTTTTAGGGAGCAAAAAATCCAA----------
HPB8_59-HPB8_60          947  CTTTATTTTTTATCACAAG---TTTAACAAAGGCTTTTAGAAATTTTTCATGGGGGAGTGCGAGTTTTAG
HPCU_07415               906  TTTACTCTATATCGTTACTGCTTTTAAAAAAGTAGCTTGTGGGCGTTTTAATTATGGTAACAAATTTAAC
HPSJM_07500              951  ATTTATTATTACTTGCA-----TTTATAAAGCTTTAAATAATAAATATT--CTTGGGGTGATAGCATAAG
HPSAT_07075              371  TTTCAAAAGTTATTTATAA---TAATAGTAAATACGAATGGACAAATAAAGCAGGGTGGAATAAAGTTAA
HPV225_1544              1007 ACTTCACAACGCTTTTAAACGCCTTAAAACGCCCTTTATTAAGCGTATTGGTTAGAGATATTGATAAAAC

HP1471                   1002 TTTTAGGGAGCAAAAAATCCAACTACCCCTAAAACCCACCGCTAAAACTCAAACCCTTGATGGTATTGAT
jhp1364                  1011 TTTTAGGGAGCAAAAAATCCAACTACCCCTAAAACCCACCGCTAAAACTCAAAGCCTTGATGGTATTGAT
HPAG1_1444               1002 TTTTAGGGAGCAAAAAATCCAACTACCCCTAAAACCCACCGCTAACACTCAAACCCTTGAGGATATTGAT
HPG27_1394               1019 GGACTAATAAAGTAGAGAACGA------GTTAATCATTCTCCC--CACCAACCCACATGGCGGTATTGAT
HPP12_1449               1032 TTTTAGGGAGCAAAAAATCCAACTACCCCTAAAACCCACCGCTAACGCTCAAACCCTTGATGGTATTGAT
HPSH_7535                1018 GGAATTTAAATTTTCTCTACCC------CTAAAACCCACCGCTAACACTCAAACCCTTAAGGATATTGAT
HPF16_1367-HPF16_1368    1002 TTTTAGGGAGCAAAAAATCCAACTACCCCTAAAACCCACCGCTAACACTCAAACCCTTGATGGTATTGAT
HPF30_1338-HPF30_1339    1031 TTTTAGGGAGCAAAAAATCCAACTACCCCTAAAACCCACCGCTAAAACTCAAACCCTTAAGGATATTGAT
HPF32_1358               1050 TTTTAGGGAGCAAAAAATCCAACTACCCCTAAAACCCACCGCTAAAACTCAAACCCTTGATGGTATTGAT
HPF57_1386               969  TTTTAGGGAGCAAAAAATCCAATTACCCCTAAAACCCACCGCTAAAACTCAAACCCTTGATGGTATTGAT
KHP_1322                 1152 ----------------------CTACCCCTAAAACCCACCGCTAACACTCAAACCCTTGATGATATTGAT
HPKB_1373                922  CG-AAAAATTGCTAGCGGAATG-------CCAATCTTTCTCCC--CACCAACCAACATGGCGGAATTGAT
hp908_1453-hp908_1454    1047 -TAAAATTGCTAGTGAAATGTC-----------AATCATTCTCCCCACCAACCCACATGGCGGTATTGAT
HELPY_1443-HELPY_1444    1074 ----------------------CTACCCCTAAAACCCACCGCTAACACTCAAACCCTTGAGGATATTGAT
HPB8_59-HPB8_60          1014 TG---TTAGCATAATAGAAAAT------CAAAATATTTCACTCCCCACCAACCCGCATGGCAAAATAGAT
HPCU_07415               976  CG--AAAAATTGCTAGTGGAAT------GCCAATCATTCTCCC--CACCAACCAACATGGCAAAATAGAT
HPSJM_07500              1014 TAACACAAAGATACAAAATGAT---------AGCATTCTACTACCCACCAACCCGCATGGCAAAATAGAT
HPSAT_07075              438  AAACGAGTTAATTTCT---CTACCCCTAAAACCCACCGCTAACACTCAAACCCTTGATGG---TATTGAT
HPV225_1544              1077 TTTTAGGGAGCAAAAAATCCAACTACCCCTAAAACCCACCGCTAACACTCAAACCCTTGATGGTATTGAT
```

```
                            repeat y''       conserved region
HP1471                      1072 TTTGATTTCATGCACACCCTTATCAACGCCCTAATGAAGCAAACCATTCAAGGCGTGGCTCAATACTGCG
jhp1364                     1081 TTTGATTTCATGCACACCCTAATCAACGCCCTGATGAAGCAAACCATTCAAGGCGTGGTTCAATACTGCG
HPAG1_1444                  1072 TTTGATTTCATGCGCACCCTTATCAACGCCCTGATGAAGCAAACCATTCAAGGCGTGGCTCAATACTGCG
HPG27_1394                  1081 TTTGATTTCATGCACACCCTAATCAACGCTCTGATGAAACAAATTATTCAAGGCGTGGCTCAATACTGCG
HPP12_1449                  1102 TTTCATTTCATGAGCACCCTTATAAACGCTCTAATGAAGCAAACCATTCAAGGCGTGGCTCAATATTGCA
HPSH_7535                   1082 TTTCATTTCATGCGCACCTTTATCAACGCCCTAATGAAACAAACCATTCAAGGCGTGGTTCAATACAGCA
HPF16_1367-HPF16_1368       1072 TTTCATTTCATGCACACCCTTATAAACGCTCTAATGAAACAAACCATTCAAGGCGTGGTTCAATACAGCA
HPF30_1338-HPF30_1339       1101 TTTCATTTCATGCGCACCTTTATAAACGCTCTAATGAAGCAAACCATTCAAGGCGTGGTTCAATACAGCA
HPF32_1358                  1120 TTTCATTTCATGCACACCCTTATAAACGCTCTAATGAAACAAACCATTCAAGGCGTGGTTCAATACAGCA
HPF57_1386                  1039 TTTCATTTCATGCACACCTTTATAAACGCCCTAATGAAACAAACCATTCAAGGCGTGGTTCAATACAGCA
KHP_1322                    1201 TTTCATTTCATGCGCACCTTTATAAACGCTCTAATGAAGCAAACCATTCAAGGCGTGGTCCAATACAGCA
HPKB_1373                   982  TTTCATTTCATGCACACCTTTATAAACGCTCTAATGAAACAAACCATTCAAGGCGTGGTTCAATACAGCC
hp908_1453-hp908_1454       1106 TTTACTTTCATGCGCACCCTAATTAACGCCCTAATGAAACAAACCATTCAAGGCGTGGTTCAATACAGCA
HELPY_1443-HELPY_1444       1123 TTTCATTTCATGCACACCCTCATCAACGCCCTGATGAAACAAACCATTCAGGGCGTGGCTCAATACTGCG
HPB8_59-HPB8_60             1075 TTTAACTTCATGCGCACCCTAATCAACGCCCTAATGAAACAAACCATTCAGGGCGTGGTTCAATACAGCG
HPCU_07415                  1036 TTTCATTTCATGCGCACCTTTATCAACGCTCTAATGAAACAAACCATTCAAGGCGTGGTTCAATACAGCA
HPSJM_07500                 1075 TTTGATTTCATGCACACCCTAATCAACGCCCTAATGAAGCAAACCATTCAAGGCGTGGCTCAATACTGCA
HPSAT_07075                 502  TTTCATTTCATGCGCACCTTTATCAACGCTCTAATGAAACAAACCATTCAAGGCGTGGTTCAATACAGCA
HPV225_1544                 1147 TTTCATTTCCATCGCACCTTTATAAACGCCCTAATGAAACAAACCATTCAAGGCGTGGTTCAATACAGCA

HP1471                      1142 ACGCTAAAATACAAGCTACAAAAGAGGTTATCAGCCAAGAAGCGCCCGTTCAAAAAGACTCGTTATTTTA
jhp1364                     1151 ACGCTAAAATACAGGCTACAAAAGAAGTTATCAGCCAAGAAACGCCTATTCAAAAAGACTCGTTATTTTG
HPAG1_1444                  1142 ACGCTAAAATACAGGCTACAAAAGAAGCCATCAGCCAAGAAACACCCATTCAAAAAGACTCGTTATTTTG
HPG27_1394                  1151 GCGCTAAAATCCAAGCCACAAAAGAAGTTATCAGCCAAGAAACACCCGTTCAAAAAGACTCGTTATTTTG
HPP12_1449                  1172 GCGCTAAAATTCAGGCCACAAAAGAAGTTATCAGCCAAGAAACACCCATTCAAAAAGACTCGTTGTTTTG
HPSH_7535                   1152 GCGCTAAAATACAGGCTACAAAAGAGGCCATCAGCCAAGAAACGCCAACCCAAAAAGACTCGTTGTTTTG
HPF16_1367-HPF16_1368       1142 GCGCTAAAATACAAGCTACAAAAGAGGCCATCAGCCAAGAAACACCAACCCAAAAAGACTCGTTATTTTG
HPF30_1338-HPF30_1339       1171 GCGCTAAAATACAGGCTACAAAAGAAGCCATCAGCCAAGAAACACCAACCCAAAAAGACTCGTTATTTTG
HPF32_1358                  1190 GCGCTAAAATACAAGCTACAAAAGAAGCCATCAGCCAAGAAGCGCCAACCCAAAAAGACTTGTTATTTTG
HPF57_1386                  1109 ACGCTAAAATACAAGCTACACAAGAGGCCATCAGCCAAGAAACACCAATCCAAAAAGACTCGTTATTTTG
KHP_1322                    1271 GCGCTAAAATACAAGCTACAAAAGAAGCCATCAGCCAAGAAGCGCCAGCCCAAAAAGATTCGTTATTTTG
HPKB_1373                   1052 GCGCTAAAATACAAGCCACAAAAGAAACCATCAGCCAAGAAACACCAACCCAAAAAGACTCGTTATTTTG
hp908_1453-hp908_1454       1176 GCGCTAAAATCCAGGCCGCAAAAGAAGTTATTAACCAAGAAACGCCCGTTCAAAAAGACTCGTTATTTTG
HELPY_1443-HELPY_1444       1193 ACGCTAAAATCCAGGCCACAAAAGAAATCATCAGCCAAGAAACACCCATTCAAAAAGACTCGTTATTTTG
HPB8_59-HPB8_60             1145 GCGCTAAAATTCAGGCTACAAAAGAAGCCATCAGTCAAGAAACACCCGTTCAAAAAGACTCGTTATTTTA
HPCU_07415                  1106 GCGCTAAAATACAGGCCACAAAAGAAGCCATCAGCCAAGAAACGCCAACCCAAAAAGACTCGTTATTTTG
HPSJM_07500                 1145 GCGCTAAAATCCAAGCCACAAAAGAAATCATCAGCCAAGAAACACCCGTTCAAAAAGACTCGTTATTTTG
HPSAT_07075                 572  GCGCTAAAATACAGGCCACAAAAGAAGCCATCAGCCAAGAAACGTCAACCCAAAAAGACTCGTTGTTTTG
HPV225_1544                 1217 GCGCTAAAATACAGGCTACAAAAGAAGCCATCAGCCAAGAAACGCCAACCCAAAAAGACTTGTTATTTTG

HP1471                      1212 A
jhp1364                     1221 A
HPAG1_1444                  1212 A
HPG27_1394                  1221 A
HPP12_1449                  1242 A
HPSH_7535                   1222 A
HPF16_1367-HPF16_1368       1212 A
HPF30_1338-HPF30_1339       1241 A
HPF32_1358                  1260 A
HPF57_1386                  1179 A
KHP_1322                    1341 A
HPKB_1373                   1122 A
hp908_1453-hp908_1454       1246 A
HELPY_1443-HELPY_1444       1263 A
HPB8_59-HPB8_60             1215 A
HPCU_07415                  1176 A
HPSJM_07500                 1215 A
HPSAT_07075                 642  A
HPV225_1544                 1287 A
```
